# Transcriptome and Metabonomic Analysis of *Tamarix ramosissima* Potassium (K^+^) Channels and Transporters in Response to NaCl Stress

**DOI:** 10.3390/genes13081313

**Published:** 2022-07-23

**Authors:** Yahui Chen, Shiyang Zhang, Shanfeng Du, Jiang Jiang, Guangyu Wang

**Affiliations:** 1 Collaborative Innovation Center of Sustainable Forestry in Southern China of Jiangsu Province, Nanjing Forestry University, Nanjing 210037, China; cyh01@student.ubc.ca (Y.C.); chocho.njfu@gmail.com (S.D.); 2 Faculty of Science and Department of Forest Resources Management, University of British Columbia, Vancouver, BC V6T 1Z4, Canada; zhshiyan@student.ubc.ca

**Keywords:** potassium channel, potassium ion transporter, NaCl stress

## Abstract

Potassium ion (K^+^) channels and transporters are key components of plant K^+^ absorption and transportation and play an important role in plant growth and development. This study revealed that K^+^ channels and transporters are involved in the salt tolerance molecular mechanism and metabolites of the halophyte representative plant *Tamarix* *ramosissima* (*T*. *ramosissima*) in response to NaCl stress, providing a theoretical basis for the mitigation of salt stress using halophytes. Through transcriptome sequencing and metabolite detection analysis of 0 h, 48 h and 168 h by applying exogenous K^+^ to the roots of *T*. *ramosissima* under NaCl stress, 15 high-quality Clean Data bases were obtained, Q20 reached more than 97%, Q30 reached more than 92%, and GC content reached 44.5%, which is in line with further bioinformatics analysis. Based on the Liquid chromatography–mass spectrometry (LC-MS) analysis, the roots of *T*. *ramosissima* were exposed to exogenous potassium for 48 h and 168 h under NaCl stress, and 1510 and 1124 metabolites were identified in positive and negative ion mode, respectively. Through orthogonal projections to latent structures discriminant analysis (OPLS-DA) model analysis, its metabolomic data have excellent predictability and stability. The results of this study showed that there were 37 differentially expressed genes (DEGs) annotated as Class 2 K^+^ channels (Shaker-like K^+^ channel and TPK channel) and Class 3 K^+^ transporters (HAK/KUP/KT, HKT and CPAs transporter families). Among them, 29 DEGs were annotated to the gene ontology (GO) database, and the most genes were involved in the GO Biological Process. In addition, the expression levels of *Unigene0014342* in the HAK/KUP/KT transporter and *Unigene0088276* and *Unigene0103067* in the CPAs transporter both first decreased and then increased when treated with 200 mM NaCl for 48 h and 168 h. However, when treated with 200 mM NaCl + 10 mM KCl for 48 h and 168 h, a continuous upward trend was shown. Notably, the expression level of *Unigene0016813* in CPAS transporter continued to increase when treated with 200 mM NaCl and 200 mM NaCl + 10 mM KCl for 48 h and 168 h. 3 DEGs, *Unigene0088276*, *Unigene0016813* and *Unigene0103067*, were dominated by the positive regulation of their related metabolites, and this correlation was significant. The results showed that these DEGs increased the absorption of K^+^ and the ratio of K^+^/Na^+^ under NaCl stress at 48 h and 168 h after adding exogenous potassium and enhanced the salt tolerance of *T*. *ramosissima*. Notably, the expression level of *Unigene0103067* in the CPAs transporter was consistently upregulated when 200 mM NaCl + 10 mM KCl was treated for 48 h and 168 h. The positive regulatory metabolites were always dominant, which better helped *T*. *ramosissima* resist salt stress. *Unigene0103067* plays an important role in enhancing the salt tolerance of *T*. *ramosissima* and reducing the toxicity of NaCl in roots. Additionally, phylogenetic tree analysis showed that *Unigene0103067* and *Reaumuria* *trigyna* had the closest genetic distance in the evolutionary relationship. Finally, 9 DEGs were randomly selected for quantitative real-time PCR (qRT-PCR) verification. Their expression trends were completely consistent with the transcriptome sequencing analysis results, proving that this study’s data are accurate and reliable. This study provides resources for revealing the molecular mechanism of NaCl stress tolerance in *T*. *ramosissima* and lays a theoretical foundation for cultivating new salt-tolerant varieties.

## 1. Introduction

K^+^, the most abundant cation in plant cells, is also one of the three essential mineral nutrients for plant growth and development [1]. It is involved in many important physiological and biochemical pathways in plants, such as promoting plant fat metabolism and water metabolism, promoting assimilation transformation and nutrient transport in phloem, controlling cell membrane polarization, regulating stomatal movement and promoting photosynthesis, functioning as an activator of enzymes, maintaining anion and cation balance, adjusting osmotic pressure, etc. [2,3,4]. Simultaneously, K^+^ can improve plants’ tolerance to abiotic stresses such as drought, salt and heavy metals. Furthermore, it can also resist biotic stresses such as fungi [5]. Potassium deficiencies in the soil will directly affect the growth and development of plants, such as by accelerating the yellowing of old leaves, short roots and easy lodging, etc. [2]. About one-third of the world’s irrigated land is affected by soil salinization. In saline soil, a high concentration of Na^+^ competes with plants to absorb K^+^, K^+^ leakage through outwardly rectifying K^+^ channels, decreases the K^+^/Na^+^ ratio, and causes Na^+^ poisoning and biochemical metabolic disorders in plants, resulting in inhibitions in plant growth or even death [6,7]. Therefore, increasing K^+^ uptake and reducing Na^+^ accumulation in plants is one of the main strategies for resisting salt stress [6,8].

Plants take up K^+^ in the root epidermis through K^+^ transporters and K^+^ channels [9,10]. In the early 1960s, Epstein et al. [11] proposed 2 K^+^ transmembrane absorption mechanisms, a high-affinity transport system (HATS) and a low-affinity transport system (LATS). HATS is in the range of external K^+^ concentration < 0.2 mM, completed by H^+^/K^+^ co-transport, and can start quickly when the outside world is low in potassium [11,12]. LATS occurs when the external K^+^ concentration is ≥0.3 mM, acts through ion channels and is insensitive to external K^+^ concentration [13,14,15]. The concentration of K^+^ in the soil solution is variable (0.01–1 mM) to maintain a relatively high and stable K^+^ content (100–150 mM) in the plant cytoplasm [16]. The acquisition of K^+^ from the soil by plant roots depends on the transport of K^+^ in plant cells, which is a combination of HATS and LATS [17]. To date, in the model plant *Arabidopsis thaliana*, more than 70 K^+^ transporters and K^+^ channels have been involved in the plant potassium nutrition’s uptake and transport mechanism. According to the structure and function of K^+^ transporters and K^+^ channel proteins, these can mainly be divided into three types of K^+^ channels and three types of K^+^ transporters, namely, Shaker-like K^+^ Channel (Shaker channel), Tandem-Pore K^+^ (TPK Channel) channel, K^+^ Inward Rectifier-Like Channel (Kir-like Channel), high-affinity K^+^ transporter/K^+^ uptake permease/K^+^ transporter (HAK/KUP/KT), high-affinity K^+^ transporter (HKT) and cation–protein antiporters (CPAs) transporters [4,18,19]. Generally, plants adapt to saline environments by maintaining the K^+^/Na^+^ ratio in the cytoplasm. Ma et al. [20] found that the Shaker channel AKT1 can respond to salt stress in plants, and the AKT1 protein just maintains this ratio, thereby enhancing the salt tolerance of plants. In the HAK/KUP/KT transporter, both Cluster III and Cluster IV family proteins are expressed in the roots of plants. This helps high-affinity K^+^ absorption and transportation, and it can also participate in Na^+^ transport. Chen et al. [21] reported that *AtHAK11* is a member of Cluster III of HAK/KUP/KT. Under salt stress, its expression in Arabidopsis roots is significantly upregulated, and it may be involved in the regulation mechanism of plant salt tolerance. HKT1 is one of the most important Na^+^ transporters, which is usually expressed in xylem parenchyma cells, and HKT1 plays an integral role in the salt tolerance mechanism of both monocotyledonous and dicotyledonous plants [22,23,24]. Chen [25] and Li [26] et al. found that, under saline–alkali stress, soybean *GmHKT1* and *GmHKT4* can mediate K^+^ uptake in plant roots. While the overexpression of *GmHKT1* and *GmHKT4* can make transgenic soybeans accumulate more K^+^ and exclude excess Na^+^, which significantly improved the salinity tolerance of transgenic soybean, it plays an extremely important role in maintaining the dynamic balance between K^+^/Na^+^ ratio and osmotic potential in soybean plants. The CPAs transporter forms a large family including cation/proton reverse transport in plants. For example, antiporters such as Na^+^/H^+^ and K^+^/H^+^ are essential to maintaining Na^+^/H^+^ homeostasis in plants and improving plant salt tolerance [27]. According to differences in K^+^ transporter protein structure and transport function, the CPAs transporter can be divided into three subfamilies: Na^+^/H^+^ exchanger (NHX), Cation/H^+^ exchanger (CHX), K^+^ exchanger antiporter (KEA) [28]. In *Arabidopsis*, 8 NHX family genes have successfully been cloned. Among them, *AtNHX1* is located in the plant tonoplast. It has Na^+^/H^+^ reverse transport functions, and the overexpression of *AtNHX1* can improve the salt tolerance of transgenic plants. *AtNHX1* also plays an important role in regulating pH and osmotic potential in plant cells [29]. The research results of Zhizhong Song et al. [30,31] found that the addition of exogenous 10 mM KCl could effectively alleviate the toxic effect of drought stress on the growth of *Alternanthera philoxeroides* and enhanced K^+^ enrichment level in plants. They also found that the overexpression of *ApKUP4* in Arabidopsis significantly enhanced the K^+^ enrichment level and ROS scavenging ability of transgenic plants under NaCl stress conditions, thereby improving the tolerance of transgenic plants to NaCl stress.

*Tamarix ramosissima* (*T*. *ramosissima*) is a halophyte that secretes halophytes and has developed an efficient abiotic stress tolerance system to adapt to unfavorable environments for its long-term survival and evolution [32]. It has been reported that halophytes have the ability to retain more K^+^ under salt stress conditions, and their absorption and transportation of K^+^ depend on a variety of K^+^ transporters that can adapt to different saline-alkali conditions [33]. Simultaneously, the K^+^ requirements of halophytes can efficiently be taken up from soil solutions by the roots and further transferred to the aerial parts, before being intracellularly distributed to different compartments and satisfied by various K^+^ (Na^+^) transport systems. In addition, Lu Yan et al. [34] found that low concentration (≤100 mM) NaCl stress can promote the growth of *T*. *ramosissima*, while high concentration (≥200 mM) NaCl inhibits its growth. In this study, *T*. *ramosissima* was used as the research object, and 200-mM NaCl and 200-mM NaCl + 10 mM KCl treatments were set. Samples were taken at 0h, 48 h and 168 h, respectively, and transcriptomic and metabolomic analysis methods were used to mine of K^+^ channel and transporter genes in response to NaCl stress in *T*. *ramosissima.* The application of exogenous K^+^ alleviated the change patterns of response genes and their metabolites under NaCl stress and was combined with quantitative real-time PCR (qRT-PCR) to verify the expression levels of differentially expressed genes (DEGs). This study provides a foundation for revealing the molecular mechanisms of applying exogenous K^+^ to alleviate NaCl toxicity in *Tamarix* plants and lays a theoretical foundation for cultivating new salt-tolerant varieties.

## 2. Materials and Methods

### 2.1. Plant Material

Plant *T*. *ramosissima* seedlings were supplied by the Dongying Experimental Base of the Shandong Academy of Forestry Sciences. From October 2019 to May 2021, the experiment was conducted and completed at the Key Laboratory of the Ministry of Education, School of Forestry, Nanjing Forestry University. Five-month-old *T*. *ramosissima* seedlings with similar growth were selected and transferred to a 24-hole hydroponic box (40 cm × 30 cm × 16 cm) filled with 1/2 Hoagland nutrient solution. Then, they were placed in a greenhouse with a temperature of 26 ± 2 °C and relative humidity of 40% to 55%, and cultivated for 2 months. The culture solution was replaced every 3 days.

### 2.2. Plant Material Treatment

Control and treatment groups were set in each experiment, with 8 plants in each group. The experiment was repeated 3 times in total and cultured with 1/2 Hoagland nutrient solution as the control group (CK), and 1/2 Hoagland nutrient solution supplemented with 200 mM NaCl and 1/2 Hoagland nutrient solution cultured with 200 mM NaCl + 10 mM KCl as treatment groups, changing the culture medium every 3 days *T*. *ramosissima* root samples were collected at 0h, 48 h, and 168 h of treatment, respectively, and immediately placed in liquid nitrogen for processing and then transferred to a −80 °C refrigerator for storage.

### 2.3. Transcriptome Sequencing and Differentially Expressed Genes (DEGs) Screening

After treatment with liquid nitrogen, the *T*. *ramosissima* root samples were sent to Guangzhou GENE Denovo Company for 3-generation high-throughput transcriptome sequencing. Referring to the transcriptome sequencing method of Chen et al. [35], Illumina raw sequencing data were submitted to the National Center for Biotechnology Information (NCBI) Short Reads Archive (SRA) database; the SRP number is SRP356215. The reads count data obtained by sequencing were analyzed according to DESeq2 software [36] to obtain the final correct FDR value (FDR value is the *p*-value value after BH correction), and a corrected *p* < 0.05 was considered significantly enriched. We screened genes with FDR < 0.05 and |log2FC| > 1 as significant DEGs based on the differential analysis results. Finally, the DEGs for gene ontology (GO) and the Kyoto Encyclopedia of Genes and Genomes (KEGG) enrichment analysis were obtained using the GO database [37] and Release 93.0 [38], respectively.

### 2.4. Metabolic Extraction, Detection and Differential Metabolite Screening

After treatment with liquid nitrogen, *T*. *ramosissima* root samples were sent to Guangzhou GENE Denovo Company for metabolite extraction and detection. A total of 0.1 g of *T*. *ramosissima* root samples ground in liquid nitrogen were selected as experimental samples. These were put in an EP tube and 500 μL of 80% methanol in water was added and oscillated with a vortex. After standing in an ice bath for 5 min, this was centrifuged at 15,000× *g* for 20 min at 4 °C. Then, a certain amount of supernatant was taken and mass spectrometer-grade water was added to dilute the solution to a methanol content of 53%. Finally, this was centrifuged at 15,000× *g* for 20 min at 4 °C; then, the supernatant was collected and injected into Liquid chromatography–mass spectrometry (LC-MS) for analysis. An equal volume of sample was taken from each experimental sample and mixed as QC samples; 53% methanol aqueous solution was used instead of an experimental sample as a blank sample. Baseline filtering, peak identification, integration, retention time correction, peak alignment and normalization were obtained using Progenesis QI (http://www.nonlinear.com/progenesis/qi/, accessed on 7 January 2021) software from raw data after mass spectrometry, the final result is a data matrix containing retention times, mass-to-charge ratios and peak intensities. The obtained peaks were analyzed with progenesis QI software and database; all normalized data were log-transformed into the centralized format, and orthogonal projections to latent structures discriminant analysis (OPLS-DA) was analyzed using Simca software. Differential metabolites were screened according to the VIP value of the first principal component of the OPLS-DA model > 1.0 and *p* < 0.05 of the *t*-test [39].

### 2.5. Quantitative Real-Time PCR (qRT-PCR) Validation

9 DEGs were randomly selected to verify the accuracy of RNA-Seq results. Omega kit (Beinuo Bio, Shanghai, China) was used to extract the total RNA from root samples of control and treatment groups. RNA was reverse-transcribed into cDNA using the PrimerScript^TM^ RT Master Mix (Perfect Real Time) kit (Bao Bio, Dalian, China). Primers were designed for key DEGs and detected by qRT-PCR (Table 1). The cDNA of root tissue samples obtained by reverse transcription was used as a template, and PowerUp^TM^ SYBR Green Master mix reagent (Thermo Fisher, Shanghai, China) was used as a template. The target gene qRT-PCR detection was carried out on using platform of the ABI ViiA™ 7 Real-time PCR system (ABI, Carlsbad, CA, USA). Each gene was biologically replicated 3 times, with *Tubulin* as the internal reference gene, and the relative expression was calculated by the 2^−ΔΔCt^ method [35].

## 3. Results

### 3.1. Transcriptional Sequencing Quality Analysis of T. ramosissima Roots under NaCl Stress with Exogenous Potassium

15 high-quality Clean Data bases were obtained by IlluminaHiSeq^TM^4000 (Illumina, Inc., San Diego, CA, USA) transcriptome sequencing (5524761130—6617359533bp). Moreover, the Q20 reached more than 97%, the Q30 reached more than 92%, and the GC content reached more than 44.5%, indicating that the quality of transcriptome sequencing is reliable and in line with further bioinformatics analyses (Supplementary Table S1).

### 3.2. Mining and Expression Level Analysis of K^+^ Channel and Transporter-Related Genes

**Figure 1:**
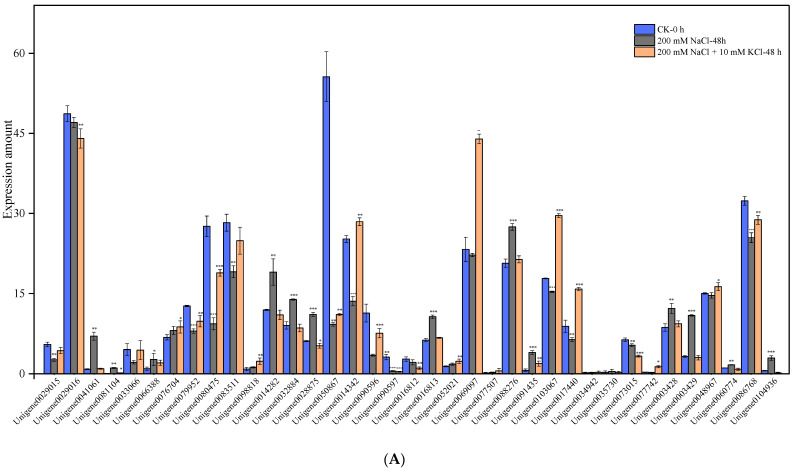

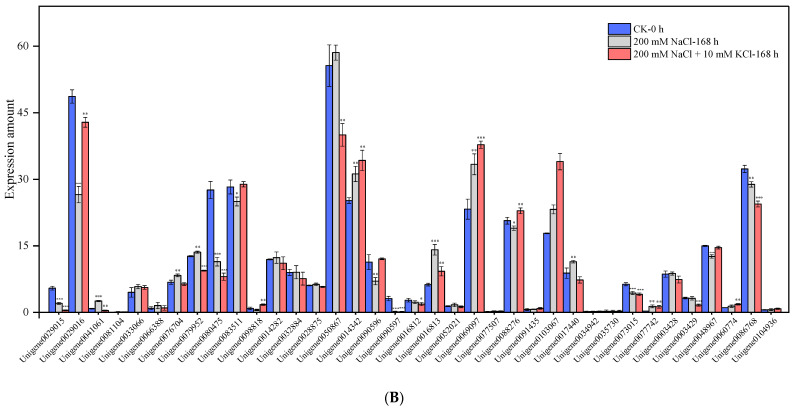
Changes in the expression of K^+^ channel and transporter-related genes in the roots of *T*. *ramosissima* with exogenous potassium application under NaCl stress (changes in expression levels of 37 K^+^ channel and transporter-related genes. Note: (**A**) represents the changes in the expression levels of 37 expressed genes in *T*. *ramosissima* under NaCl stress for 48 h; (**B**) represents the changes in the expression levels of 37 expressed genes in *T*. *ramosissima* under NaCl stress for 168 h; 0.01 < *p* < 0.05 is marked as *; 0.001 < *p* < 0.01 is marked as **; *p* ≤ 0.001 is marked as ***).

According to the transcriptional data of *T*. *ramosissima* roots treated with exogenous potassium for 0 h, 48 h and 168 h under NaCl stress, 37 related genes were found in Class 2 K^+^ channels and Class 3 K^+^ transporters (Table 2). The results showed (Figure 1) that the expression levels of 5 genes, including *Unigene0029015*, *Unigene0029016*, *Unigene0090597*, *Unigene0073015* and *Unigene0048967,* showed a decreasing trend at 48 h and 168 h when treated with 200 mM NaCl. The expression levels of 17 genes, including *Unigene0041061*, *Unigene0081104*, *Unigene0066388*, *Unigene0098818*, *Unigene0014282*, *Unigene0032884*, *Unigene0028875*, *Unigene0052021*, *Unigene0077507*, *Unigene0088276*, *Unigene0091435*, *Unigene0034942*, *Unigene0035730*, *Unigene0003428*, *Unigene0003429*, *Unigene0060774* and *Unigene0104936,* first increased and then decreased at 48 h and 168 h when treated with 200 mM NaCl. The expression levels of *Unigene0076704* and *Unigene0016813* showed a rising trend at 48 h and 168 h when treated with 200 mM NaCl. The expression levels of 13 genes, including *Unigene0033066*, *Unigene0079952, Unigene0080475, Unigene0083511, Unigene0050867, Unigene0014342, Unigene0090596, Unigene0016812, Unigene0069097, Unigene0103067, Unigene0017440, Unigene0077742* and *Unigene0086768* first decreased and then increased at 48 h and 168 h when treated with 200 mM NaCl. The expression levels of 7 genes, including *Unigene0029015*, *Unigene0029016*, *Unigene0079952*, *Unigene0014282*, *Unigene0032884*, *Unigene0028875* and *Unigene0086768* showed a decreasing trend at 48 h and 168 h when treated with 200 mM NaCl + 10 mM KCl. The expression levels of 16 genes, including *Unigene0041061*, *Unigene0081104*, *Unigene0066388*, *Unigene0076704*, *Unigene0080475*, *Unigene0098818*, *Unigene0090597*, *Unigene0052021*, *Unigene0069097*, *Unigene0077507*, *Unigene0091435*, *Unigene0017440*, *Unigene0077742*, *Unigene0003428*, *Unigene0003429* and *Unigene0048967* first increased and then decreased at 48 h and 168 h after 200 mM NaCl + 10 mM KCl treatment. The expression levels of 4 genes including, *Unigene0014342*, *Unigene0016813*, *Unigene0088276* and *Unigene0103067,* showed a rising trend at 48 h and 168 h when treated with 200 mM NaCl + 10 mM KCl. Ten expressed genes, including *Unigene0033066*, *Unigene0083511*, *Unigene0050867*, *Unigene0090596*, *Unigene0016812*, *Unigene0034942*, *Unigene0035730*, *Unigene0073015*, *Unigene0060774* and *Unigene0104936* first decreased and then increased at 48 h and 168 h when treated with 200 mM NaCl + 10 mM KCl. Notably, the expression levels of 3 genes, including *Unigene0014342*, *Unigene0088276* and *Unigene0103067,* first decreased and then increased at 48 h and 168 h when treated with 200 mM NaCl, but 200 mM NaCl + 10 mM KCl treatment for 48 h and 168 h led to a rising trend. This showed that they had an improved K^+^ uptake and K^+^/Na^+^ ratio, and the salt tolerance of *T*. *ramosissima* was enhanced. The expression levels of 4 expressed genes, including *Unigene0083511*, *Unigene0050867*, *Unigene0090596* and *Unigene0016812*, decreased with 200 mM NaCl and 200 mM NaCl + 10 mM KCl treatment for 48 h. However, the expression levels at 168 h showed an upward trend, indicating that they played a role at 168 h, and the addition of exogenous potassium could better help the roots of *T*. *ramosissima* to resist NaCl stress. The expression level of *Unigene0016813* showed an increasing trend at 48 h and 168 h after 200 mM NaCl and 200 mM NaCl + 10 mM KCl treatment, which played a role in improving the salt tolerance of *T*. *ramosissima.*

Finally, the obtained 37 expressed genes in K^+^ channels and transporters were annotated into GO and KEGG databases. It was found that 29 expressed genes in K^+^ channels and transporters were annotated in the GO database but not in KEGG data.

### 3.3. GO Enrichment and Expression Changes of DEGs in K^+^ Channels and Transporters

Only 1 expressed gene (*Unigene0081104*) was enriched to GO in Shaker channel; 4 expressed genes (*Unigene0033066, Unigene0066388, Unigene0080475* and *Unigene0098818*) were enriched to GO in TPK channel; 5 expressed genes (*Unigene0014282, Unigene0032884, Unigene0028875, Unigene0050867* and *Unigene0014342*) were enriched to GO in HAK/KUP/KT transporter; no expressed gene was enriched to GO in HKT transporter. 19 genes (*Unigene0016812, Unigene0016813, Unigene0052021, Unigene0069097, Unigene0077507, Unigene0088276, Unigene0091435, Unigene0103067, Unigene0017440, Unigene0034942, Unigene0035730, Unigene0073015, Unigene0077742, Unigene0003428, Unigene0003429, Unigene0048967, Unigene0060774, Unigene0086768* and *Unigene0104936*) were enriched to GO in CPAs transporters (Table 3). The results showed that the roots of *T*. *ramosissima* were treated with exogenous potassium for 48 h and 168 h under NaCl stress. A large number of genes in K^+^ channels and transporters are involved in the GO Biological Process, which can resist NaCl stress and Na^+^ poisoning by regulating the Biological Process.

**Figure 2:**
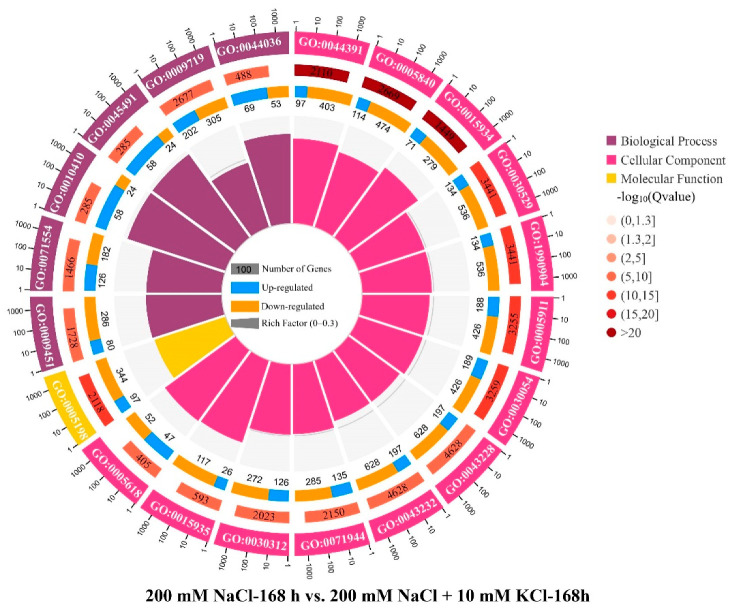

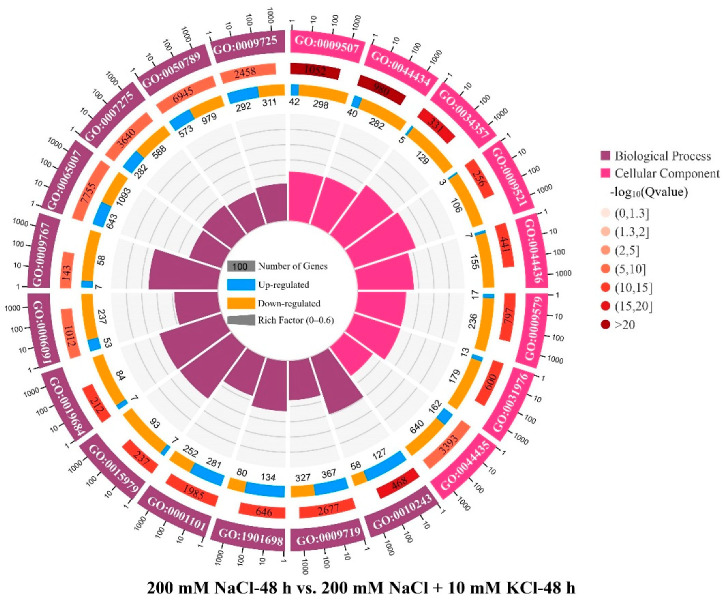
Top 20 GO enrichment (The first and outer circle: the top 20 GO terms are enriched, outside the circle is the scale of the number of genes. Different colors represent different ontologies. The second circle: the number of the GO term in the background gene and the Q value. The darker the color, the smaller the Q value. The longer the bars, the more genes they contain. The dark color represents the proportion of upregulated genes, and the light color represents the proportion of downregulated genes. The specific value is displayed below. The fourth and inner circle: the ratio of each GO term Rich Factor value (the number of differential genes in this GO term divided by all numbers), background grid lines; each grid represents 0.1).

According to the GO enrichment results (Figure 2), the top 20 GO enrichments in comparison groups 200 mM NaCl-48 h vs. 200 mM NaCl + 10 mM KCl-48 h and 200 mM NaCl-168 h vs. 200 mM NaCl + 10 mM KCl-168 h all have expressed genes that are involved in GO0009719. According to the enrichment results for 37 K^+^ channel and transporter-related genes to GO, in the 200 mM NaCl-48 h vs. 200 mM NaCl + 10 mM KCl-48 h comparison group, *Unigene0081104* and *Unigene0014342* participated in the GO Biological Process GO:0001101, *Unigene0052021* participated in GO:0065007 in GO Biological Process, and *Unigene0073015* participates in GO:0007275 in GO Molecular Function. Among them, the most expressed genes were involved in the GO Biological Process; however, no expressed genes were enriched to GO in the 200 mM NaCl-168 h vs. 200 mM NaCl + 10 mM KCl-168 h comparison group. Moreover, among the 37 K^+^ channel- and transporter-related genes that were screened, 8 DEGs, including *Unigene0029016* and *Unigene0081104* in the Shaker channel, *Unigene0090597* in the HKT transporter, and *Unigene0088276*, *Unigene0091435*, *Unigene0035730*, *Unigene0060774* and *Unigene0104936* in the CPAs transporter, were downregulated in the 200 mM NaCl-48 h vs. 200 mM NaCl + 10 mM KCl-48 h comparison group, but upregulated in the 200 mM NaCl-168 h vs. 200 mM NaCl + 10 mM KCl-168 h comparison group. 9 DEGs, including *Unigene0083511* and *Unigene0098818* in the TPK channel, *Unigene0014342* in the HAK/KUP/KT transporter, *Unigene0090596* in the HKT transporter, *Unigene0069097*, *Unigene0077507*, *Unigene0103067*, *Unigene0034942* and *Unigene0048967* in the CPAs transporter, were consistently upregulated in the 200 mM NaCl-48 h vs. 200 mM NaCl + 10 mM KCl-48 h and 200 mM NaCl-168 h vs. 200 mM NaCl + 10 mM KCl-168 h comparison group (Table 4). This indicates that these 17 DEGs played an important role in the response of *T*. *ramosissima* roots to exogenous potassium for 48 h and 168 h in response to NaCl stress. They improve the salt tolerance of *T*. *ramosissima* to reduce the Na^+^ poisoning of *T*. *ramosissima* and maintain growth.

### 3.4. OPLS-DA Model Analysis

*T*. *ramosissima* roots were treated with 200 mM NaCl and 200 mM NaCl + 10 mM KCl for 48 h and 168 h. A total of 1510 metabolites were identified in positive-ion mode, and 1124 metabolites were identified in negative-ion mode. In an OPLS-DA analysis of metabolomic data using Simca software, the results showed that differential metabolites existed in 200 mM NaCl and 200 mM NaCl + 10 mM KCl treatment for 48 h and 168 h in *T*. *ramosissima* roots, and the OPLS-DA model has excellent predictability and stability (Supplementary Figure S1).

### 3.5. Correlation Analysis of DEGs and Metabolites in K^+^ Channels and Transporters

According to the requirements of the absolute value of Person correlation coefficient |Corr| > 0.8, 37 DEGs and the metabolomic data of K^+^ channels and transporters in transcriptome data were screened and correlated in this study.

Firstly, Person correlation analysis was performed on the DEGs and metabolome data related to 17 important K^+^ channels and transporters involved in the application of exogenous potassium for 48 h and 168 h (Section 3.2 above) under NaCl stress in *T*. *ramosissima*. The results showed (Supplementary Figure S2) that, in the Shaker channel, *Unigene0029016* was correlated with 258 metabolites and *Unigene0029016* was significantly positively correlated with 4 metabolites and significantly negatively correlated with 254 metabolites; a correlation analysis of *Unigene0081104* and 52 metabolites found that *Unigene0081104* was significantly positively correlated with 47 metabolites, and significantly negatively correlated with 5 metabolites. In the TPK channel, the correlation analysis of *Unigene0083511* and 29 metabolites found that *Unigene0083511* was significantly positively correlated with 13 metabolites, and significantly negatively correlated with 16 metabolites; the correlation analysis of *Unigene0098818* and 78 metabolites found that *Unigene0098818* was significantly positively correlated with 27 metabolites and significantly negatively correlated with 51 metabolites. For the HAK/KUP/KT transporters, the correlation analysis of *Unigene0014342* and 32 metabolites found that *Unigene0014342* was significantly positively correlated with 9 metabolites and significantly negatively correlated with 23 metabolites. Among HKT transporters, the correlation analysis of *Unigene0090596* and 42 metabolites found that *Unigene0090596* was significantly positively correlated with 30 metabolites and significantly negatively correlated with 12 metabolites; correlation analysis of *Unigene0090597* and 155 metabolites found that *Unigene0090597* was significantly positively correlated with 147 metabolites, and significantly negatively correlated with 8 metabolites. Among CPA transporters, the correlation analysis of *Unigene0069097* and 109 metabolites found that *Unigene0069097* was significantly positively correlated with 61 metabolites, and significantly negatively correlated with 48 metabolites; correlation analysis of *Unigene0077507* and 12 metabolites found that *Unigene0077507* was significantly positively correlated with 12 metabolites; correlation analysis of *Unigene0088276* and 59 metabolites found that *Unigene0088276* was significantly positively correlated with 46 metabolites, and significantly correlated with 13 metabolites Negative correlation; correlation analysis of *Unigene0091435* and 37 metabolites found that *Unigene0091435* was significantly positively correlated with 28 metabolites, and significantly negatively correlated with 9 metabolites; correlation analysis of *Unigene0103067* and 29 metabolites found that *Unigene0103067* was significantly positively correlated with 17 metabolites, and significantly negatively correlated with 12 metabolites; correlation analysis of *Unigene0048967* and 206 metabolites found that *Unigene0048967* was significantly positively correlated with 8 metabolites, and significantly negatively correlated with 198 metabolites; correlation analysis of *Unigene0060774* and 68 metabolites found that *Unigene0060774* was significantly positively correlated with 41 metabolites, and significantly negatively correlated with 27 metabolites; correlation analysis of *Unigene0104936* and 80 metabolites found that *Unigene0104936* was significantly positively correlated with 71 metabolites, and significantly negatively correlated with 9 metabolites. Furthermore, *Unigene0034942* and *Unigene0035730* had no metabolites with their Person correlation coefficient absolute values |Corr| > 0.8.

Secondly, according to the requirement of the absolute value of Person correlation coefficient |Corr| > 0.8, the remaining 20 DEGs and metabolome data related to K^+^ channels and transporters were screened and correlated. The results showed that in the Shaker channel, *Unigene0029015* was correlated with 46 metabolites and correlation analysis found that *Unigene0029015* was significantly positively correlated with 24 metabolites and significantly negatively correlated with 22 metabolites; correlation analysis of *Unigene0041061* and 71 metabolites found that *Unigene0041061* was significantly positively correlated with 62 metabolites, and significantly negatively correlated with 9 metabolites (Supplementary Figure S3). In the TPK channel, the correlation analysis of *Unigene0033066* and 3 metabolites found that *Unigene0033066* was significantly positively correlated with 2 metabolites and significantly negatively correlated with 1 metabolite; correlation analysis of *Unigene0076704* and 27 metabolites found that *Unigene0076704* was significantly positively correlated with 7 metabolites, and significantly negatively correlated with 20 metabolites; correlation analysis of *Unigene0079952* and 85 metabolites found that *Unigene0079952* was significantly positively correlated with 77 metabolites, and significantly negatively correlated with 8 metabolites; correlation analysis of *Unigene0080475* and 108 metabolites found that *Unigene0080475* was significantly positively correlated with 85 metabolites, and significantly negatively correlated with 23 metabolites. Additionally, *Unigene0066388* has no metabolite and its Person correlation coefficient absolute value |Corr| > 0.8 (Supplementary Figure S4). Among the HAK/KUP/KT transporters, the correlation analysis of *Unigene0014282* and 34 metabolites found that *Unigene0014282* was significantly positively correlated with 33 metabolites and significantly negatively correlated with 1 metabolite; correlation analysis of *Unigene0032884* and 28 metabolites found that *Unigene0032884* was significantly positively correlated with 27 metabolites, and significantly negatively correlated with 1 metabolite; correlation analysis of *Unigene0028875* and 73 metabolites found that *Unigene0028875* was significantly positively correlated with 66 metabolites, and significantly negatively correlated with 7 metabolites; correlation analysis of *Unigene0050867* and 78 metabolites found that *Unigene0050867* was significantly positively correlated with 68 metabolites, and significantly negatively correlated with 10 metabolites (Supplementary Figure S5). Among CPAs transporters, the correlation analysis of *Unigene0016812* and 65 metabolites found that *Unigene0016812* was significantly positively correlated with 34 metabolites, and significantly negatively correlated with 31 metabolites; correlation analysis of *Unigene0016813* and 180 metabolites found that *Unigene0016813* was significantly positively correlated with 159 metabolites, and significantly negatively correlated with 21 metabolites; correlation analysis of *Unigene0052021* and 32 metabolites found that *Unigene0052021* was significantly positively correlated with 22 metabolites, and significantly negatively correlated with 11 metabolites; correlation analysis of *Unigene0017440* and 538 metabolites found that *Unigene0017440* was significantly positively correlated with 387 metabolites, and significantly negatively correlated with 151 metabolites; correlation analysis of *Unigene0073015* and 97 metabolites found that *Unigene0073015* was significantly positively correlated with 76 metabolites, and significantly negatively correlated with 21 metabolites; correlation analysis of *Unigene0077742* and 31 metabolites found that *Unigene0077742* was significantly positively correlated with 10 metabolites, and significantly negatively correlated with 21 metabolites; correlation analysis of *Unigene0003428* and 19 metabolites found that *Unigene0003428* was significantly positively correlated with 15 metabolites, and significantly negatively correlated with 4 metabolites; correlation analysis of *Unigene0003429* and 49 metabolites found that *Unigene0003429* was significantly positively correlated with 45 metabolites, and significantly negatively correlated with 4 metabolites; correlation analysis of *Unigene0086768* and 59 metabolites found that *Unigene0086768* was significantly positively correlated with 46 metabolites, and significantly negatively correlated with 13 metabolites (Supplementary Figure S6).

To summarize, except *Unigene0029016*, *Unigene0083511*, *Unigene0098818*, *Unigene0014342*, *Unigene0048967*, *Unigene0076704* and *Unigene0077742*, 7 DEGs in K^+^ channels and transporters negatively regulate their related metabolites. The remaining 30 DEGs and their metabolites were all positively correlated. The results showed that a high number of DEGs of K^+^ channels and transporters were found to positively regulated their related metabolites to resist salt stress in the roots of *T*. *ramosissima* with exogenous potassium application under NaCl stress for 48 h and 168 h, mitigating NaCl poisoning. However, the regulatory mechanism between these DEGs and metabolites needs further study.

### 3.6. Phylogenetic Tree Analysis of Key DEGs

**Figure 3:**
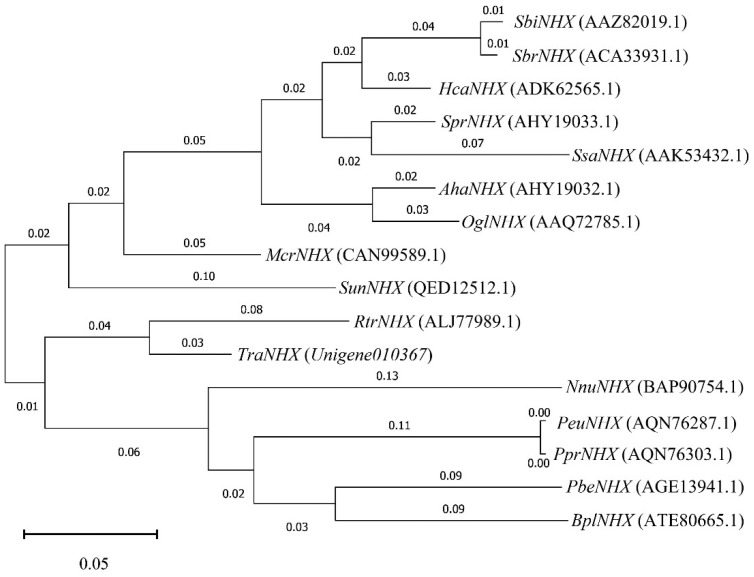
Phylogenetic tree analysis of *T*. *ramosissima* NHX and other species NHX. (Phylogenetic tree analysis of Unigene0103067 protein amino acid sequence and protein amino acid sequence of another 15 species of *T. ramosissima*).

The expression levels of key genes in K^+^ channels and transporters in response to exogenous potassium in *T*. *ramosissima* roots, and the correlation between DEGs and metabolites, were analyzed under NaCl stress. *Unigene0103067* was sensitive to the addition of exogenous potassium under NaCl stress in the CPAs transporter of *T*. *ramosissima.* Its expression level first decreased and then increased in 200 mM NaCl treatment for 48 h and 168 h, while its expression level showed an upward trend in 200 mM NaCl + 10 mM KCl treatment for 48 h and 168 h. The correlation analysis between *Unigene0103067* and its 29 related metabolites found that it was significantly positively correlated with 17 metabolites and negatively correlated with 12 metabolites. The results showed that *Unigene0103067* played an important role in *T*. *ramosissima* under NaCl stress by applying exogenous potassium for 48 h, which lasted until 168 h, and was dominated by positive regulation metabolites, thus enhancing the salt tolerance of *T*. *ramosissima* and playing an important role in reducing NaCl toxicity. Therefore, the protein amino acid sequence of *Unigene0103067* was selected for alignment on NCBI using BLAST. A total of 15 homologous gene species were selected (Table 5). Then, MEGA software was used to construct a phylogenetic tree by combining the amino acid sequence of *Unigene0103067* protein of *T*. *ramosissima* and the protein amino acid sequence of these 15 homologous gene species. The results showed that *Unigene0103067* was closely related to *Reaumuria trigyna* (Figure 3).

### 3.7. Quantitative Real-Time PCR (qRT-PCR) Validation of DEGs

**Figure 4:**
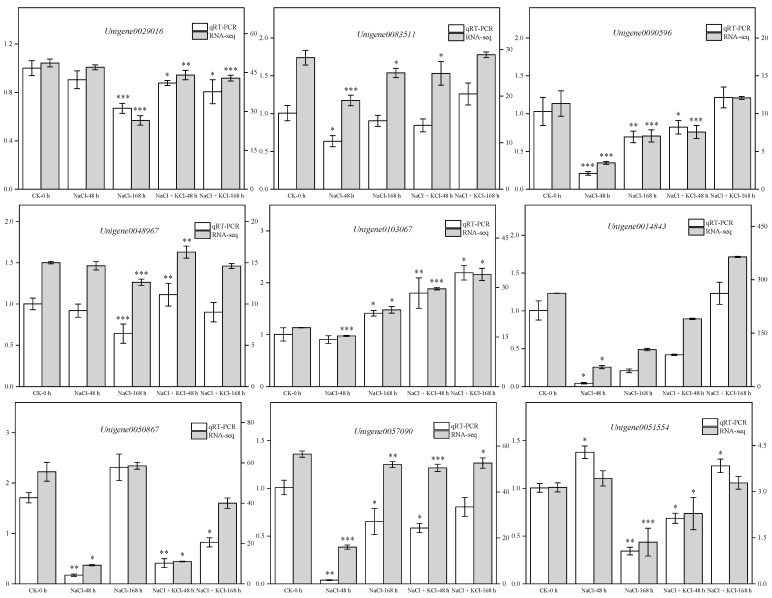
Validation of DEGs by qRT-PCR. (9 DEGs were randomly selected for qRT-PCR validation, and the error bars were obtained from multiple replicates of qRT-PCR. Note: NaCl means 200 mM NaCl treatment group, NaCl + KCl means 200 mM NaCl + 10 mM KCl treatment group). 0.01 < *p* < 0.05 is marked as *; 0.001 < *p* < 0.01 is marked as **; *p* ≤ 0.001 is marked as ***.

Referring to the method of Chen et al. [35] 9 DEGs (*Unigene0029016*, *Unigene083511*, *Unigene0090596*, *Unigene0048967*, *Unigene0103067*, *Unigene0014843*, *Unigene0057090*, *Unigene0050867* and *Unigene0051554*) were randomly selected for qRT-PCR verification. The results showed that the qRT-PCR verification results were entirely consistent with the expression trends of the transcriptome sequencing analysis results (Figure 4). This demonstrates that the transcriptome data obtained in this study are accurate and reliable. It can provide a theoretical basis for excavating the roots of *T*. *ramosissima* to promote K^+^ absorption, alleviate NaCl stress damage, and improve key salt tolerance genes.

## 4. Discussion

Under salt stress, a high amount of Na^+^ accumulates in plants; on the one hand, this inhibits the absorption of K^+^, and on the other hand, it competes with K^+^ for some enzymatic binding sites, affecting protein synthesis and ribosome function and resulting in Na^+^ toxicity [24,40,41,42]. It is generally believed that maintaining a high K^+^/Na^+^ ratio in the cytoplasm is one of the important measures by which plants adapt to salt stress [15,24,41].

K^+^ is a regular nutrient, and is involved in photosynthesis, the regulation of photosynthetic product transport, enzyme activation and Na^+^ uptake under salinity conditions. It can also enable plants to survive under stress conditions by regulating plant physiological processes, and plays an especially important role in improving tolerance to salt stress [42]. Halophytes have a strong competitive advantage in maintaining K^+^ stability under high Na^+^ stress [43]. The high absorption of K^+^ by the salt-tolerant genotype may be related to its selectivity for Na^+^. Salt-tolerant plants selectively absorb K^+^ and preferentially deliver K^+^ to the xylem over Na^+^, thereby accumulating more K^+^. Numerous studies have shown that adding potassium alleviates the adverse effects of Na^+^, improves the absorption of K^+^, and increases the K^+^/Na^+^ ratio under NaCl stress [44]. In this study, the expression levels of *Unigene0014342*, *Unigene0088276,* and *Unigene0103067* first decreased and then increased at 48 h and 168 h with 200 mM NaCl treatment. However, 200 mM NaCl + 10 mM KCl treatment for 48 h and 168 h showed a rising trend, and they all positively regulated their respective metabolites. This shows that they play an important role when adding exogenous potassium for 48 h, improving the salt tolerance of *T*. *ramosissima* and maintaining its growth, which is consistent with the previous research results [45,46]. In addition, the HKT transporter is activated in the plasma membrane [24], which plays an important role in K^+^ and Na^+^ transportation in higher plants. This can alleviate plant salt stress [47,48,49]. HKT transporters can take up K^+^ in culture environments with high NaCl or low K^+^ concentrations [43], have the ability to transport K^+^ through Na^+^ [50], and are involved in the recovery of sodium ions from the transpiration stream, preventing the further transportation of sodium ions to the leaves [51]. It can also increase root length and fresh weight and enhance salt tolerance [52]. In this study, *Unigene0090596* of the HKT transporter showed an upregulation trend in 200 mM NaCl-48 h vs. 200 mM NaCl + 10 mM KCl-48 h and 200 mM NaCl-168 h vs. 200 mM NaCl + 10 mM KCl-168 h expression levels at 48 h and 168 h after exogenous potassium was added. *Unigene0090597* was first downregulated at 200 mM NaCl-48 h vs. 200 mM NaCl + 10 mM KCl-48 h, but upregulated at 200 mM NaCl-168 h vs. 200 mM NaCl + 10 mM KCl-168 h expression levels. From the above, it can be seen that *Unigene0090596* and *Unigene0090597* in the HKT transporter are involved in NaCl stress and resist salt stress by positively regulating autocorrelated metabolites, especially 48 h and 168 h after the addition of exogenous potassium. *Unigene0090596* plays a role by resisting NaCl stress, similar to the results of previous studies [53]. Simultaneously, the root uptake of K^+^ in plants is partially dependent on the contribution of the HAK/KUP/KT transporter Cluster I [54], and Na^+^ has a weak competitive effect on the transport of K^+^, which is mediated by these transporters [55]. It has been reported that the overexpression of *OsHAK16* in rice increases the K^+^/Na^+^ ratio in the roots and shoots of transgenic rice, mainly due to the increased K^+^ concentration in the roots, but Na^+^ remained unchanged, which improved the salt tolerance of rice [56]. In this study, the expression of *Unigene0050867* in the HAK/KUP/KT transporter initially decreased and then increased in 200 mM NaCl and 200 mM NaCl + 10 mM KCl treatment for 48 h and 168 h. The results showed that *Unigene0050867* was activated within 48 h of treatment. The expression level of *Unigene0050867* was upregulated in the 200 mM NaCl-48 h vs. 200 mM NaCl + 10 mM KCl-48 h comparison group. Its related metabolites were positively regulated to rapidly respond to NaCl. Na^+^, K^+^/H^+^ antiporters (NHX) belong to the CPAs I subfamily of the monovalent cation/H+ antiporter CPAs gene family, which are widely present in plants. According to subcellular distribution, plant NHX gene family members can be divided into three types: plasma membrane NHX, vacuolar NHX and endosomal NHX. In this study, the related genes of vacuolar NHX that existed in the roots of *T*. *ramosissima* with the application of exogenous potassium under NaCl stress were *Unigene0069097* and *Unigene0103067*, respectively. The expression level of *Unigene0103067* first decreased and then increased under 200 mM NaCl stress at 48 h and 168 h, while the expression level of *Unigene0103067* showed a continuously increasing trend at 48 h and 168 h under 200 mM NaCl + 10 mM KCl treatment. At the same time, *Unigene0103067* was upregulated in 200 mM NaCl-48 h vs. 200 mM NaCl 10 mM KCl-48 h and 200 mM NaCl-168 h vs. 200 mM NaCl 10 mM KCl-168 h comparison groups, indicating that the addition of K^+^ increases *Unigene0103067* expression levels under NaCl stress. The results indicate that NHX in the roots of *T*. *ramosissima* is involved in cell expansion, pH adjustment, the protection of K^+^ homeostasis and resistance to salt stress. It can discharge the excess absorbed Na^+^ in the cell to the outside or regionalize Na^+^ in the vacuole to control the damage in Na^+^ accumulation to the membrane system, thus reducing salt poisoning, which is similar to the results of previous studies [57,58,59,60]. Therefore, under NaCl stress, *Unigene0103067* responded to the application of exogenous K^+^, increased the absorption of K^+^ by *T*. *ramosissima*, and alleviated the toxic effect of NaCl. The expression level of *Unigene0016813* shows a continuously increasing trend under the treatment of 200 mM NaCl and 200 mM NaCl + 10 mM KCl at 48 h and 168 h. Related metabolites were positively regulated; however, the mechanism of action needs to be further studied or justified.

## 5. Conclusions

This study utilizes metabolomic and transcriptomic analyses of 37 DEGs of K^+^ channel and transporter genes and their metabolites in response to NaCl stress and 30 DEGs to positively regulate their related metabolites, which were excavated in *T*. *ramosissima.* Under NaCl stress, 17 DEGs persisted in response to exogenous K^+^ application. *Unigene0103067* belongs to Vacuolar NHX, and its metabolites are predominant in positive regulation and play an important role in responding to exogenous K^+^, promoting K^+^ absorption and alleviating the toxic effect of NaCl. The application of exogenous potassium under NaCl stress helps *T*. *ramosissima* overcome oxidative damage to cells, improves the absorption of K^+^ by the roots, and relieves the toxicity of NaCl. Still, it cannot completely eliminate Na^+^ toxicity.

## Figures and Tables

**Table 1:** The sequences of specific primers.

ID	Primer Name	Primer Sequence (5′-3′)
1	*Unigene0029016*	F:CTCGCTGTTTGGTGTGATGT
		R:CCGCCGTCTTCAACCACAAC
2	*Unigene0083511*	F:GGAATCTGGCAAAATGGGTG
		R:GTCCTTCTCCGATACTTTCC
3	*Unigene0090596*	F:CTGCGAAAGAAGATTGAAAC
		R:AGAGTTTCCACGCTTTTCCT
4	*Unigene0048967*	F:TAAGTCGTGCCTCCAATCTC
		R:CTCAATCTGTGTGCCGCTTT
5	*Unigene0103067*	F:GTTACTTTCAGGCAGCAGAT
		R:GTTATCATTATCGCATTCCC
6	*Unigene0014843*	F:GATGGGGATGTTCACTTCTG
		R:CACCCTGATTCCCCGTCTTA
7	*Unigene0057090*	F:GGTAGATTCCCTCCTTGGTG
		R:GTAACCGCCAAAGCCACTAT
8	*Unigene0050867*	F:TATTGAAGAGGTAGGCGGCG
		R:CACTTCGCTTTCGCCCATTA
9	*Unigene0051554*	F:ATCATCGGGGCTGTTTCTGC
		R:CTCAGCCACAGCACCCTCAA
10	*Tubulin*	F:GCTGAGATTACAACCGCTG
		R:CTGTTCGTTTGGTCTTGATT

**Table 2:** K^+^ channel- and transporter-related genes.

Gene ID	Description
Shaker	
*Unigene0029015*	Potassium channel AKT1-like
*Unigene0029016*	Predicted: potassium channel AKT1
*Unigene0041061*	Potassium channel AKT2/3 isoform X2
*Unigene0081104*	Predicted: potassium channel AKT1
TPK	
*Unigene0033066*	Predicted: two-pore potassium channel 1-like
*Unigene0066388*	Two pore potassium channel c
*Unigene0076704*	Two-pore potassium channel 5-like isoform X2
*Unigene0079952*	Two-pore potassium channel 3-like
*Unigene0080475*	Predicted: two-pore potassium channel 1 isoform X2
*Unigene0083511*	two-pore potassium channel 3
*Unigene0098818*	Predicted: two-pore potassium channel 1 isoform X3
HAK/KUP/KT	
*Unigene0014282*	Low affinity potassium transport system protein kup isoform 1
*Unigene0032884*	Potassium transport system protein kup
*Unigene0028875*	Potassium transporter
*Unigene0050867*	High affinity H^+^/K^+^ symporter
*Unigene0014342*	Predicted: probable potassium transporter 13
HKT	
*Unigene0090596*	Sodium transporter HKT1
*Unigene0090597*	Sodium transporter HKT1
CPAs	
*Unigene0016812*	Predicted: sodium/hydrogen exchanger 1
*Unigene0016813*	Predicted: sodium/hydrogen exchanger 1-like
*Unigene0052021*	Sodium/hydrogen exchanger 4
*Unigene0069097*	Vacuolar membrane Na^+^/H^+^ antiporter
*Unigene0077507*	Na^+^/H^+^ exchanger 3, partial
*Unigene0088276*	Sodium/hydrogen exchanger 6 like, partial
*Unigene0091435*	Sodium/hydrogen exchanger 2-like
*Unigene0103067*	Vacuolar membrane Na^+^/H^+^ antiporter
*Unigene0017440*	Predicted: cation/H^+^ antiporter 20
*Unigene0034942*	Cation/H^+^ antiporter 15-like isoform X1
*Unigene0035730*	Predicted: cation/H^+^ antiporter 2-like
*Unigene0073015*	Cation/H^+^ antiporter 15-like
*Unigene0077742*	Cation/H^+^ antiporter like
*Unigene0003428*	K^+^ efflux antiporter 4-like isoform X1
*Unigene0003429*	K^+^ efflux antiporter 4-like isoform X2
*Unigene0048967*	Predicted: K^+^ efflux antiporter 5
*Unigene0060774*	K^+^ efflux antiporter 3, chloroplastic isoform X3
*Unigene0086768*	Predicted: K^+^ efflux antiporter 2, chloroplastic
*Unigene0104936*	K^+^ efflux antiporter 5-like

**Table 3:** GO enrichment of K^+^ channel- and transporter-related genes.

Gene ID	GO Cellular Component	GO Molecular Function	GO Biological Process
Shaker			
*Unigene0029015*	-	-	-
*Unigene0029016*	-	-	-
*Unigene0041061*	-	-	-
*Unigene0081104*	GO:0031224; GO:0043231	GO:0000166; GO:0005249; GO:0005515	GO:0000041; GO:0000904; GO:0001101; GO:0006970; GO:0007015; GO:0009267; GO:0009933; GO:0010053; GO:0010119; GO:0015698; GO:0022610; GO:0045229; GO:0048588; GO:0071805
TPK			
*Unigene0033066*	GO:0005774; GO:0031224	GO:0005249; GO:0046872	GO:0005976; GO:0006875; GO:0006970; GO:0006996; GO:0009845; GO:0034220; GO:0050794; GO:0051259; GO:0070838
*Unigene0066388*	GO:0005774	GO:0005249	GO:0006811
*Unigene0076704*	-	-	-
*Unigene0079952*	-	-	-
*Unigene0080475*	GO:0005774; GO:0031224	GO:0005249; GO:0046872	GO:0006875; GO:0016043; GO:0034220; GO:0050789; GO:0070838
*Unigene0083511*	-	-	-
*Unigene0098818*	GO:0005774; GO:0031224	GO:0005249; GO:0046872	GO:0005976; GO:0006875; GO:0006970; GO:0006996; GO:0009845; GO:0034220; GO:0050794; GO:0051259; GO:0070838
HAK/KUP/KT			
*Unigene0014282*	GO:0016020	-	GO:0006807; GO:0044237; GO:0044238; GO:0044763; GO:0071704
*Unigene0032884*	GO:0043231	-	-
*Unigene0028875*	GO:0009536; GO:0031224	GO:0046873	GO:0030001; GO:0034220
*Unigene0050867*	GO:0031224	GO:0022820	GO:0030001; GO:0034220
*Unigene0014342*	GO:0016020	GO:0000166; GO:0005249; GO:0005515	GO:0000041; GO:0000904; GO:0001101; GO:0006970; GO:0007015; GO:0009267; GO:0009933; GO:0010053; GO:0010119; GO:0015698; GO:0022610; GO:0045229; GO:0048588; GO:0071805
HKT			
*Unigene0090596*	-	-	-
*Unigene0090597*	-	-	-
CPAs			
*Unigene0016812*	GO:0031224	GO:0005451	GO:0006814; GO:0006970; GO:0015992; GO:0044763; GO:0055065; GO:0055067
*Unigene0016813*	GO:0016020	GO:0005451; GO:0046873	GO:0006814; GO:0010119; GO:0055065; GO:0055067; GO:0071805
*Unigene0052021*	GO:0016020; GO:0043231; GO:0044444	GO:0015299; GO:0046873	GO:0015672; GO:0030001; GO:0065007; GO:0098662
*Unigene0069097*	GO:0005773; GO:0031090; GO:0031224	GO:0005451; GO:0005515; GO:0046873	GO:0006625; GO:0006814; GO:0006970; GO:0010119; GO:0015992; GO:0048193; GO:0048827; GO:0055065; GO:0055067
*Unigene0077507*	GO:0016020	GO:0005451; GO:0046873	GO:0006814; GO:0044763; GO:0055065; GO:0055067
*Unigene0088276*	GO:0031224	GO:0005451	GO:0006814; GO:0015992; GO:0044763; GO:0055067
*Unigene0091435*	GO:0031090; GO:0031224	GO:0005451	GO:0006814; GO:0006970; GO:0015992; GO:0044763; GO:0055065; GO:0055067
*Unigene0103067*	GO:0005773; GO:0031090; GO:0031224	GO:0005451; GO:0005515; GO:0046873	GO:0006625; GO:0006814; GO:0006970; GO:0010119; GO:0015992; GO:0048193; GO:0048827; GO:0055065; GO:0055067
*Unigene0017440*	GO:0009536; GO:0031224	GO:0005451	GO:0006605; GO:0006814; GO:0006875; GO:0015992; GO:0055067
*Unigene0034942*	-	-	GO:0006812; GO:0009987
*Unigene0035730*	GO:0031224	GO:0005451	GO:0000904; GO:0006814; GO:0009664; GO:0009814; GO:0015992; GO:0033554
*Unigene0073015*	-	GO:0015297 GO:0007275	GO:0015672; GO:0044763
*Unigene0077742*	GO:0016020	GO:0015299	GO:0015672; GO:0016043; GO:0030001; GO:0044763; GO:0055067
*Unigene0003428*	GO:0031224	GO:0005451; GO:0046873; GO:0046914	GO:0015992; GO:0030001; GO:0044763
*Unigene0003429*	GO:0031224	GO:0005451; GO:0046873; GO:0046914	GO:0015992; GO:0030001; GO:0044763
*Unigene0048967*	GO:0031224	GO:0005451; GO:0046873	GO:0015992; GO:0030001; GO:0044763
*Unigene0060774*	GO:0031224; GO:0042170	GO:0005451; GO:0046873	GO:0006468; GO:0015992; GO:0030001; GO:0043269; GO:0044763
*Unigene0086768*	GO:0009526; GO:0031224	GO:0005451; GO:0046873	GO:0015992; GO:0030001; GO:0044763
*Unigene0104936*	GO:0031224	GO:0005451; GO:0046873	GO:0030001

**Table 4:** Changes in the expression of K^+^ channel- and transporter-related genes in the roots of *T*. *ramosissima* by exogenous potassium application under NaCl stress.

Gene ID	Log_2_Fold Change	
	200 mM NaCl-48 h vs. 200 mM NaCl + 10 mM KCl-48 h	200 mM NaCl-168 h vs. 200 mM NaCl + 10 mM KCl-168 h
Shaker		
*Unigene0029015*	0.72	−2.13
*Unigene0029016*	−0.10	0.69
*Unigene0041061*	−2.90	−2.56
*Unigene0081104*	−2.73	0.19
TPK		
*Unigene0033066*	1.04	−0.05
*Unigene0066388*	−0.38	−0.59
*Unigene0076704*	0.12	−0.38
*Unigene0079952*	0.30	−0.53
*Unigene0080475*	1.02	−0.51
*Unigene0083511*	0.38	0.21
*Unigene0098818*	0.96	1.68
HAK/KUP/KT		
*Unigene0014282*	−0.79	−0.16
*Unigene0032884*	−0.70	−0.25
*Unigene0028875*	−1.09	−0.13
*Unigene0050867*	0.26	−0.55
*Unigene0014342*	1.07	0.13
HKT		
*Unigene0090596*	1.12	0.78
*Unigene0090597*	−0.41	0.07
CPAs		
*Unigene0016812*	−1.03	−0.30
*Unigene0016813*	−0.67	−0.61
*Unigene0052021*	0.39	−0.43
*Unigene0069097*	0.98	0.18
*Unigene0077507*	1.28	0.08
*Unigene0088276*	−0.36	0.27
*Unigene0091435*	−1.07	0.40
*Unigene0103067*	0.95	0.55
*Unigene0017440*	1.31	−0.64
*Unigene0034942*	0.24	0.06
*Unigene0035730*	−0.74	0.10
*Unigene0073015*	−0.68	−0.10
*Unigene0077742*	2.47	−0.11
*Unigene0003428*	−0.39	−0.24
*Unigene0003429*	−1.86	−0.95
*Unigene0048967*	0.15	0.21
*Unigene0060774*	−0.99	0.39
*Unigene0086768*	0.18	−0.24
*Unigene0104936*	−4.12	0.38

**Table 5:** Information sheet for 15 species.

Family	Species	Description	Gene	Protein ID	CDS (bp)	ORF Length (aa)
Tamaricaceae	*Reaumuria trigyna*	vacuolar membrane Na^+^/H^+^ antiporter	*RtrNHX*	ALJ77989.1	1662	553
Amaranthaceae	*Suaeda pruinosa*	Na^+^/H^+^ antiporter	*SprNHX*	AHY19033.1	1662	553
Amaranthaceae	*Atriplex halimus*	Na^+^/H^+^ antiporter	*AhaNHX*	AHY19032.1	1668	555
Amaranthaceae	*Salicornia bigelovii*	Na^+^/H^+^ antiporter	*SbiNHX*	AAZ82019.1	1683	560
Amaranthaceae	*Halostachys caspica*	Na^+^/H^+^ antiporter	*HcaNHX*	ADK62565.1	1656	551
Amaranthaceae	*Oxybasis glauca*	Na^+^/H^+^ antiporter	*OglNHX*	AAQ72785.1	1656	551
Amaranthaceae	*Salicornia brachiata*	Na^+^/H^+^ antiporter	*SbrNHX*	ACA33931.1	1683	560
Amaranthaceae	*Suaeda salsa*	Na^+^/H^+^ antiporter	*SsaNHX*	AAK53432.1	1671	556
Salicaceae	*Populus pruinosa*	Na^+^/H^+^ antiporter	*PprNHX*	AQN76303.1	1635	544
Betulaceae	*Betula platyphylla*	Na^+^/H^+^ antiporter	*BplNHX*	ATE80665.1	1626	541
Aizoaceae	*Mesembryanthemum crystallinum*	vacuolar Na^+^/H^+^ antiporter	*McrNHX*	CAN99589.1	1671	556
Cactaceae	*Selenicereus undatus*	Na^+^/H^+^ antiporter	*SunNHX*	QED12512.1	1662	553
Salicaceae	*Populus euphratica*	Na^+^/H^+^ antiporter	*PeuNHX*	AQN76287.1	1635	544
Rosaceae	*Pyrus betulifolia*	vacuolar Na^+^/H^+^ antiporter	*PbeNHX*	AGE13941.1	1629	542
Nelumbonaceae	*Nelumbo nucifera*	vacuolar Na^+^/H^+^ antiporter	*NnuNHX*	BAP90754.1	1617	538

## Data Availability

Not applicable.

## Supplementary Materials

The following supporting information can be downloaded at: https://www.mdpi.com/article/10.3390/genes13081313/s1, Supplementary Table S1: Filtered reads quality statistics. Supplementary Figure S1: OPLS-DA and model validation of the roots’ metabolites of *T*. *ramosissima* under 2 treatments. Supplementary Figure S2: Heatmap of correlations between major DEGs and metabolites in K^+^ channels and transporters. Supplementary Figure S3: Heatmap of correlations between major DGEs and metabolites in the Shaker channel. Supplementary Figure S4: Heatmap of correlations between major DGEs and metabolites in the TPK channel. Supplementary Figure S5: Heatmap of correlations between major DGEs and metabolites in the HAK/KUP/KT transporter family. Supplementary Figure S6: Heatmap of correlations between major DGEs and metabolites in the CPAs transporter family.
