# Supplementary material for: Transcriptome and Metabonomic Analysis of Tamarix ramosissima Potassium (K+) Channels and Transporters in Response to NaCl Stress
Source: Genes (Basel). 2022 Jul 23;13(8):1313. doi: 10.3390/genes13081313 (PMC9394374; doi:10.3390/genes13081313)
Supplement: Supplementary file 1 [file genes-13-01313-s001.zip › Supplementary Figure S1.pdf]

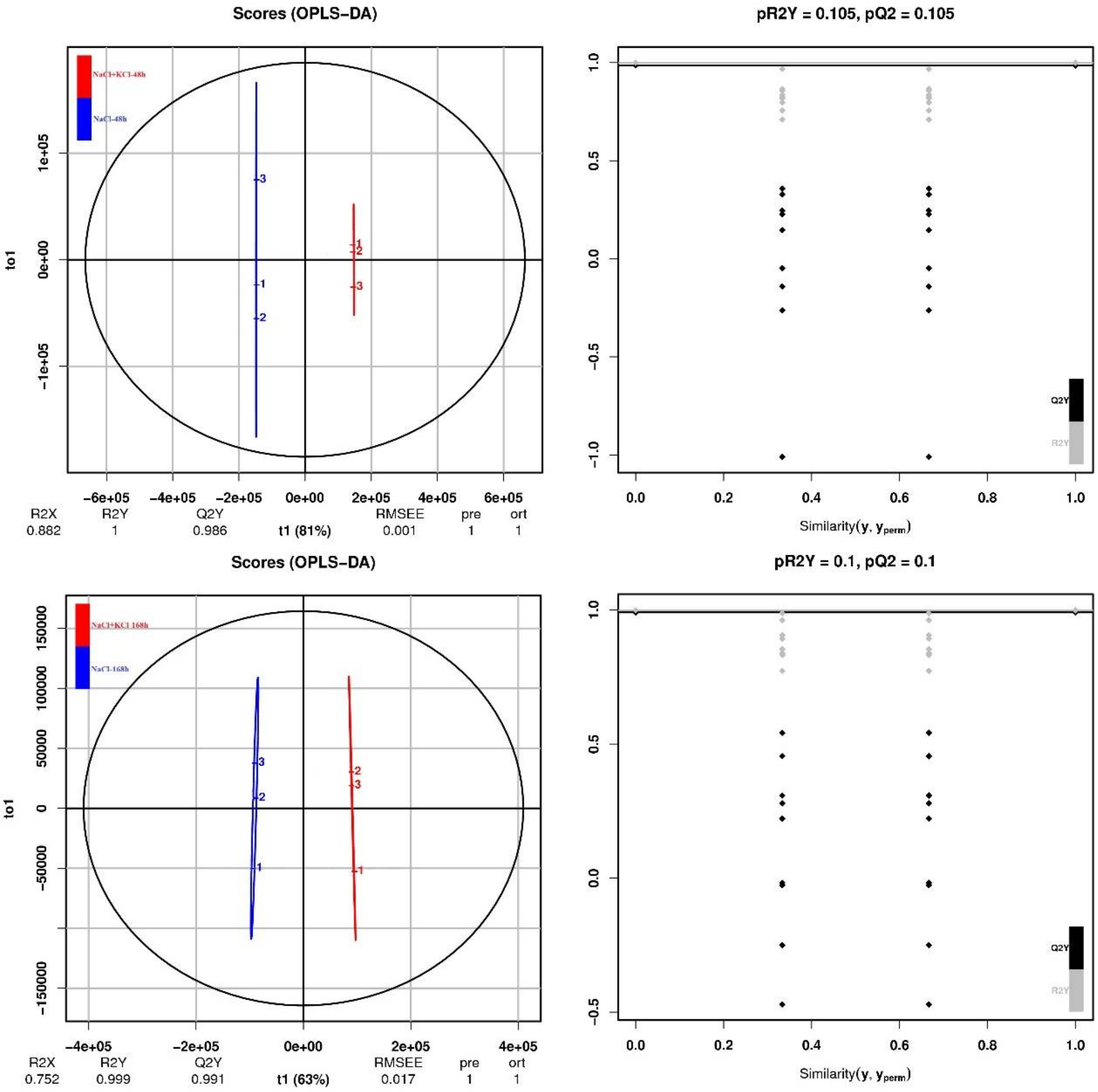

Positive ion mode

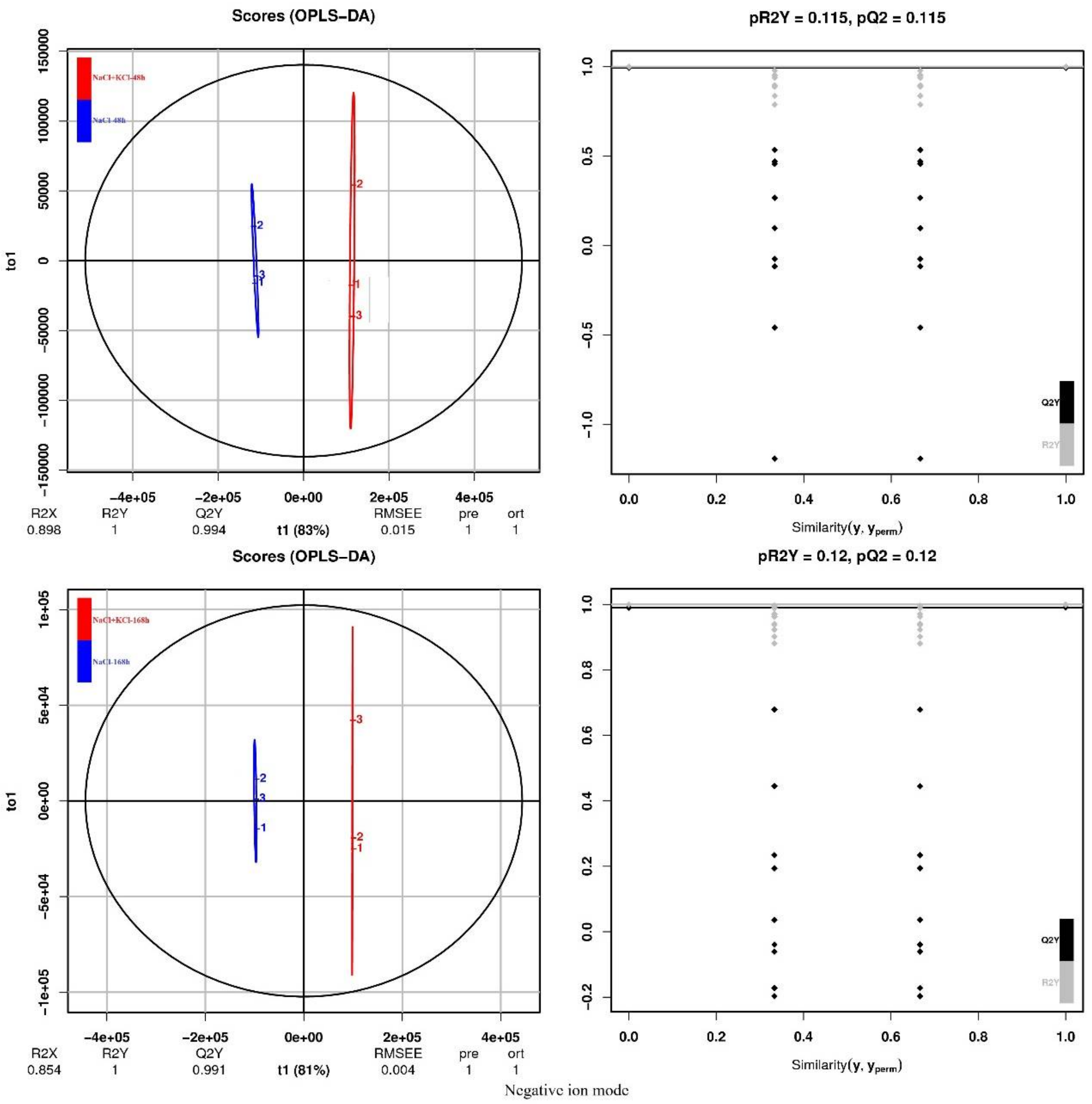

Supplementary Figure S1. OPLS-DA and model validation of the roots' metabolites of *T. ramosissima* under 2 treatments

(OPLS-DA analysis and model validation in positive and negative modes of *T. ramosissima* roots under NaCl stress at 0h, 48h and 168h after application of exogenous potassium. Note: 200 mM NaCl group; NaCl + KCl means 200 mM NaCl + 10 mM KCl group; Q2Y>0.5 indicates that the model has good predictive ability, and Q2Y>0.9 indicates that the model has an excellent predictive ability.)
