# Supplementary material for: Transcriptome and Metabonomic Analysis of Tamarix ramosissima Potassium (K+) Channels and Transporters in Response to NaCl Stress
Source: Genes (Basel). 2022 Jul 23;13(8):1313. doi: 10.3390/genes13081313 (PMC9394374; doi:10.3390/genes13081313)
Supplement: Supplementary file 1 [file genes-13-01313-s001.zip › Supplementary Figure S2.pdf]

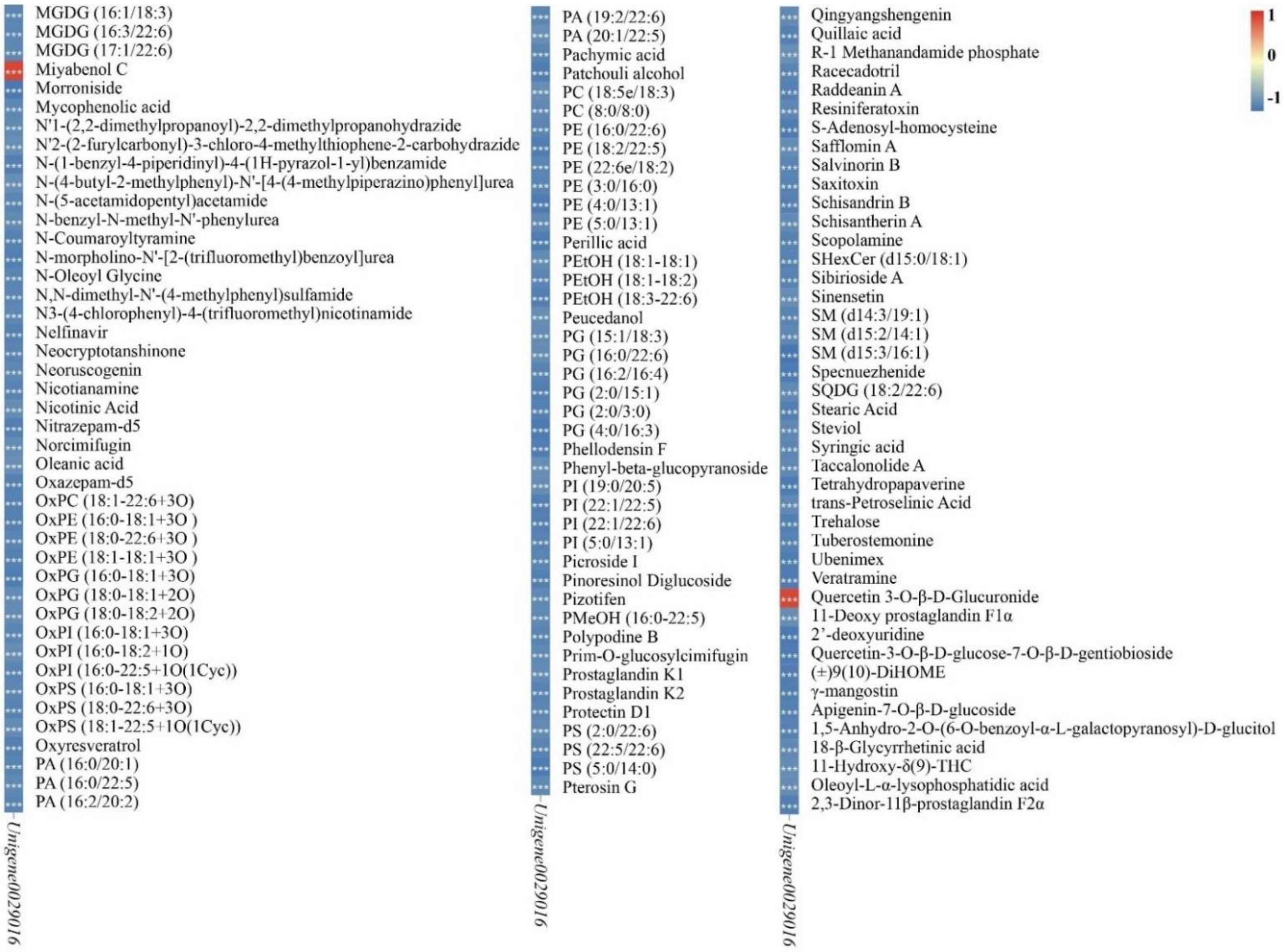
MGDG (16:1/18:3)
MGDG (16:3/22:6)
MGDG (17:1/22:6)
Miyabenol C
Morroniside
Mycophenolic acid
N'1-(2,2-dimethylpropanoyl)-2,2-dimethylpropanohydrazide
N'2-(2-furylcarbonyl)-3-chloro-4-methylthiophene-2-carbohydrazide
N-(1-benzyl-4-piperidinyl)-4-(1H-pyrazol-1-yl)benzamide
N-(4-butyl-2-methylphenyl)-N'-[4-(4-methylpiperazino)phenyl]urea
N-(5-acetamidopentyl)acetamide
N-benzyl-N-methyl-N'-phenylurea
N-Coumaroyltyramine
N-morpholino-N'-[2-(trifluoromethyl)benzoyl]urea
N-Oleoyl Glycine
N,N-dimethyl-N'-(4-methylphenyl)sulfamide
N3-(4-chlorophenyl)-4-(trifluoromethyl)nicotinamide
Nelfinavir
Neocryptotanshinone
Neoruscogenin
Nicotianamine
Nicotinic Acid
Nitrazepam-d5
Norcimifugin
Oleanic acid
Oxazepam-d5
OxPC (18:1-22:6+3O)
OxPE (16:0-18:1+3O)
OxPE (18:0-22:6+3O)
OxPE (18:1-18:1+3O)
OxPG (16:0-18:1+3O)
OxPG (18:0-18:1+2O)
OxPG (18:0-18:2+2O)
OxPI (16:0-18:1+3O)
OxPI (16:0-18:2+1O)
OxPI (16:0-22:5+1O(1Cyc))
OxPS (16:0-18:1+3O)
OxPS (18:0-22:6+3O)
OxPS (18:1-22:5+1O(1Cyc))
Oxyresveratrol
PA (16:0/20:1)
PA (16:0/22:5)
PA (16:2/20:2)
Unigene0029016
PA (19:2/22:6)
PA (20:1/22:5)
Pachymic acid
Patchouli alcohol
PC (18:5e/18:3)
PC (8:0/8:0)
PE (16:0/22:6)
PE (18:2/22:5)
PE (22:6e/18:2)
PE (3:0/16:0)
PE (4:0/13:1)
PE (5:0/13:1)
Perillic acid
PEtOH (18:1-18:1)
PEtOH (18:1-18:2)
PEtOH (18:3-22:6)
Peucedanol
PG (15:1/18:3)
PG (16:0/22:6)
PG (16:2/16:4)
PG (2:0/15:1)
PG (2:0/3:0)
PG (4:0/16:3)
Phellodensin F
Phenyl-beta-glucopyranoside
PI (19:0/20:5)
PI (22:1/22:5)
PI (22:1/22:6)
PI (5:0/13:1)
Picroside I
Pinoresinol Diglucoside
Pizotifen
PMeOH (16:0-22:5)
Polypodine B
Prim-O-glucosylcimifugin
Prostaglandin K1
Prostaglandin K2
Protectin D1
PS (2:0/22:6)
PS (22:5/22:6)
PS (5:0/14:0)
Pterosin G
Unigene0029016
Qingyangshengenin
Quillaic acid
R-1 Methanandamide phosphate
Racecadotril
Raddeanin A
Resiniferatoxin
S-Adenosyl-homocysteine
Safflomin A
Salvinorin B
Saxitoxin
Schisandrin B
Schisantherin A
Scopolamine
SHexCer (d15:0/18:1)
Sibirioside A
Sinensetin
SM (d14:3/19:1)
SM (d15:2/14:1)
SM (d15:3/16:1)
Specnuezhenide
SQDG (18:2/22:6)
Stearic Acid
Steviol
Syringic acid
Taccalonolide A
Tetrahydropapaverine
trans-Petroselinic Acid
Trehalose
Tuberostemonine
Ubenimex
Veratramine
Quercetin 3-O-β-D-Glucuronide
11-Deoxy prostaglandin F1α
2'-deoxyuridine
Quercetin-3-O-β-D-glucose-7-O-β-D-gentiobioside
(±)9(10)-DiHOME
γ-mangostin
Apigenin-7-O-β-D-glucoside
1,5-Anhydro-2-O-(6-O-benzoyl-α-L-galactopyranosyl)-D-glucitol
18-β-Glycyrrhetinic acid
11-Hydroxy-δ(9)-THC
Oleoyl-L-α-lysophosphatidic acid
2,3-Dinor-11β-prostaglandin F2α
Unigene0029016
1
0
-1

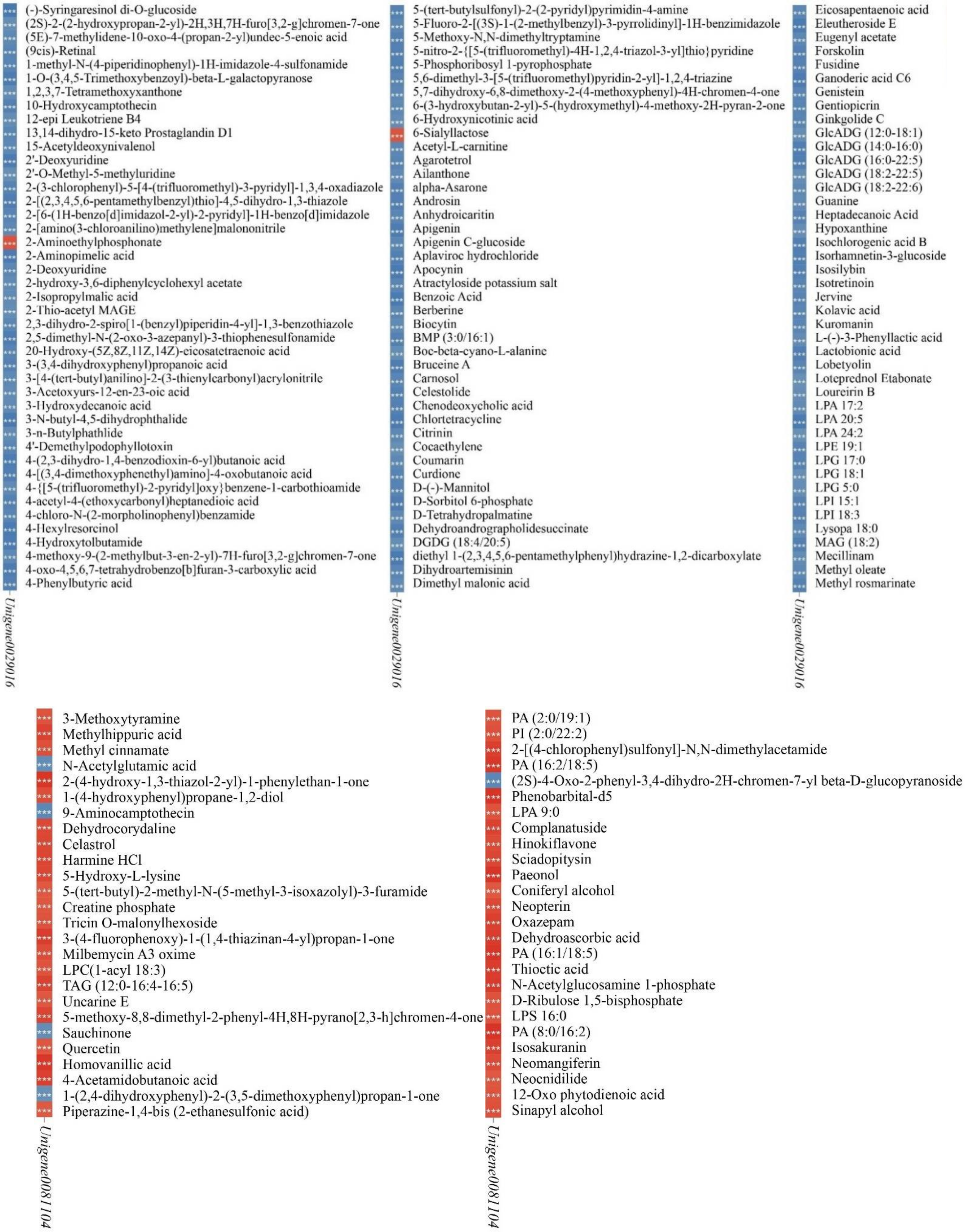
(-)-Syringaresinol di-O-glucoside
(2S)-2-(2-hydroxypropan-2-yl)-2H,3H,7H-furo[3,2-g]chromen-7-one
(5E)-7-methylidene-10-oxo-4-(propan-2-yl)undec-5-enoic acid
(9cis)-Retinal
1-methyl-N-(4-piperidinophenyl)-1H-imidazole-4-sulfonamide
1-O-(3,4,5-Trimethoxybenzoyl)-beta-L-galactopyranose
1,2,3,7-Tetramethoxyxanthone
10-Hydroxycamptothecin
12-epi Leukotriene B4
13,14-dihydro-15-keto Prostaglandin D1
15-Acetyldeoxynivalenol
2'-Deoxyuridine
2'-O-Methyl-5-methyluridine
2-(3-chlorophenyl)-5-[4-(trifluoromethyl)-3-pyridyl]-1,3,4-oxadiazole
2-[(2,3,4,5,6-pentamethylbenzyl)thio]-4,5-dihydro-1,3-thiazole
2-[6-(1H-benzo[d]imidazol-2-yl)-2-pyridyl]-1H-benzo[d]imidazole
2-[amino(3-chloroanilino)methylene]malononitrile
2-Aminoethylphosphonate
2-Aminopimelic acid
2-Deoxyuridine
2-hydroxy-3,6-diphenylcyclohexyl acetate
2-Isopropylmalic acid
2-Thio-acetyl MAGE
2,3-dihydro-2-spiro[1-(benzyl)piperidin-4-yl]-1,3-benzothiazole
2,5-dimethyl-N-(2-oxo-3-azepanyl)-3-thiophenesulfonamide
20-Hydroxy-(5Z,8Z,11Z,14Z)-eicosatetraenoic acid
3-(3,4-dihydroxyphenyl)propanoic acid
3-[4-(tert-butyl)anilino]-2-(3-thienylcarbonyl)acrylonitrile
3-Acetoxyurs-12-en-23-oic acid
3-Hydroxydecanoic acid
3-N-butyl-4,5-dihydrophthalide
3-n-Butylphathlide
4'-Demethylpodophyllotoxin
4-(2,3-dihydro-1,4-benzodioxin-6-yl)butanoic acid
4-[(3,4-dimethoxyphenethyl)amino]-4-oxobutanoic acid
4-{[5-(trifluoromethyl)-2-pyridyl]oxy}benzene-1-carbothioamide
4-acetyl-4-(ethoxycarbonyl)heptanedioic acid
4-chloro-N-(2-morpholinophenyl)benzamide
4-Hexylresorcinol
4-Hydroxytolbutamide
4-methoxy-9-(2-methylbut-3-en-2-yl)-7H-furo[3,2-g]chromen-7-one
4-oxo-4,5,6,7-tetrahydrobenzo[b]furan-3-carboxylic acid
4-Phenylbutyric acid
Unigene0029016
5-(tert-butylsulfonyl)-2-(2-pyridyl)pyrimidin-4-amine
5-Fluoro-2-[(3S)-1-(2-methylbenzyl)-3-pyrrolidinyl]-1H-benzimidazole
5-Methoxy-N,N-dimethyltryptamine
5-nitro-2-{[5-(trifluoromethyl)-4H-1,2,4-triazol-3-yl]thio}pyridine
5-Phosphoribosyl 1-pyrophosphate
5,6-dimethyl-3-[5-(trifluoromethyl)pyridin-2-yl]-1,2,4-triazine
5,7-dihydroxy-6,8-dimethoxy-2-(4-methoxyphenyl)-4H-chromen-4-one
6-(3-hydroxybutan-2-yl)-5-(hydroxymethyl)-4-methoxy-2H-pyran-2-one
6-Hydroxynicotinic acid
6-Sialyllactose
Acetyl-L-carnitine
Agarotetrol
Ailanthone
alpha-Asarone
Androsin
Anhydroicaritin
Apigenin
Apigenin C-glucoside
Aplaviroc hydrochloride
Apocynin
Atractyloside potassium salt
Benzoic Acid
Berberine
Biocytin
BMP (3:0/16:1)
Boc-beta-cyano-L-alanine
Bruceine A
Carnosol
Celestolide
Chenodeoxycholic acid
Chlortetracycline
Citrinin
Cocaethylene
Coumarin
Curdione
D-(-)-Mannitol
D-Sorbitol 6-phosphate
D-Tetrahydropalmatine
Dehydroandrographolidesuccinate
DGDG (18:4/20:5)
diethyl 1-(2,3,4,5,6-pentamethylphenyl)hydrazine-1,2-dicarboxylate
Dihydroartemisinin
Dimethyl malonic acid
Unigene0029016
Eicosapentaenoic acid
Eleutheroside E
Eugenyl acetate
Forskolin
Fusidine
Ganoderic acid C6
Genistein
Gentiopicrin
Ginkgolide C
GlcADG (12:0-18:1)
GlcADG (14:0-16:0)
GlcADG (16:0-22:5)
GlcADG (18:2-22:5)
GlcADG (18:2-22:6)
Guanine
Heptadecanoic Acid
Hypoxanthine
Isochlorogenic acid B
Isorhamnetin-3-glucoside
Isosilybin
Isotretinoin
Jervine
Kolavic acid
Kuromanin
L-(-)-3-Phenyllactic acid
Lactobionic acid
Lobetyolin
Loteprednol Etabonate
Loureirin B
LPA 17:2
LPA 20:5
LPA 24:2
LPE 19:1
LPG 17:0
LPG 18:1
LPG 5:0
LPI 15:1
LPI 18:3
Lysopa 18:0
MAG (18:2)
Mecillinam
Methyl oleate
Methyl rosmarinate
Unigene0029016
3-Methoxytyramine
Methylhippuric acid
Methyl cinnamate
N-Acetylglutamic acid
2-(4-hydroxy-1,3-thiazol-2-yl)-1-phenylethan-1-one
1-(4-hydroxyphenyl)propane-1,2-diol
9-Aminocamptothecin
Dehydrocorydaline
Celastrol
Harmine HCl
5-Hydroxy-L-lysine
5-(tert-butyl)-2-methyl-N-(5-methyl-3-isoxazolyl)-3-furamide
Creatine phosphate
Tricin O-malonylhexoside
3-(4-fluorophenoxy)-1-(1,4-thiazinan-4-yl)propan-1-one
Milbemycin A3 oxime
LPC(1-acyl 18:3)
TAG (12:0-16:4-16:5)
Uncarine E
5-methoxy-8,8-dimethyl-2-phenyl-4H,8H-pyrano[2,3-h]chromen-4-one
Sauchinone
Quercetin
Homovanillic acid
4-Acetamidobutanoic acid
1-(2,4-dihydroxyphenyl)-2-(3,5-dimethoxyphenyl)propan-1-one
Piperazine-1,4-bis (2-ethanesulfonic acid)
Unigene0081104
PA (2:0/19:1)
PI (2:0/22:2)
2-[(4-chlorophenyl)sulfonyl]-N,N-dimethylacetamide
PA (16:2/18:5)
(2S)-4-Oxo-2-phenyl-3,4-dihydro-2H-chromen-7-yl beta-D-glucopyranoside
Phenobarbital-d5
LPA 9:0
Complanatuside
Hinokiflavone
Sciadopitysin
Paeonol
Coniferyl alcohol
Neopterin
Oxazepam
Dehydroascorbic acid
PA (16:1/18:5)
Thioctic acid
N-Acetylglucosamine 1-phosphate
D-Ribulose 1,5-bisphosphate
LPS 16:0
PA (8:0/16:2)
Isosakuranin
Neomangiferin
Neocnidilide
12-Oxo phytodienoic acid
Sinapyl alcohol
Unigene0081104

A

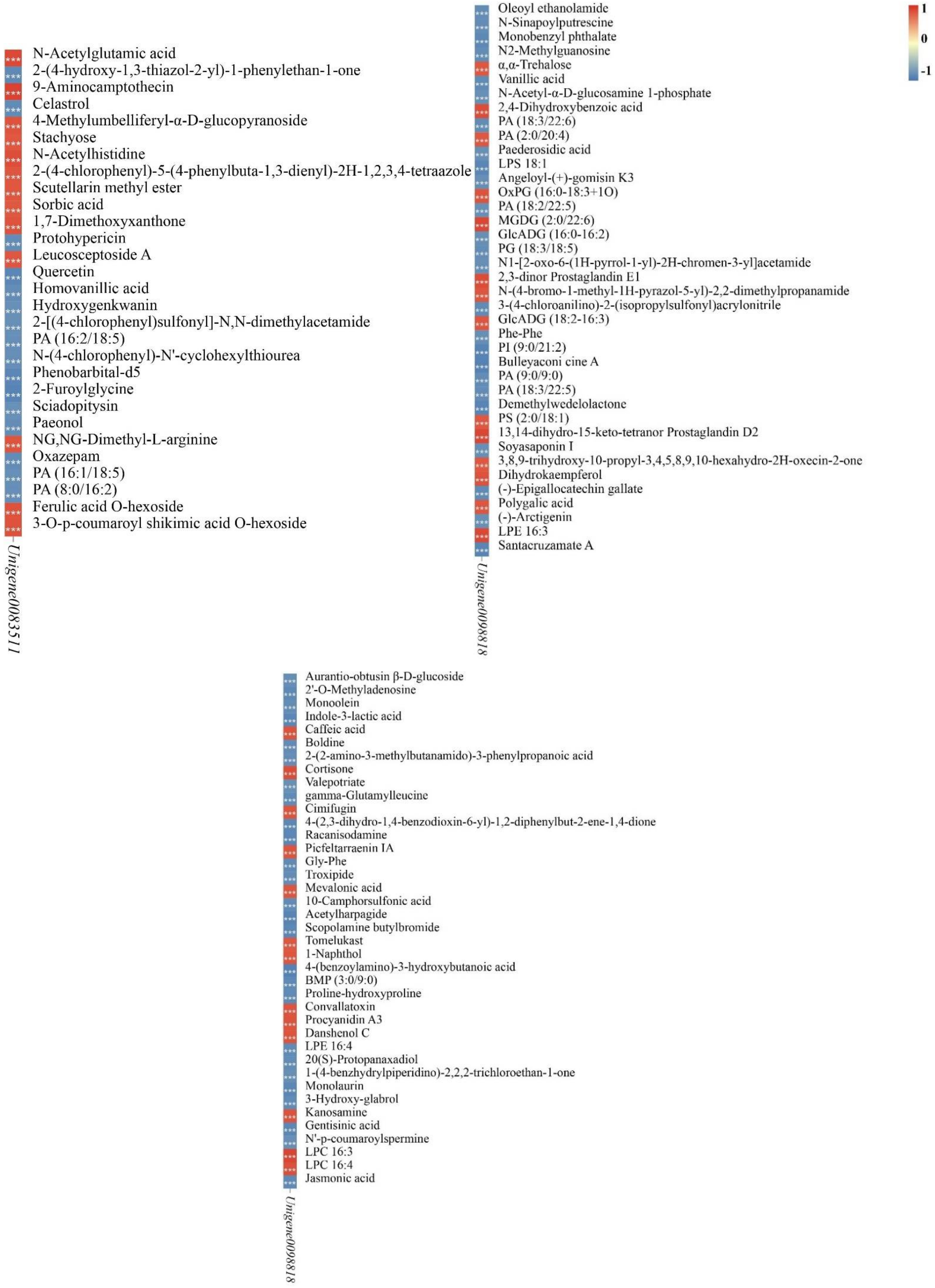

N-Acetylglutamic acid
2-(4-hydroxy-1,3-thiazol-2-yl)-1-phenylethan-1-one
9-Aminocamptothecin
Celastrol
4-Methylumbelliferyl-α-D-glucopyranoside
Stachyose
N-Acetylhistidine
2-(4-chlorophenyl)-5-(4-phenylbuta-1,3-dienyl)-2H-1,2,3,4-tetraazole
Scutellarin methyl ester
Sorbic acid
1,7-Dimethoxyxanthone
Protohypericin
Leucosceptoside A
Quercetin
Homovanillic acid
Hydroxygenkwanin
2-[(4-chlorophenyl)sulfonyl]-N,N-dimethylacetamide
PA (16:2/18:5)
N-(4-chlorophenyl)-N'-cyclohexylthiourea
Phenobarbital-d5
2-Furoylglycine
Sciadopitysin
Paeonol
NG,NG-Dimethyl-L-arginine
Oxazepam
PA (16:1/18:5)
PA (8:0/16:2)
Ferulic acid O-hexoside
3-O-p-coumaroyl shikimic acid O-hexoside
Unigene0083511
Oleoyl ethanolamide
N-Sinapoylputrescine
Monobenzyl phthalate
N2-Methylguanosine
α,α-Trehalose
Vanillic acid
N-Acetyl-α-D-glucosamine 1-phosphate
2,4-Dihydroxybenzoic acid
PA (18:3/22:6)
PA (2:0/20:4)
Paederosidic acid
LPS 18:1
Angeloyl-(+)-gomisin K3
OxPG (16:0-18:3+1O)
PA (18:2/22:5)
MGDG (2:0/22:6)
GlcADG (16:0-16:2)
PG (18:3/18:5)
N1-[2-oxo-6-(1H-pyrrol-1-yl)-2H-chromen-3-yl]acetamide
2,3-dinor Prostaglandin E1
N-(4-bromo-1-methyl-1H-pyrazol-5-yl)-2,2-dimethylpropanamide
3-(4-chloroanilino)-2-(isopropylsulfonyl)acrylonitrile
GlcADG (18:2-16:3)
Phe-Phe
PI (9:0/21:2)
Bulleyaconi cine A
PA (9:0/9:0)
PA (18:3/22:5)
Demethylwedelolactone
PS (2:0/18:1)
13,14-dihydro-15-keto-tetranor Prostaglandin D2
Soyasaponin I
3,8,9-trihydroxy-10-propyl-3,4,5,8,9,10-hexahydro-2H-oxecin-2-one
Dihydrokaempferol
(-)-Epigallocatechin gallate
Polygalic acid
(-)-Arctigenin
LPE 16:3
Santacruzamate A
Unigene0098818
1
0
-1
Aurantio-obtusin β-D-glucoside
2'-O-Methyladenosine
Monoolein
Indole-3-lactic acid
Caffeic acid
Boldine
2-(2-amino-3-methylbutanamido)-3-phenylpropanoic acid
Cortisone
Valepotriate
gamma-Glutamylleucine
Cimifugin
4-(2,3-dihydro-1,4-benzodioxin-6-yl)-1,2-diphenylbut-2-ene-1,4-dione
Racanisodamine
Picfeltarraenin IA
Gly-Phe
Troxipide
Mevalonic acid
10-Camphorsulfonic acid
Acetylharpagide
Scopolamine butylbromide
Tomelukast
1-Naphthol
4-(benzoylamino)-3-hydroxybutanoic acid
BMP (3:0/9:0)
Proline-hydroxyproline
Convallatoxin
Procyanidin A3
Danshenol C
LPE 16:4
20(S)-Protopanaxadiol
1-(4-benzhydrylpiperidino)-2,2,2-trichloroethan-1-one
Monolaurin
3-Hydroxy-glabrol
Kanosamine
Gentisinic acid
N'-p-coumaroylspermine
LPC 16:3
LPC 16:4
Jasmonic acid
Unigene0098818

B

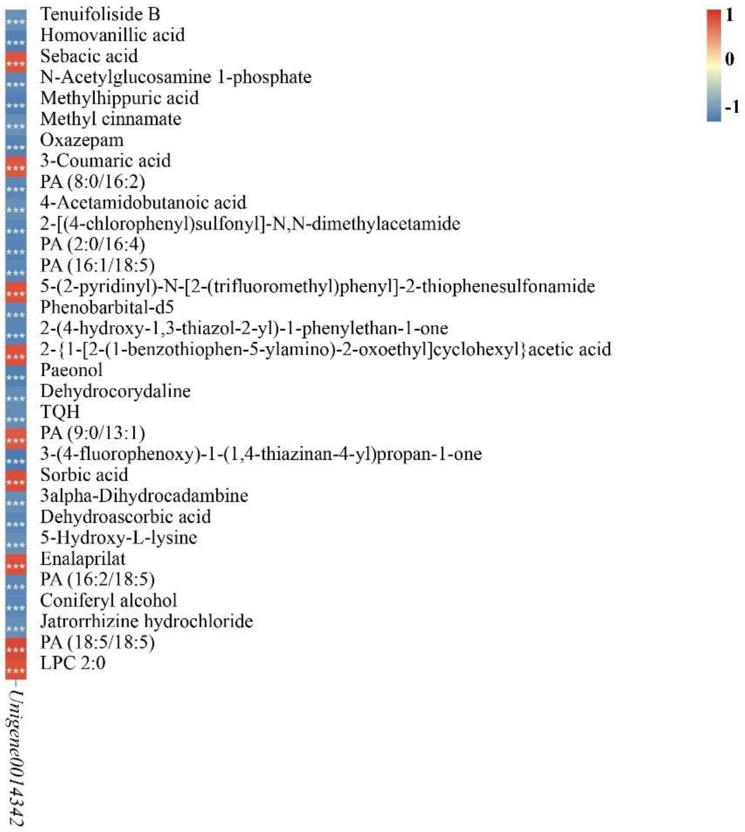
Tenuifoliside B
Homovanillic acid
Sebacic acid
N-Acetylglucosamine 1-phosphate
Methylhippuric acid
Methyl cinnamate
Oxazepam
3-Coumaric acid
PA (8:0/16:2)
4-Acetamidobutanoic acid
2-[(4-chlorophenyl)sulfonyl]-N,N-dimethylacetamide
PA (2:0/16:4)
PA (16:1/18:5)
5-(2-pyridinyl)-N-[2-(trifluoromethyl)phenyl]-2-thiophenesulfonamide
Phenobarbital-d5
2-(4-hydroxy-1,3-thiazol-2-yl)-1-phenylethan-1-one
2-{1-[2-(1-benzothiophen-5-ylamino)-2-oxoethyl]cyclohexyl}acetic acid
Paeonol
Dehydrocorydaline
TQH
PA (9:0/13:1)
3-(4-fluorophenoxy)-1-(1,4-thiazinan-4-yl)propan-1-one
Sorbic acid
3alpha-Dihydrocadambine
Dehydroascorbic acid
5-Hydroxy-L-lysine
Enalaprilat
PA (16:2/18:5)
Coniferyl alcohol
Jatrorrhizine hydrochloride
PA (18:5/18:5)
LPC 2:0
Unigene0014342
1
0
-1

C

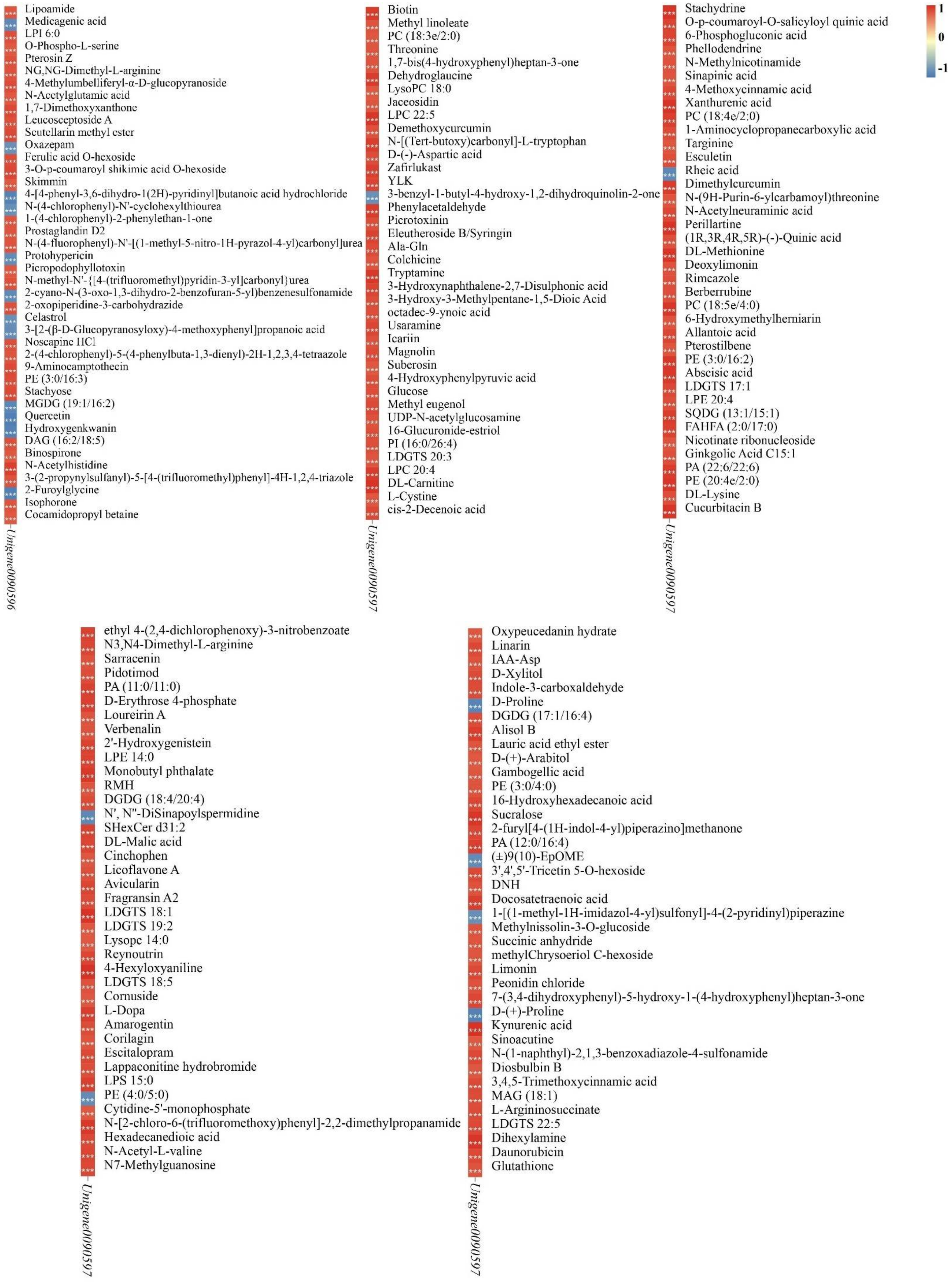

1
0
-1
Lipoamide
Medicagenic acid
LPI 6:0
O-Phospho-L-serine
Pterosin Z
NG,NG-Dimethyl-L-arginine
4-Methylumbelliferyl-α-D-glucopyranoside
N-Acetylglutamic acid
1,7-Dimethoxyxanthone
Leucosceptoside A
Scutellarin methyl ester
Oxazepam
Ferulic acid O-hexoside
3-O-p-coumaroyl shikimic acid O-hexoside
Skimmin
4-[4-phenyl-3,6-dihydro-1(2H)-pyridinyl]butanoic acid hydrochloride
N-(4-chlorophenyl)-N'-cyclohexylthiourea
1-(4-chlorophenyl)-2-phenylethan-1-one
Prostaglandin D2
N-(4-fluorophenyl)-N'-[(1-methyl-5-nitro-1H-pyrazol-4-yl)carbonyl]urea
Protohypericin
Picropodophyllotoxin
N-methyl-N'-{[4-(trifluoromethyl)pyridin-3-yl]carbonyl}urea
2-cyano-N-(3-oxo-1,3-dihydro-2-benzofuran-5-yl)benzenesulfonamide
2-oxopiperidine-3-carbohydrazide
Celastrol
3-[2-(β-D-Glucopyranosyloxy)-4-methoxyphenyl]propanoic acid
Noscapine HCl
2-(4-chlorophenyl)-5-(4-phenylbuta-1,3-dienyl)-2H-1,2,3,4-tetraazole
9-Aminocamptothecin
PE (3:0/16:3)
Stachyose
MGDG (19:1/16:2)
Quercetin
Hydroxygenkwanin
DAG (16:2/18:5)
Binospirone
N-Acetylhistidine
3-(2-propynylsulfanyl)-5-[4-(trifluoromethyl)phenyl]-4H-1,2,4-triazole
2-Furoylglycine
Isophorone
Cocamidopropyl betaine
Unigene0090596
Biotin
Methyl linoleate
PC (18:3e/2:0)
Threonine
1,7-bis(4-hydroxyphenyl)heptan-3-one
Dehydroglaucine
LysoPC 18:0
Jaceosidin
LPC 22:5
Demethoxycurcumin
N-[(Tert-butoxy)carbonyl]-L-tryptophan
D-(-)-Aspartic acid
Zafirlukast
YLK
3-benzyl-1-butyl-4-hydroxy-1,2-dihydroquinolin-2-one
Phenylacetaldehyde
Picrotoxinin
Eleutheroside B/Syringin
Ala-Gln
Colchicine
Tryptamine
3-Hydroxynaphthalene-2,7-Disulphonic acid
3-Hydroxy-3-Methylpentane-1,5-Dioic Acid
octadec-9-ynoic acid
Usaramine
Icariin
Magnolin
Suberosin
4-Hydroxyphenylpyruvic acid
Glucose
Methyl eugenol
UDP-N-acetylglucosamine
16-Glucuronide-estriol
PI (16:0/26:4)
LDGTS 20:3
LPC 20:4
DL-Carnitine
L-Cystine
cis-2-Decenoic acid
Unigene0090597
Stachydrine
O-p-coumaroyl-O-salicyloyl quinic acid
6-Phosphogluconic acid
Phellodendrine
N-Methylnicotinamide
Sinapinic acid
4-Methoxycinnamic acid
Xanthurenic acid
PC (18:4e/2:0)
1-Aminocyclopropanecarboxylic acid
Targinine
Esculetin
Rheic acid
Dimethylcurcumin
N-(9H-Purin-6-ylcarbamoyl)threonine
N-Acetylneuraminic acid
Perillartine
(1R,3R,4R,5R)-(-)-Quinic acid
DL-Methionine
Deoxylimonin
Rimcazole
Berberrubine
PC (18:5e/4:0)
6-Hydroxymethylherniarin
Allantoic acid
Pterostilbene
PE (3:0/16:2)
Abscisic acid
LDGTS 17:1
LPE 20:4
SQDG (13:1/15:1)
FAHFA (2:0/17:0)
Nicotinate ribonucleoside
Ginkgolic Acid C15:1
PA (22:6/22:6)
PE (20:4e/2:0)
DL-Lysine
Cucurbitacin B
Unigene0090597
ethyl 4-(2,4-dichlorophenoxy)-3-nitrobenzoate
N3,N4-Dimethyl-L-arginine
Sarracenin
Pidotimod
PA (11:0/11:0)
D-Erythrose 4-phosphate
Loureirin A
Verbenalin
2'-Hydroxygenistein
LPE 14:0
Monobutyl phthalate
RMH
DGDG (18:4/20:4)
N', N''-DiSinapoylspermidine
SHexCer d31:2
DL-Malic acid
Cinchophen
Licoflavone A
Avicularin
Fragransin A2
LDGTS 18:1
LDGTS 19:2
Lysopc 14:0
Reynoutrin
4-Hexyloxyaniline
LDGTS 18:5
Cornuside
L-Dopa
Amarogentin
Corilagin
Escitalopram
Lappaconitine hydrobromide
LPS 15:0
PE (4:0/5:0)
Cytidine-5'-monophosphate
N-[2-chloro-6-(trifluoromethoxy)phenyl]-2,2-dimethylpropanamide
Hexadecanedioic acid
N-Acetyl-L-valine
N7-Methylguanosine
Unigene0090597
Oxypeucedanin hydrate
Linarin
IAA-Asp
D-Xylitol
Indole-3-carboxaldehyde
D-Proline
DGDG (17:1/16:4)
Alisol B
Lauric acid ethyl ester
D-(+)-Arabitol
Gambogellic acid
PE (3:0/4:0)
16-Hydroxyhexadecanoic acid
Sucralose
2-furyl[4-(1H-indol-4-yl)piperazino]methanone
PA (12:0/16:4)
(±)9(10)-EpOME
3',4',5'-Tricetin 5-O-hexoside
DNH
Docosatetraenoic acid
1-[(1-methyl-1H-imidazol-4-yl)sulfonyl]-4-(2-pyridinyl)piperazine
Methylnissolin-3-O-glucoside
Succinic anhydride
methylChrysoeriol C-hexoside
Limonin
Peonidin chloride
7-(3,4-dihydroxyphenyl)-5-hydroxy-1-(4-hydroxyphenyl)heptan-3-one
D-(+)-Proline
Kynurenic acid
Sinoacutine
N-(1-naphthyl)-2,1,3-benzoxadiazole-4-sulfonamide
Diosbulbin B
3,4,5-Trimethoxycinnamic acid
MAG (18:1)
L-Argininosuccinate
LDGTS 22:5
Dihexylamine
Daunorubicin
Glutathione
Unigene0090597

D

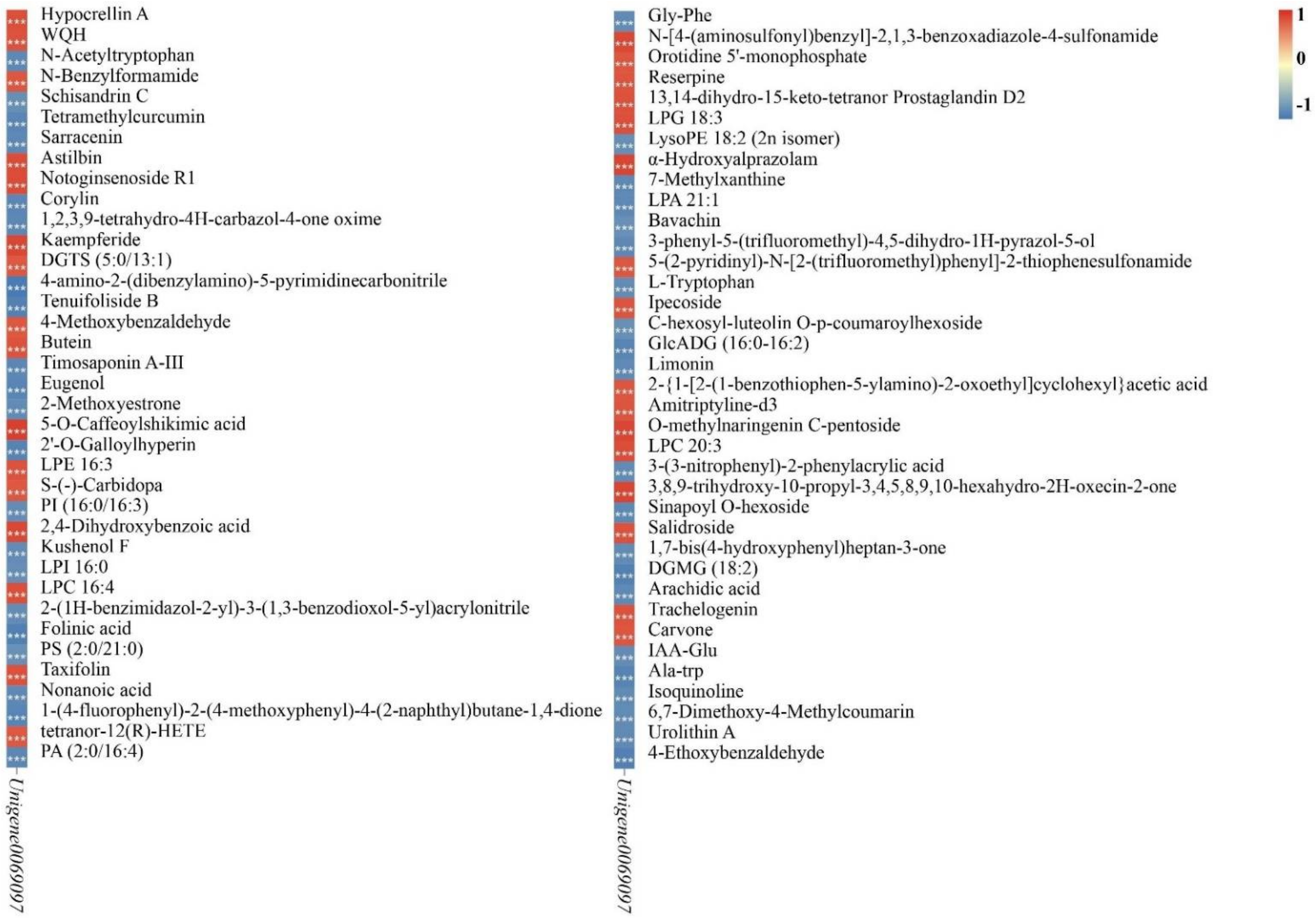
Hypocrellin A
WQH
N-Acetyltryptophan
N-Benzylformamide
Schisandrin C
Tetramethylcurcumin
Sarracenin
Astilbin
Notoginsenoside R1
Corylin
1,2,3,9-tetrahydro-4H-carbazol-4-one oxime
Kaempferide
DGTS (5:0/13:1)
4-amino-2-(dibenzylamino)-5-pyrimidinecarbonitrile
Tenuifoliside B
4-Methoxybenzaldehyde
Butein
Timosaponin A-III
Eugenol
2-Methoxyestrone
5-O-Caffeoylshikimic acid
2'-O-Galloylhyperin
LPE 16:3
S-(-)-Carbidopa
PI (16:0/16:3)
2,4-Dihydroxybenzoic acid
Kushenol F
LPI 16:0
LPC 16:4
2-(1H-benzimidazol-2-yl)-3-(1,3-benzodioxol-5-yl)acrylonitrile
Folinic acid
PS (2:0/21:0)
Taxifolin
Nonanoic acid
1-(4-fluorophenyl)-2-(4-methoxyphenyl)-4-(2-naphthyl)butane-1,4-dione
tetranor-12(R)-HETE
PA (2:0/16:4)
Unigene0069097
Gly-Phe
N-[4-(aminosulfonyl)benzyl]-2,1,3-benzoxadiazole-4-sulfonamide
Orotidine 5'-monophosphate
Reserpine
13,14-dihydro-15-keto-tetranor Prostaglandin D2
LPG 18:3
LysoPE 18:2 (2n isomer)
α-Hydroxyalprazolam
7-Methylxanthine
LPA 21:1
Bavachin
3-phenyl-5-(trifluoromethyl)-4,5-dihydro-1H-pyrazol-5-ol
5-(2-pyridinyl)-N-[2-(trifluoromethyl)phenyl]-2-thiophenesulfonamide
L-Tryptophan
Ipecoside
C-hexosyl-luteolin O-p-coumaroylhexoside
GlcADG (16:0-16:2)
Limonin
2-{1-[2-(1-benzothiophen-5-ylamino)-2-oxoethyl]cyclohexyl}acetic acid
Amitriptyline-d3
O-methylnaringenin C-pentoside
LPC 20:3
3-(3-nitrophenyl)-2-phenylacrylic acid
3,8,9-trihydroxy-10-propyl-3,4,5,8,9,10-hexahydro-2H-oxecin-2-one
Sinapoyl O-hexoside
Salidroside
1,7-bis(4-hydroxyphenyl)heptan-3-one
DGMG (18:2)
Arachidic acid
Trachelogenin
Carvone
IAA-Glu
Ala-trp
Isoquinoline
6,7-Dimethoxy-4-Methylcoumarin
Urolithin A
4-Ethoxybenzaldehyde
Unigene0069097
1
0
-1

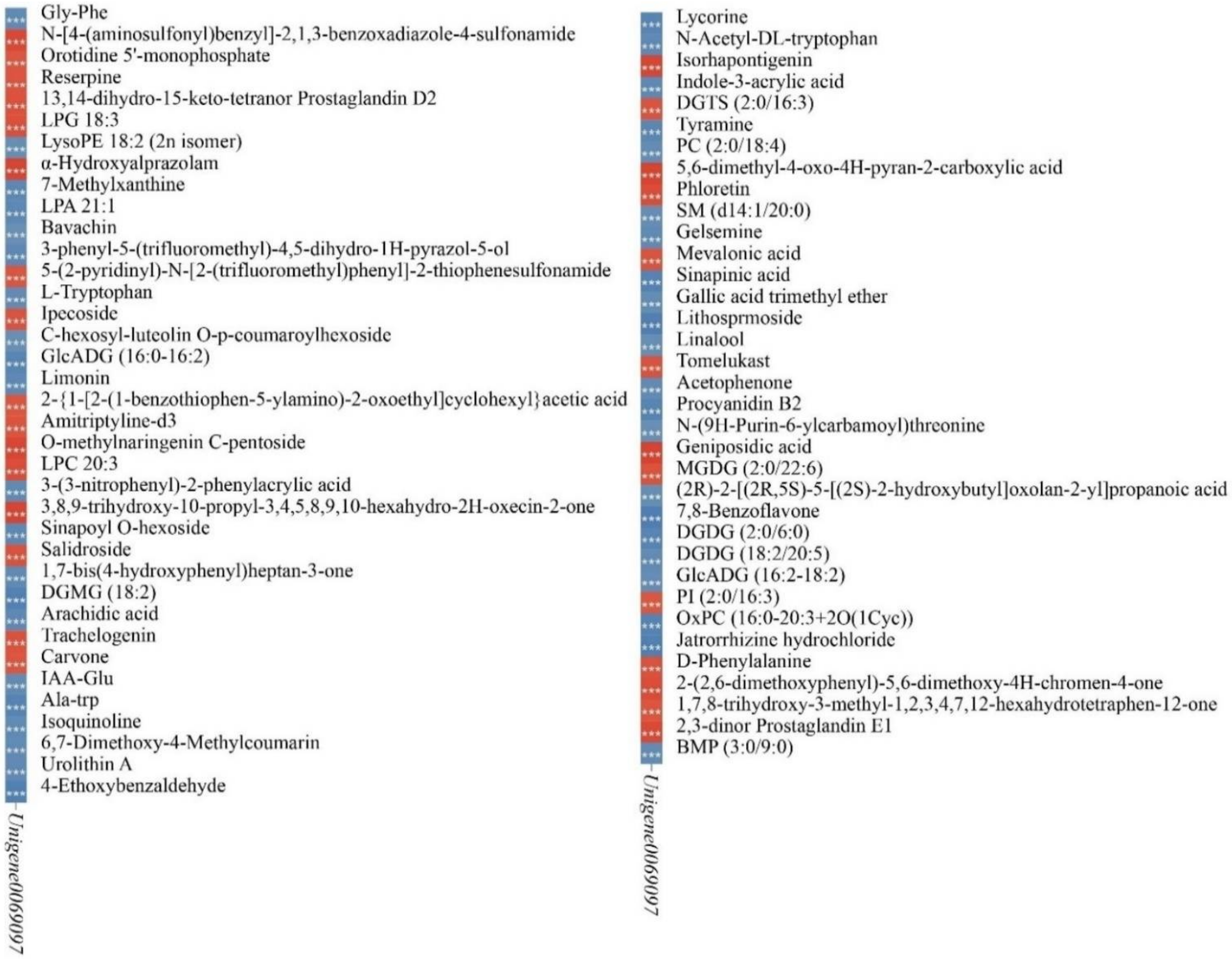
Gly-Phe
N-[4-(aminosulfonyl)benzyl]-2,1,3-benzoxadiazole-4-sulfonamide
Orotidine 5'-monophosphate
Reserpine
13,14-dihydro-15-keto-tetranor Prostaglandin D2
LPG 18:3
LysoPE 18:2 (2n isomer)
α-Hydroxyalprazolam
7-Methylxanthine
LPA 21:1
Bavachin
3-phenyl-5-(trifluoromethyl)-4,5-dihydro-1H-pyrazol-5-ol
5-(2-pyridinyl)-N-[2-(trifluoromethyl)phenyl]-2-thiophenesulfonamide
L-Tryptophan
Ipecoside
C-hexosyl-luteolin O-p-coumaroylhexoside
GlcADG (16:0-16:2)
Limonin
2-{1-[2-(1-benzothiophen-5-ylamino)-2-oxoethyl]cyclohexyl}acetic acid
Amitriptyline-d3
O-methylnaringenin C-pentoside
LPC 20:3
3-(3-nitrophenyl)-2-phenylacrylic acid
3,8,9-trihydroxy-10-propyl-3,4,5,8,9,10-hexahydro-2H-oxecin-2-one
Sinapoyl O-hexoside
Salidroside
1,7-bis(4-hydroxyphenyl)heptan-3-one
DGMG (18:2)
Arachidic acid
Trachelogenin
Carvone
IAA-Glu
Ala-trp
Isoquinoline
6,7-Dimethoxy-4-Methylcoumarin
Urolithin A
4-Ethoxybenzaldehyde
Unigene0069097
Lycorine
N-Acetyl-DL-tryptophan
Isorhapontigenin
Indole-3-acrylic acid
DGTS (2:0/16:3)
Tyramine
PC (2:0/18:4)
5,6-dimethyl-4-oxo-4H-pyran-2-carboxylic acid
Phloretin
SM (d14:1/20:0)
Gelsemine
Mevalonic acid
Sinapinic acid
Gallic acid trimethyl ether
Lithosprmoside
Linalool
Tomelukast
Acetophenone
Procyanidin B2
N-(9H-Purin-6-ylcarbamoyl)threonine
Geniposidic acid
MGDG (2:0/22:6)
(2R)-2-[(2R,5S)-5-[(2S)-2-hydroxybutyl]oxolan-2-yl]propanoic acid
7,8-Benzoflavone
DGDG (2:0/6:0)
DGDG (18:2/20:5)
GlcADG (16:2-18:2)
PI (2:0/16:3)
OxPC (16:0-20:3+2O(1Cyc))
Jatrorrhizine hydrochloride
D-Phenylalanine
2-(2,6-dimethoxyphenyl)-5,6-dimethoxy-4H-chromen-4-one
1,7,8-trihydroxy-3-methyl-1,2,3,4,7,12-hexahydrotetraphen-12-one
2,3-dinor Prostaglandin E1
BMP (3:0/9:0)
Unigene0069097

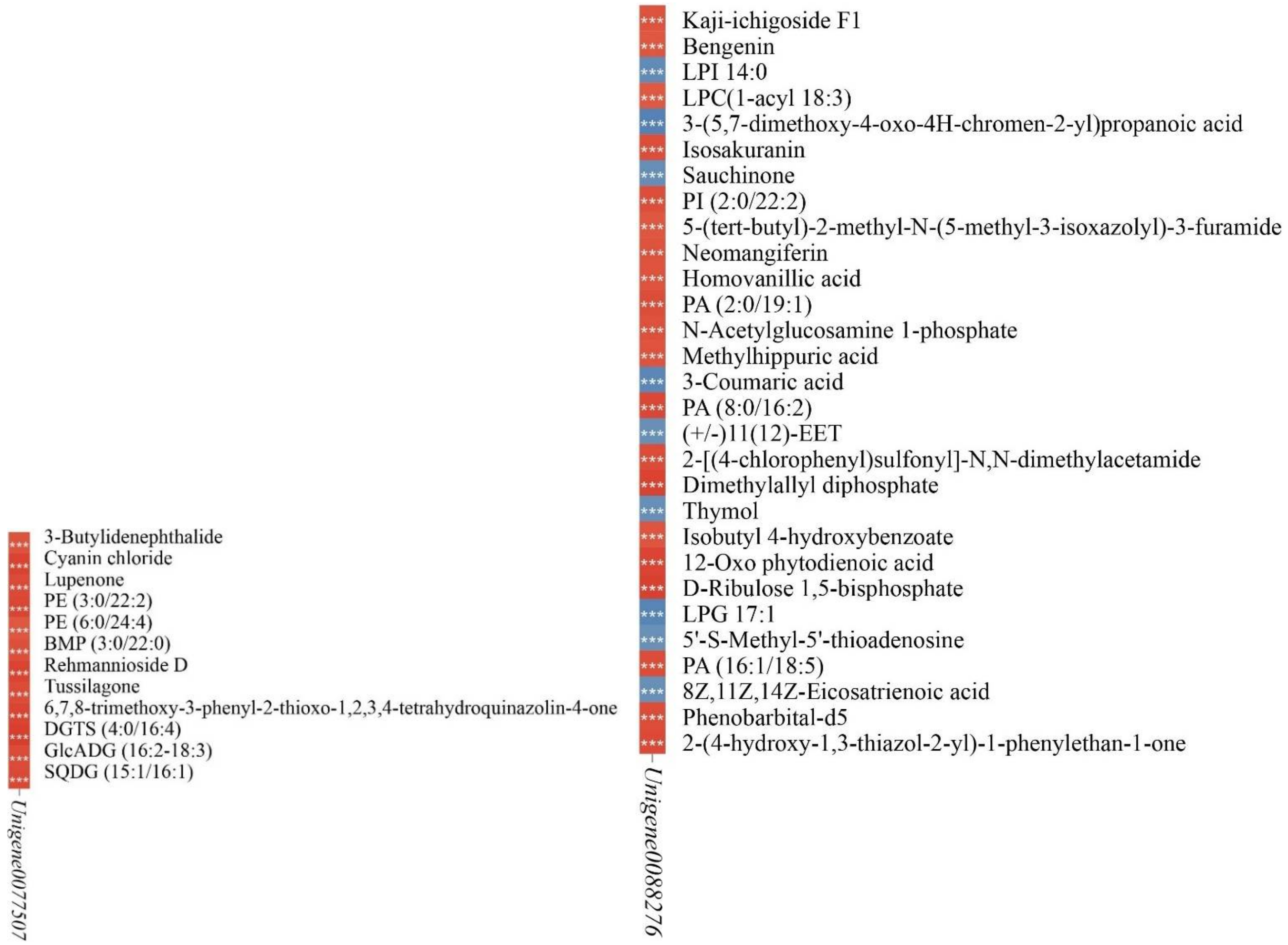
3-Butylidenephthalide
Cyanin chloride
Lupenone
PE (3:0/22:2)
PE (6:0/24:4)
BMP (3:0/22:0)
Rehmannioside D
Tussilagone
6,7,8-trimethoxy-3-phenyl-2-thioxo-1,2,3,4-tetrahydroquinazolin-4-one
DGTS (4:0/16:4)
GlcADG (16:2-18:3)
SQDG (15:1/16:1)
Unigene0077507
Kaji-ichigoside F1
Bengenin
LPI 14:0
LPC(1-acyl 18:3)
3-(5,7-dimethoxy-4-oxo-4H-chromen-2-yl)propanoic acid
Isosakuranin
Sauchinone
PI (2:0/22:2)
5-(tert-butyl)-2-methyl-N-(5-methyl-3-isoxazolyl)-3-furamide
Neomangiferin
Homovanillic acid
PA (2:0/19:1)
N-Acetylglucosamine 1-phosphate
Methylhippuric acid
3-Coumaric acid
PA (8:0/16:2)
(+/-)11(12)-EET
2-[(4-chlorophenyl)sulfonyl]-N,N-dimethylacetamide
Dimethylallyl diphosphate
Thymol
Isobutyl 4-hydroxybenzoate
12-Oxo phytodienoic acid
D-Ribulose 1,5-bisphosphate
LPG 17:1
5'-S-Methyl-5'-thioadenosine
PA (16:1/18:5)
8Z,11Z,14Z-Eicosatrienoic acid
Phenobarbital-d5
2-(4-hydroxy-1,3-thiazol-2-yl)-1-phenylethan-1-one
Unigene0088276

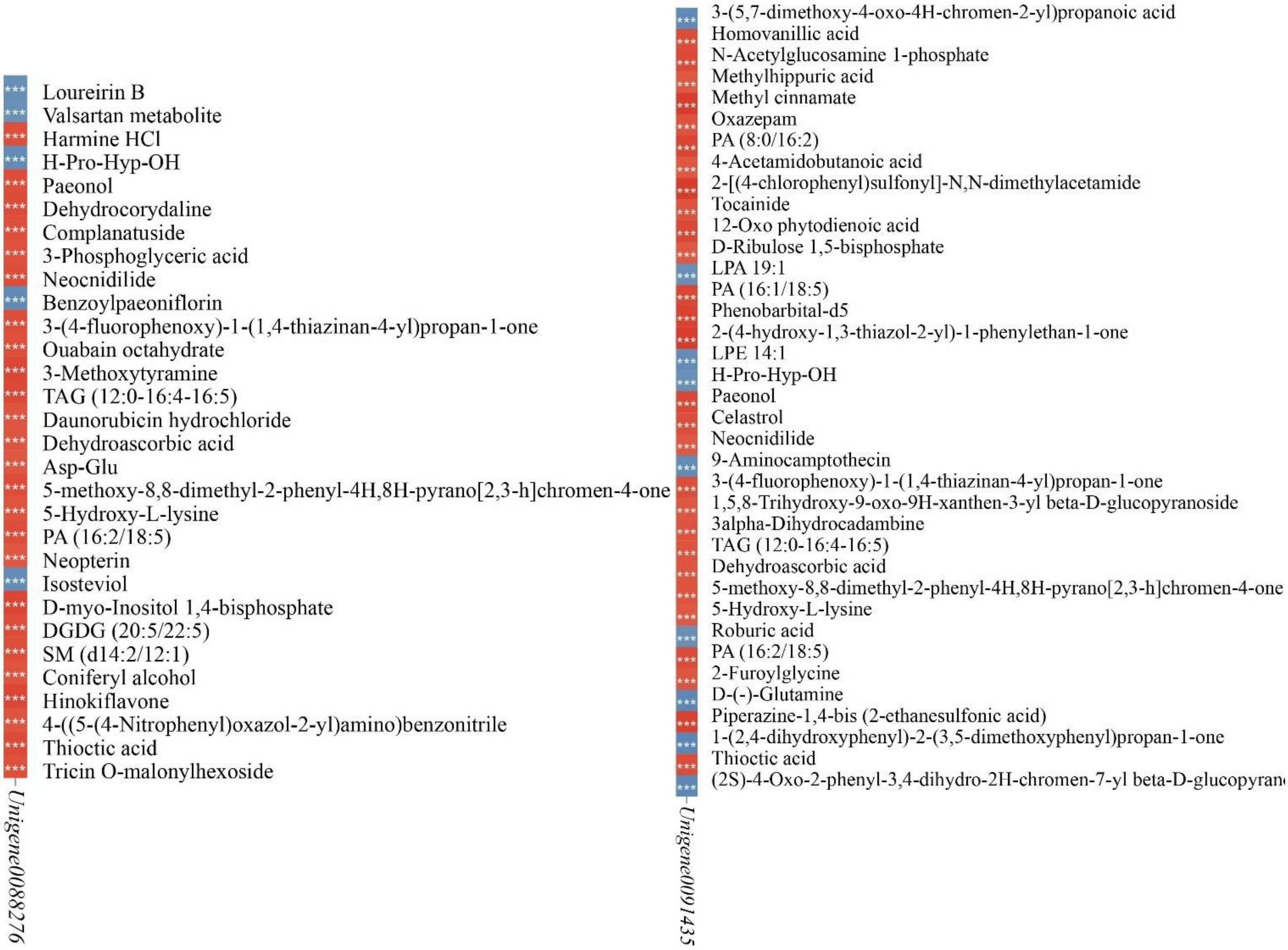
Loureirin B
Valsartan metabolite
Harmine HCl
H-Pro-Hyp-OH
Paeonol
Dehydrocorydaline
Complanatuside
3-Phosphoglyceric acid
Neocnidilide
Benzoylpaeoniflorin
3-(4-fluorophenoxy)-1-(1,4-thiazinan-4-yl)propan-1-one
Ouabain octahydrate
3-Methoxytyramine
TAG (12:0-16:4-16:5)
Daunorubicin hydrochloride
Dehydroascorbic acid
Asp-Glu
5-methoxy-8,8-dimethyl-2-phenyl-4H,8H-pyrano[2,3-h]chromen-4-one
5-Hydroxy-L-lysine
PA (16:2/18:5)
Neopterin
Isosteviol
D-myo-Inositol 1,4-bisphosphate
DGDG (20:5/22:5)
SM (d14:2/12:1)
Coniferyl alcohol
Hinokiflavone
4-((5-(4-Nitrophenyl)oxazol-2-yl)amino)benzonitrile
Thioctic acid
Tricin O-malonylhexoside
Unigene0088276
3-(5,7-dimethoxy-4-oxo-4H-chromen-2-yl)propanoic acid
Homovanillic acid
N-Acetylglucosamine 1-phosphate
Methylhippuric acid
Methyl cinnamate
Oxazepam
PA (8:0/16:2)
4-Acetamidobutanoic acid
2-[(4-chlorophenyl)sulfonyl]-N,N-dimethylacetamide
Tocainide
12-Oxo phytodienoic acid
D-Ribulose 1,5-bisphosphate
LPA 19:1
PA (16:1/18:5)
Phenobarbital-d5
2-(4-hydroxy-1,3-thiazol-2-yl)-1-phenylethan-1-one
LPE 14:1
H-Pro-Hyp-OH
Paeonol
Celastrol
Neocnidilide
9-Aminocamptothecin
3-(4-fluorophenoxy)-1-(1,4-thiazinan-4-yl)propan-1-one
1,5,8-Trihydroxy-9-oxo-9H-xanthen-3-yl beta-D-glucopyranoside
3alpha-Dihydrocadambine
TAG (12:0-16:4-16:5)
Dehydroascorbic acid
5-methoxy-8,8-dimethyl-2-phenyl-4H,8H-pyrano[2,3-h]chromen-4-one
5-Hydroxy-L-lysine
Roburic acid
PA (16:2/18:5)
2-Furoylglycine
D-(-)-Glutamine
Piperazine-1,4-bis (2-ethanesulfonic acid)
1-(2,4-dihydroxyphenyl)-2-(3,5-dimethoxyphenyl)propan-1-one
Thioctic acid
(2S)-4-Oxo-2-phenyl-3,4-dihydro-2H-chromen-7-yl beta-D-glucopyran
Unigene0091435

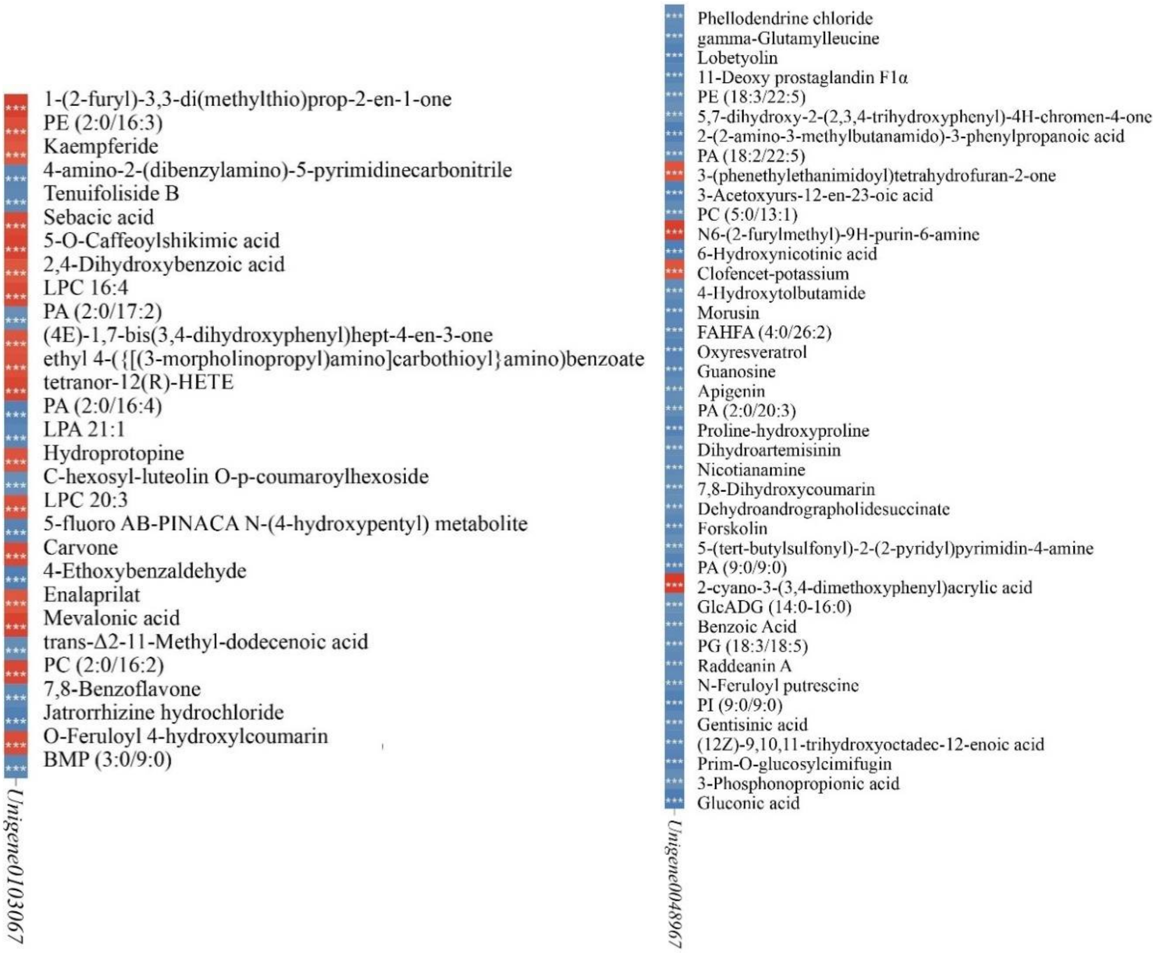
1-(2-furyl)-3,3-di(methylthio)prop-2-en-1-one
PE (2:0/16:3)
Kaempferide
4-amino-2-(dibenzylamino)-5-pyrimidinecarbonitrile
Tenuifoliside B
Sebacic acid
5-O-Caffeoylshikimic acid
2,4-Dihydroxybenzoic acid
LPC 16:4
PA (2:0/17:2)
(4E)-1,7-bis(3,4-dihydroxyphenyl)hept-4-en-3-one
ethyl 4-({[(3-morpholinopropyl)amino]carbothioyl}amino)benzoate
tetranor-12(R)-HETE
PA (2:0/16:4)
LPA 21:1
Hydroprotopine
C-hexosyl-luteolin O-p-coumaroylhexoside
LPC 20:3
5-fluoro AB-PINACA N-(4-hydroxypentyl) metabolite
Carvone
4-Ethoxybenzaldehyde
Enalaprilat
Mevalonic acid
trans-Δ2-11-Methyl-dodecenoic acid
PC (2:0/16:2)
7,8-Benzoflavone
Jatrorrhizine hydrochloride
O-Feruloyl 4-hydroxylcoumarin
BMP (3:0/9:0)
Unigene0103067
Phellodendrine chloride
gamma-Glutamylleucine
Lobetyolin
11-Deoxy prostaglandin F1α
PE (18:3/22:5)
5,7-dihydroxy-2-(2,3,4-trihydroxyphenyl)-4H-chromen-4-one
2-(2-amino-3-methylbutanamido)-3-phenylpropanoic acid
PA (18:2/22:5)
3-(phenethylethanimidoyl)tetrahydrofuran-2-one
3-Acetoxyurs-12-en-23-oic acid
PC (5:0/13:1)
N6-(2-furylmethyl)-9H-purin-6-amine
6-Hydroxynicotinic acid
Clofencet-potassium
4-Hydroxytolbutamide
Morusin
FAHFA (4:0/26:2)
Oxyresveratrol
Guanosine
Apigenin
PA (2:0/20:3)
Proline-hydroxyproline
Dihydroartemisinin
Nicotianamine
7,8-Dihydroxycoumarin
Dehydroandrographolidesuccinate
Forskolin
5-(tert-butylsulfonyl)-2-(2-pyridyl)pyrimidin-4-amine
PA (9:0/9:0)
2-cyano-3-(3,4-dimethoxyphenyl)acrylic acid
GlcADG (14:0-16:0)
Benzoic Acid
PG (18:3/18:5)
Raddeanin A
N-Feruloyl putrescine
PI (9:0/9:0)
Gentisinic acid
(12Z)-9,10,11-trihydroxyoctadec-12-enoic acid
Prim-O-glucosylcimifugin
3-Phosphonopropionic acid
Gluconic acid
Unigene0048967

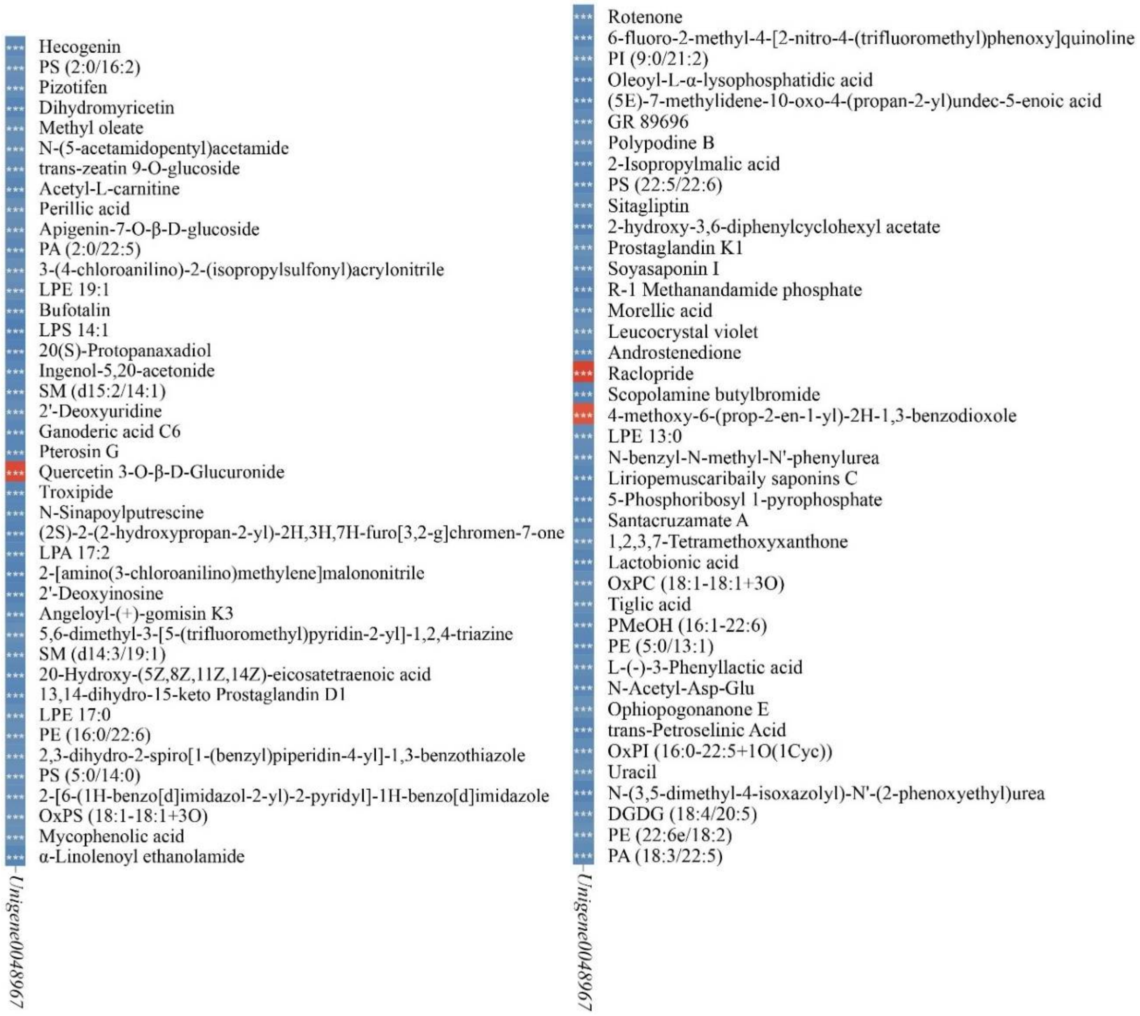
Hecogenin
PS (2:0/16:2)
Pizotifen
Dihydromyricetin
Methyl oleate
N-(5-acetamidopentyl)acetamide
trans-zeatin 9-O-glucoside
Acetyl-L-carnitine
Perillic acid
Apigenin-7-O-β-D-glucoside
PA (2:0/22:5)
3-(4-chloroanilino)-2-(isopropylsulfonyl)acrylonitrile
LPE 19:1
Bufotalin
LPS 14:1
20(S)-Protopanaxadiol
Ingenol-5,20-acetonide
SM (d15:2/14:1)
2'-Deoxyuridine
Ganoderic acid C6
Pterosin G
Quercetin 3-O-β-D-Glucuronide
Troxipide
N-Sinapoylputrescine
(2S)-2-(2-hydroxypropan-2-yl)-2H,3H,7H-furo[3,2-g]chromen-7-one
LPA 17:2
2-[amino(3-chloroanilino)methylene]malononitrile
2'-Deoxyinosine
Angeloyl-(+)-gomisin K3
5,6-dimethyl-3-[5-(trifluoromethyl)pyridin-2-yl]-1,2,4-triazine
SM (d14:3/19:1)
20-Hydroxy-(5Z,8Z,11Z,14Z)-eicosatetraenoic acid
13,14-dihydro-15-keto Prostaglandin D1
LPE 17:0
PE (16:0/22:6)
2,3-dihydro-2-spiro[1-(benzyl)piperidin-4-yl]-1,3-benzothiazole
PS (5:0/14:0)
2-[6-(1H-benzo[d]imidazol-2-yl)-2-pyridyl]-1H-benzo[d]imidazole
OxPS (18:1-18:1+3O)
Mycophenolic acid
α-Linolenoyl ethanolamide
Unigene0048967
Rotenone
6-fluoro-2-methyl-4-[2-nitro-4-(trifluoromethyl)phenoxy]quinoline
PI (9:0/21:2)
Oleoyl-L-α-lysophosphatidic acid
(5E)-7-methylidene-10-oxo-4-(propan-2-yl)undec-5-enoic acid
GR 89696
Polypodine B
2-Isopropylmalic acid
PS (22:5/22:6)
Sitagliptin
2-hydroxy-3,6-diphenylcyclohexyl acetate
Prostaglandin K1
Soyasaponin I
R-1 Methanandamide phosphate
Morellic acid
Leucocrystal violet
Androstenedione
Raclopride
Scopolamine butylbromide
4-methoxy-6-(prop-2-en-1-yl)-2H-1,3-benzodioxole
LPE 13:0
N-benzyl-N-methyl-N'-phenylurea
Liriopemuscaribaily saponins C
5-Phosphoribosyl 1-pyrophosphate
Santacruzamate A
1,2,3,7-Tetramethoxyxanthone
Lactobionic acid
OxPC (18:1-18:1+3O)
Tiglic acid
PMeOH (16:1-22:6)
PE (5:0/13:1)
L-(-)-3-Phenyllactic acid
N-Acetyl-Asp-Glu
Ophiopogonanone E
trans-Petroselinic Acid
OxPI (16:0-22:5+1O(1Cyc))
Uracil
N-(3,5-dimethyl-4-isoxazolyl)-N'-(2-phenoxyethyl)urea
DGDG (18:4/20:5)
PE (22:6e/18:2)
PA (18:3/22:5)
Unigene0048967

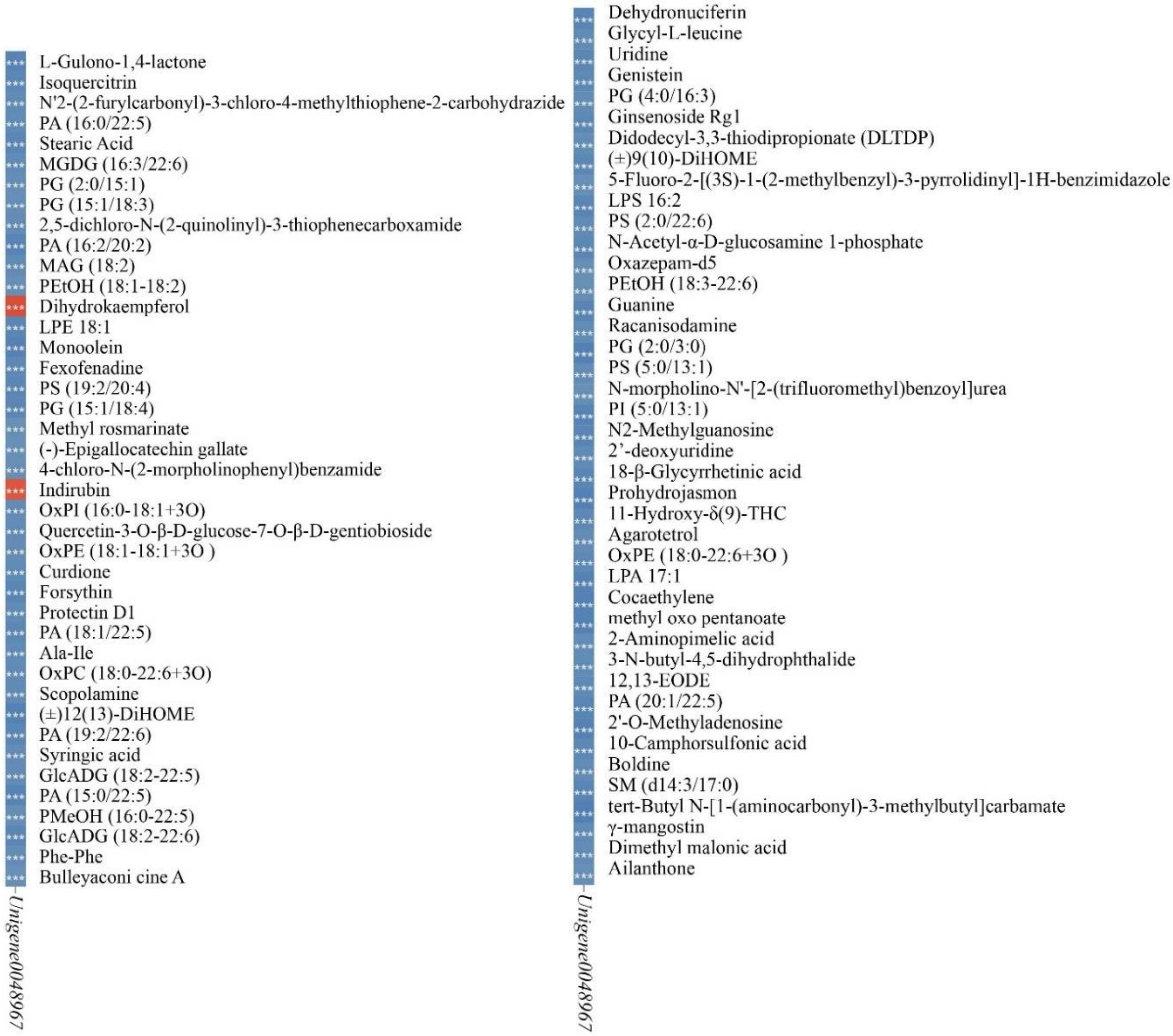
L-Gulono-1,4-lactone
Isoquercitrin
N'2-(2-furylcarbonyl)-3-chloro-4-methylthiophene-2-carbohydrazide
PA (16:0/22:5)
Stearic Acid
MGDG (16:3/22:6)
PG (2:0/15:1)
PG (15:1/18:3)
2,5-dichloro-N-(2-quinolinyl)-3-thiophenecarboxamide
PA (16:2/20:2)
MAG (18:2)
PEtOH (18:1-18:2)
Dihydrokaempferol
LPE 18:1
Monoolein
Fexofenadine
PS (19:2/20:4)
PG (15:1/18:4)
Methyl rosmarinate
(-)-Epigallocatechin gallate
4-chloro-N-(2-morpholinophenyl)benzamide
Indirubin
OxPI (16:0-18:1+3O)
Quercetin-3-O-β-D-glucose-7-O-β-D-gentiobioside
OxPE (18:1-18:1+3O)
Curdione
Forsythin
Protectin D1
PA (18:1/22:5)
Ala-Ile
OxPC (18:0-22:6+3O)
Scopolamine
(±)12(13)-DiHOME
PA (19:2/22:6)
Syringic acid
GlcADG (18:2-22:5)
PA (15:0/22:5)
PMeOH (16:0-22:5)
GlcADG (18:2-22:6)
Phe-Phe
Bulleyaconi cine A
Unigene0048967
Dehydronuciferin
Glycyl-L-leucine
Uridine
Genistein
PG (4:0/16:3)
Ginsenoside Rg1
Didodecyl-3,3-thiodipropionate (DLTDP)
(±)9(10)-DiHOME
5-Fluoro-2-[(3S)-1-(2-methylbenzyl)-3-pyrrolidinyl]-1H-benzimidazole
LPS 16:2
PS (2:0/22:6)
N-Acetyl-α-D-glucosamine 1-phosphate
Oxazepam-d5
PEtOH (18:3-22:6)
Guanine
Racanisodamine
PG (2:0/3:0)
PS (5:0/13:1)
N-morpholino-N'-[2-(trifluoromethyl)benzoyl]urea
PI (5:0/13:1)
N2-Methylguanosine
2'-deoxyuridine
18-β-Glycyrrhetinic acid
Prohydrojasmon
11-Hydroxy-δ(9)-THC
Agarotetrol
OxPE (18:0-22:6+3O)
LPA 17:1
Cocaethylene
methyl oxo pentanoate
2-Aminopimelic acid
3-N-butyl-4,5-dihydrophthalide
12,13-EODE
PA (20:1/22:5)
2'-O-Methyladenosine
10-Camphorsulfonic acid
Boldine
SM (d14:3/17:0)
tert-Butyl N-[1-(aminocarbonyl)-3-methylbutyl]carbamate
γ-mangostin
Dimethyl malonic acid
Ailanthone
Unigene0048967

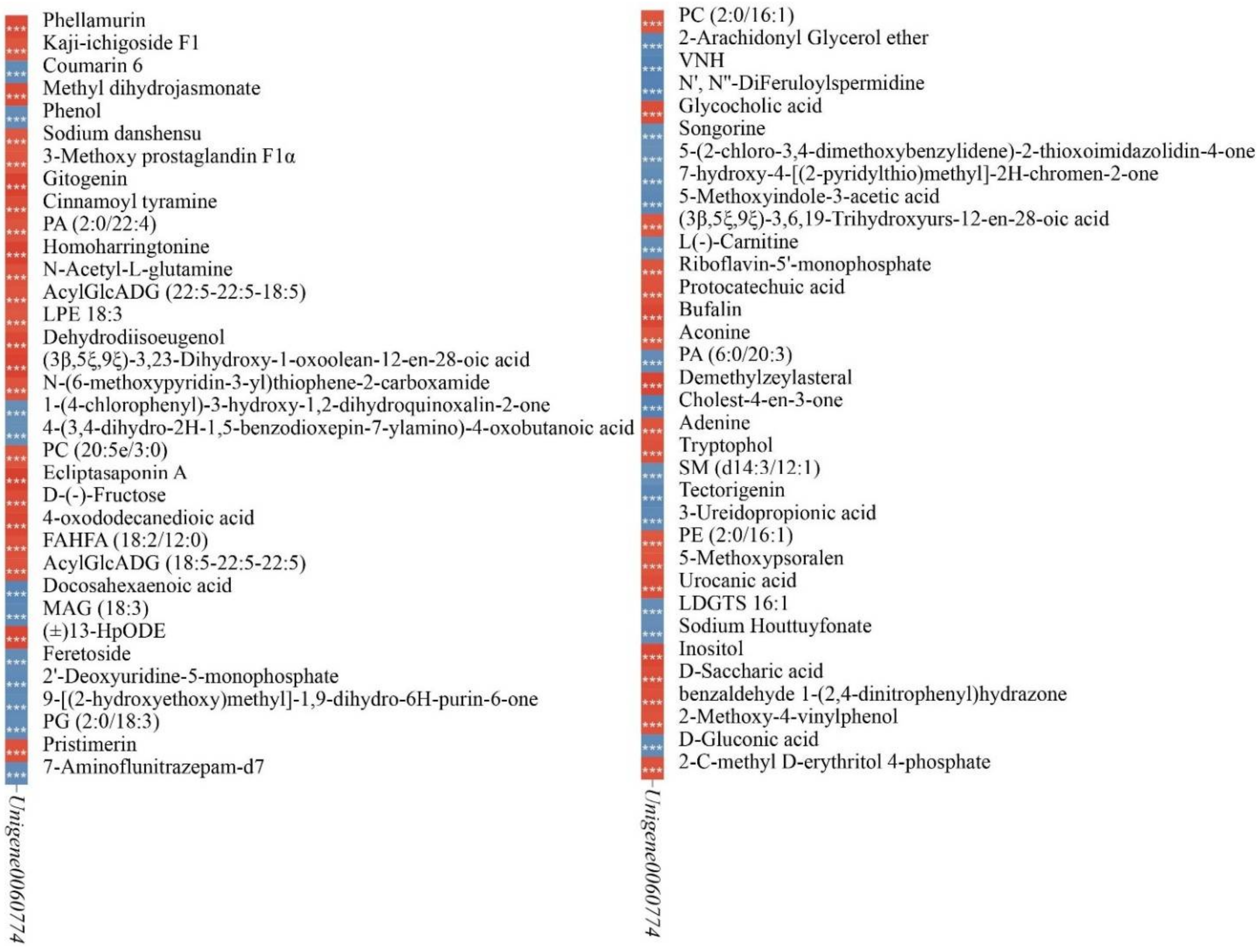
Phellamurin
Kaji-ichigoside F1
Coumarin 6
Methyl dihydrojasmonate
Phenol
Sodium danshensu
3-Methoxy prostaglandin F1α
Gitogenin
Cinnamoyl tyramine
PA (2:0/22:4)
Homoharringtonine
N-Acetyl-L-glutamine
AcylGlcADG (22:5-22:5-18:5)
LPE 18:3
Dehydrodiisoeugenol
(3β,5ξ,9ξ)-3,23-Dihydroxy-1-oxoolean-12-en-28-oic acid
N-(6-methoxypyridin-3-yl)thiophene-2-carboxamide
1-(4-chlorophenyl)-3-hydroxy-1,2-dihydroquinoxalin-2-one
4-(3,4-dihydro-2H-1,5-benzodioxepin-7-ylamino)-4-oxobutanoic acid
PC (20:5e/3:0)
Ecliptasaponin A
D-(-)-Fructose
4-oxododecanedioic acid
FAHFA (18:2/12:0)
AcylGlcADG (18:5-22:5-22:5)
Docosahexaenoic acid
MAG (18:3)
(±)13-HpODE
Feretoside
2'-Deoxyuridine-5-monophosphate
9-[(2-hydroxyethoxy)methyl]-1,9-dihydro-6H-purin-6-one
PG (2:0/18:3)
Pristimerin
7-Aminoflunitrazepam-d7
Unigene0060774
PC (2:0/16:1)
2-Arachidonyl Glycerol ether
VNH
N', N''-DiFeruloylspermidine
Glycocholic acid
Songorine
5-(2-chloro-3,4-dimethoxybenzylidene)-2-thioxoimidazolidin-4-one
7-hydroxy-4-[(2-pyridylthio)methyl]-2H-chromen-2-one
5-Methoxyindole-3-acetic acid
(3β,5ξ,9ξ)-3,6,19-Trihydroxyurs-12-en-28-oic acid
L(-)-Carnitine
Riboflavin-5'-monophosphate
Protocatechuic acid
Bufalin
Aconine
PA (6:0/20:3)
Demethylzeylasteral
Cholest-4-en-3-one
Adenine
Tryptophol
SM (d14:3/12:1)
Tectorigenin
3-Ureidopropionic acid
PE (2:0/16:1)
5-Methoxypsoralen
Urocanic acid
LDGTS 16:1
Sodium Houttuyfonate
Inositol
D-Saccharic acid
benzaldehyde 1-(2,4-dinitrophenyl)hydrazone
2-Methoxy-4-vinylphenol
D-Gluconic acid
2-C-methyl D-erythritol 4-phosphate
Unigene0060774

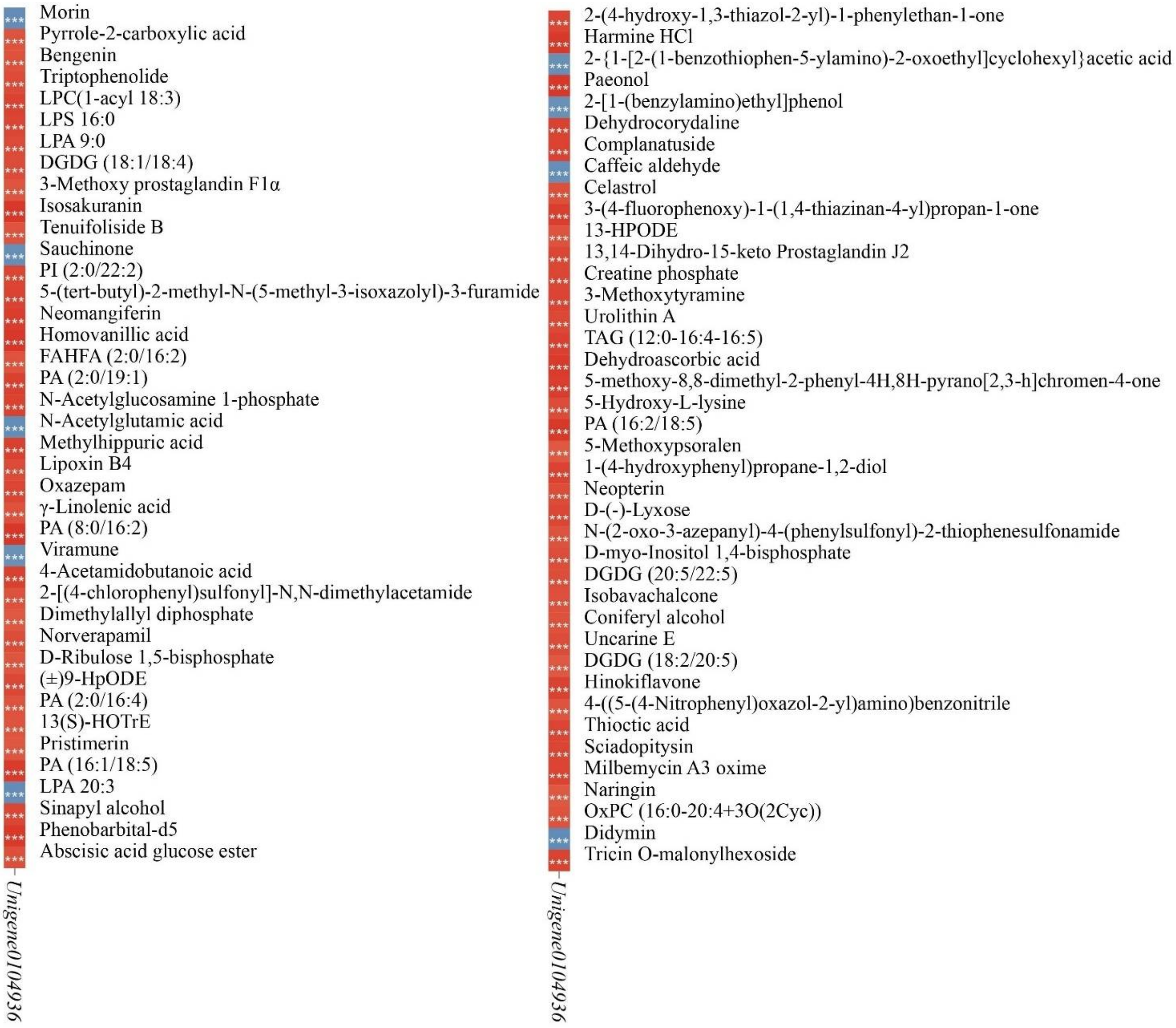

E

Supplementary Figure S2. Heatmap of correlations between major DEGs and metabolites in K^+ channels and transporters

(According to the requirement of the absolute value of Person correlation coefficient |Corr|>0.8, correlation analysis of 17 K^+ channel and transporter-related differentially expressed genes and metabolome numbers of 17 K^+ channel and transporter-related DEGs that were up-regulated by exogenous potassium for 48h and 168h under NaCl stress in *T. ramosissima.* Note: A indicates Shaker channel; B indicates TPK channel; C indicates HAK/KUP/KT transporter; D indicates HKT transporter; E indicates CPAs transporter; $p \geq 0.05$ is not marked; $0.01 < p < 0.05$ is marked as *; $0.001 < p < 0.01$ is marked as **; $p \leq 0.001$ is marked as ***.)
