# Supplementary material for: Transcriptome and Metabonomic Analysis of Tamarix ramosissima Potassium (K+) Channels and Transporters in Response to NaCl Stress
Source: Genes (Basel). 2022 Jul 23;13(8):1313. doi: 10.3390/genes13081313 (PMC9394374; doi:10.3390/genes13081313)
Supplement: Supplementary file 1 [file genes-13-01313-s001.zip › Supplementary Figure S3.pdf]

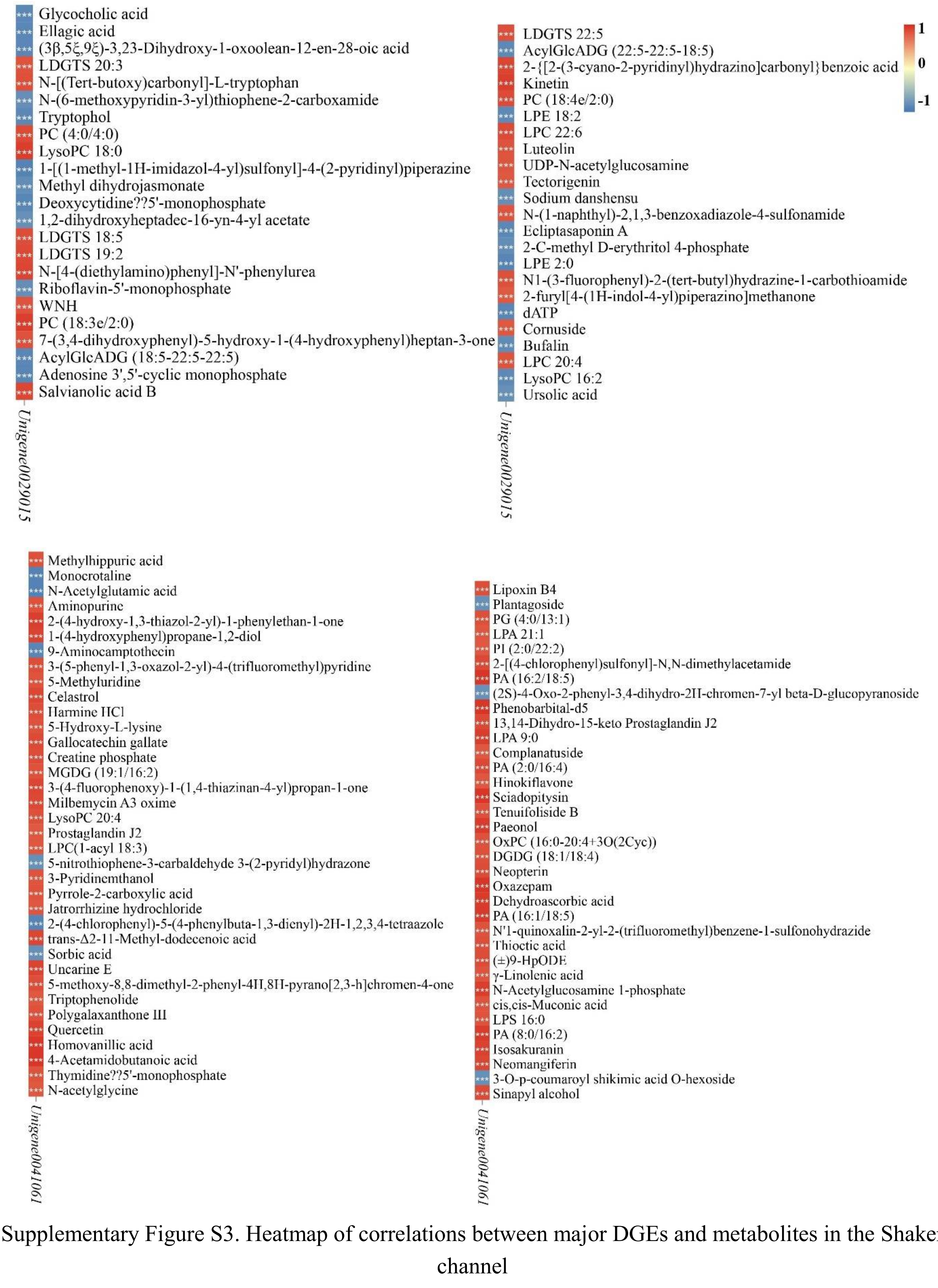

Supplementary Figure S3. Heatmap of correlations between major DGEs and metabolites in the Shaker channel

(According to the requirement of the absolute value of Person correlation coefficient |Corr|>0.8, the related DEGs and metabolome data in the Shaker channel were screened and correlated. $p \geq 0.05$ is not marked; $0.01 < p < 0.05$ is marked as *; $0.001 < p < 0.01$ is marked as **; $p \leq 0.001$ is marked as ***.)
