# Supplementary material for: Transcriptome and Metabonomic Analysis of Tamarix ramosissima Potassium (K+) Channels and Transporters in Response to NaCl Stress
Source: Genes (Basel). 2022 Jul 23;13(8):1313. doi: 10.3390/genes13081313 (PMC9394374; doi:10.3390/genes13081313)
Supplement: Supplementary file 1 [file genes-13-01313-s001.zip › Supplementary Figure S4.pdf]

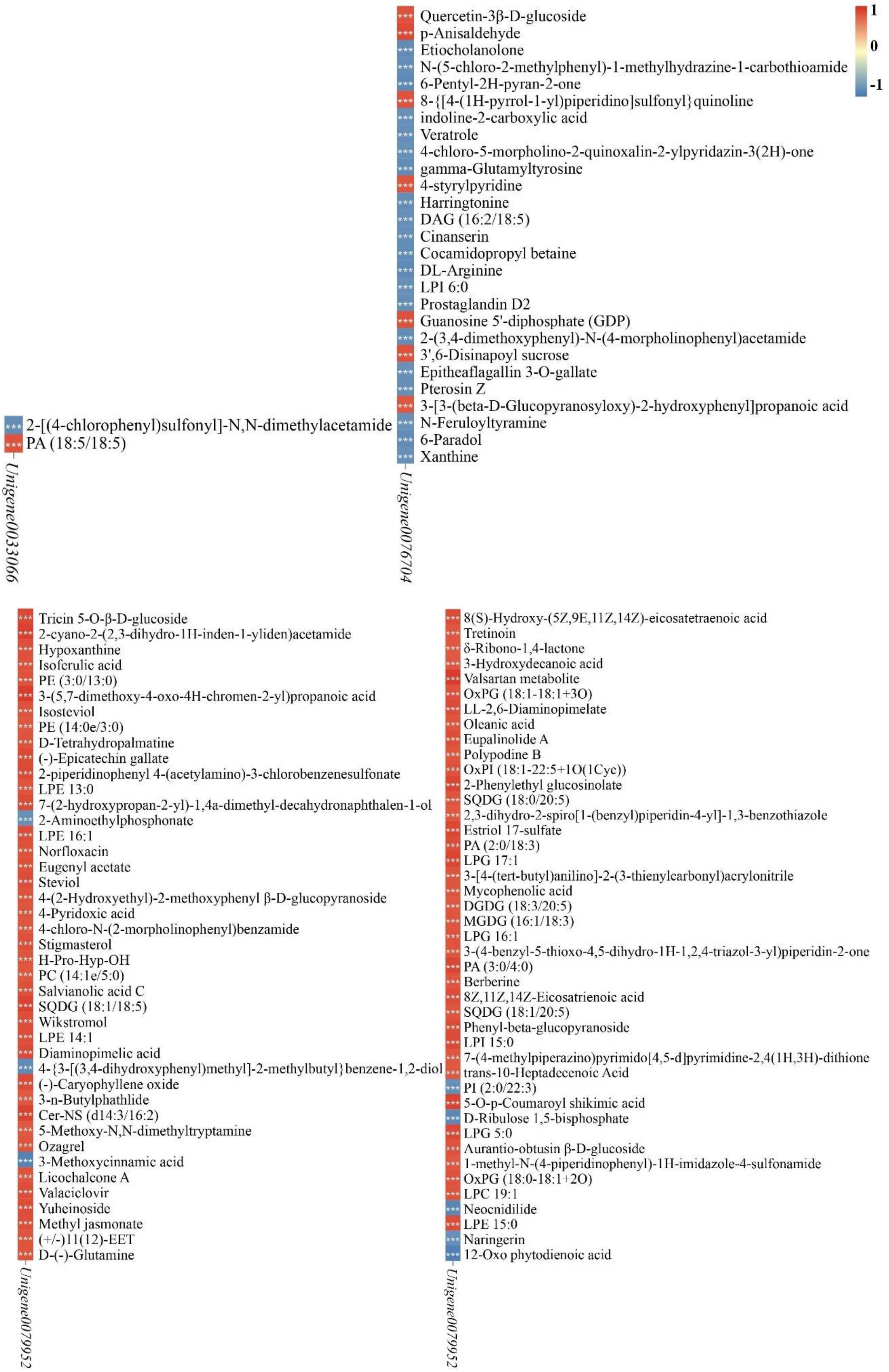
1
0
-1
Quercetin-3β-D-glucoside
p-Anisaldehyde
Etiocholanolone
N-(5-chloro-2-methylphenyl)-1-methylhydrazine-1-carbothioamide
6-Pentyl-2H-pyran-2-one
8-{[4-(1H-pyrrol-1-yl)piperidino]sulfonyl}quinoline
indoline-2-carboxylic acid
Veratrole
4-chloro-5-morpholino-2-quinoxalin-2-ylpyridazin-3(2H)-one
gamma-Glutamyltyrosine
4-styrylpyridine
Harringtonine
DAG (16:2/18:5)
Cinanserin
Cocamidopropyl betaine
DL-Arginine
LPI 6:0
Prostaglandin D2
Guanosine 5'-diphosphate (GDP)
2-(3,4-dimethoxyphenyl)-N-(4-morpholinophenyl)acetamide
3',6-Disinapoyl sucrose
Epitheaflagallin 3-O-gallate
Pterosin Z
3-[3-(beta-D-Glucopyranosyloxy)-2-hydroxyphenyl]propanoic acid
N-Feruloyltyramine
6-Paradol
Xanthine
Unigene0076704
2-[(4-chlorophenyl)sulfonyl]-N,N-dimethylacetamide
PA (18:5/18:5)
Unigene0033066
Tricin 5-O-β-D-glucoside
2-cyano-2-(2,3-dihydro-1H-inden-1-yliden)acetamide
Hypoxanthine
Isoferulic acid
PE (3:0/13:0)
3-(5,7-dimethoxy-4-oxo-4H-chromen-2-yl)propanoic acid
Isosteviol
PE (14:0e/3:0)
D-Tetrahydropalmatine
(-)-Epicatechin gallate
2-piperidinophenyl 4-(acetylamino)-3-chlorobenzenesulfonate
LPE 13:0
7-(2-hydroxypropan-2-yl)-1,4a-dimethyl-decahydronaphthalen-1-ol
2-Aminoethylphosphonate
LPE 16:1
Norfloxacin
Eugenyl acetate
Steviol
4-(2-Hydroxyethyl)-2-methoxyphenyl β-D-glucopyranoside
4-Pyridoxic acid
4-chloro-N-(2-morpholinophenyl)benzamide
Stigmasterol
H-Pro-Hyp-OH
PC (14:1e/5:0)
Salvianolic acid C
SQDG (18:1/18:5)
Wikstromol
LPE 14:1
Diaminopimelic acid
4-{3-[(3,4-dihydroxyphenyl)methyl]-2-methylbutyl}benzene-1,2-diol
(-)-Caryophyllene oxide
3-n-Butylphathlide
Cer-NS (d14:3/16:2)
5-Methoxy-N,N-dimethyltryptamine
Ozagrel
3-Methoxycinnamic acid
Licochalcone A
Valaciclovir
Yuheinoside
Methyl jasmonate
(+/-)11(12)-EET
D-(-)-Glutamine
Unigene0079952
8(S)-Hydroxy-(5Z,9E,11Z,14Z)-eicosatetraenoic acid
Tretinoin
δ-Ribono-1,4-lactone
3-Hydroxydecanoic acid
Valsartan metabolite
OxPG (18:1-18:1+3O)
LL-2,6-Diaminopimelate
Oleanic acid
Eupalinolide A
Polypodine B
OxPI (18:1-22:5+1O(1Cyc))
2-Phenylethyl glucosinolate
SQDG (18:0/20:5)
2,3-dihydro-2-spiro[1-(benzyl)piperidin-4-yl]-1,3-benzothiazole
Estriol 17-sulfate
PA (2:0/18:3)
LPG 17:1
3-[4-(tert-butyl)anilino]-2-(3-thienylcarbonyl)acrylonitrile
Mycophenolic acid
DGDG (18:3/20:5)
MGDG (16:1/18:3)
LPG 16:1
3-(4-benzyl-5-thioxo-4,5-dihydro-1H-1,2,4-triazol-3-yl)piperidin-2-one
PA (3:0/4:0)
Berberine
8Z,11Z,14Z-Eicosatrienoic acid
SQDG (18:1/20:5)
Phenyl-beta-glucopyranoside
LPI 15:0
7-(4-methylpiperazino)pyrimido[4,5-d]pyrimidine-2,4(1H,3H)-dithione
trans-10-Heptadecenoic Acid
PI (2:0/22:3)
5-O-p-Coumaroyl shikimic acid
D-Ribulose 1,5-bisphosphate
LPG 5:0
Aurantio-obtusin β-D-glucoside
1-methyl-N-(4-piperidinophenyl)-1H-imidazole-4-sulfonamide
OxPG (18:0-18:1+2O)
LPC 19:1
Neocnidilide
LPE 15:0
Naringerin
12-Oxo phytodienoic acid
Unigene0079952

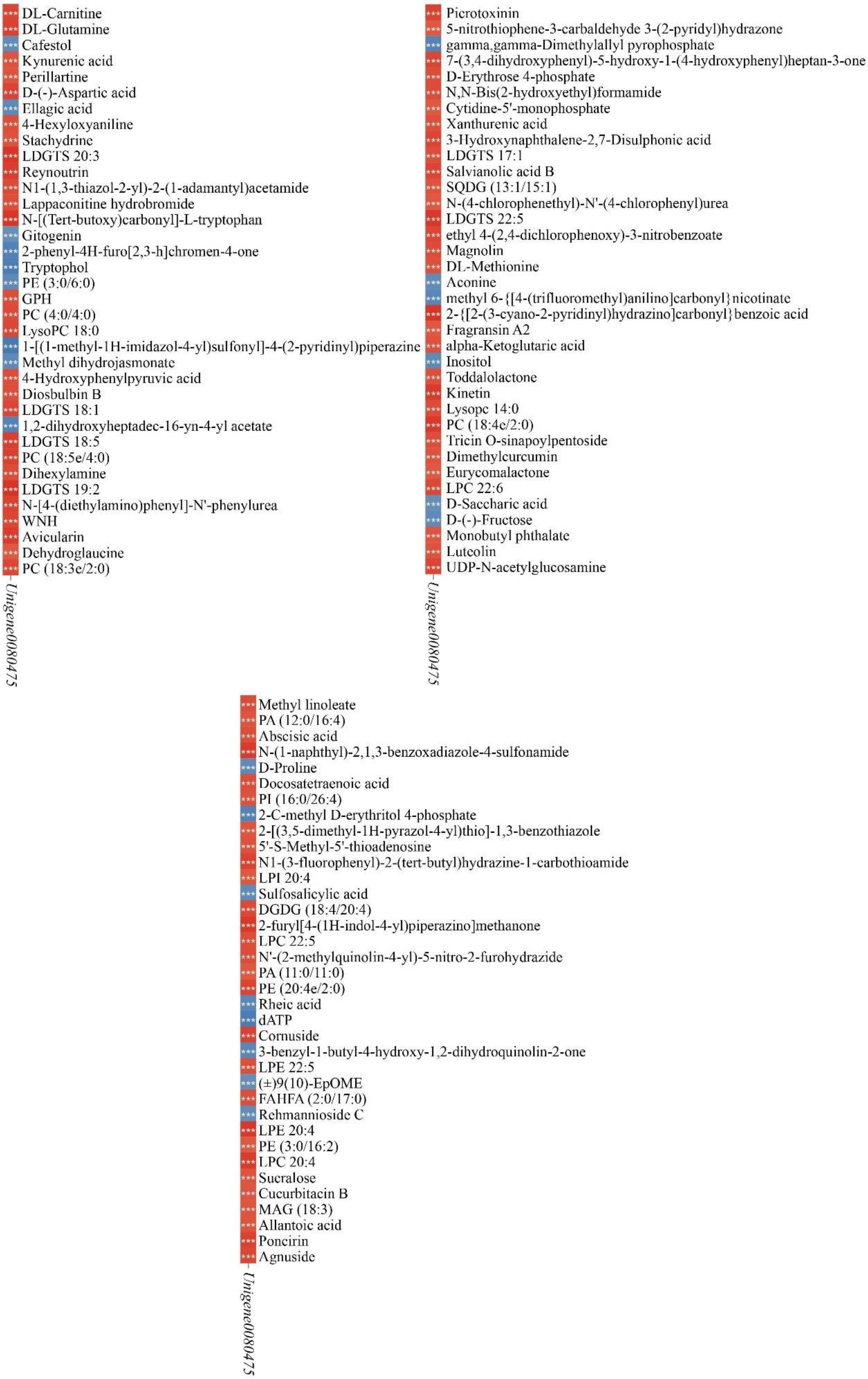

Supplementary Figure S4. Heatmap of correlations between major DGEs and metabolites in the TPK channel (According to the requirement of the absolute value of Person correlation coefficient |Corr|>0.8, the related DEGs and metabolome data in the TPK channel were screened and correlated. $p \geq 0.05$ is not marked; $0.01< p <0.05$ is marked as *; $0.001< p <0.01$ is marked as **; $p \leq 0.001$ is marked as ***.)
