# Supplementary material for: Transcriptome and Metabonomic Analysis of Tamarix ramosissima Potassium (K+) Channels and Transporters in Response to NaCl Stress
Source: Genes (Basel). 2022 Jul 23;13(8):1313. doi: 10.3390/genes13081313 (PMC9394374; doi:10.3390/genes13081313)
Supplement: Supplementary file 1 [file genes-13-01313-s001.zip › Supplementary Figure S6.pdf]

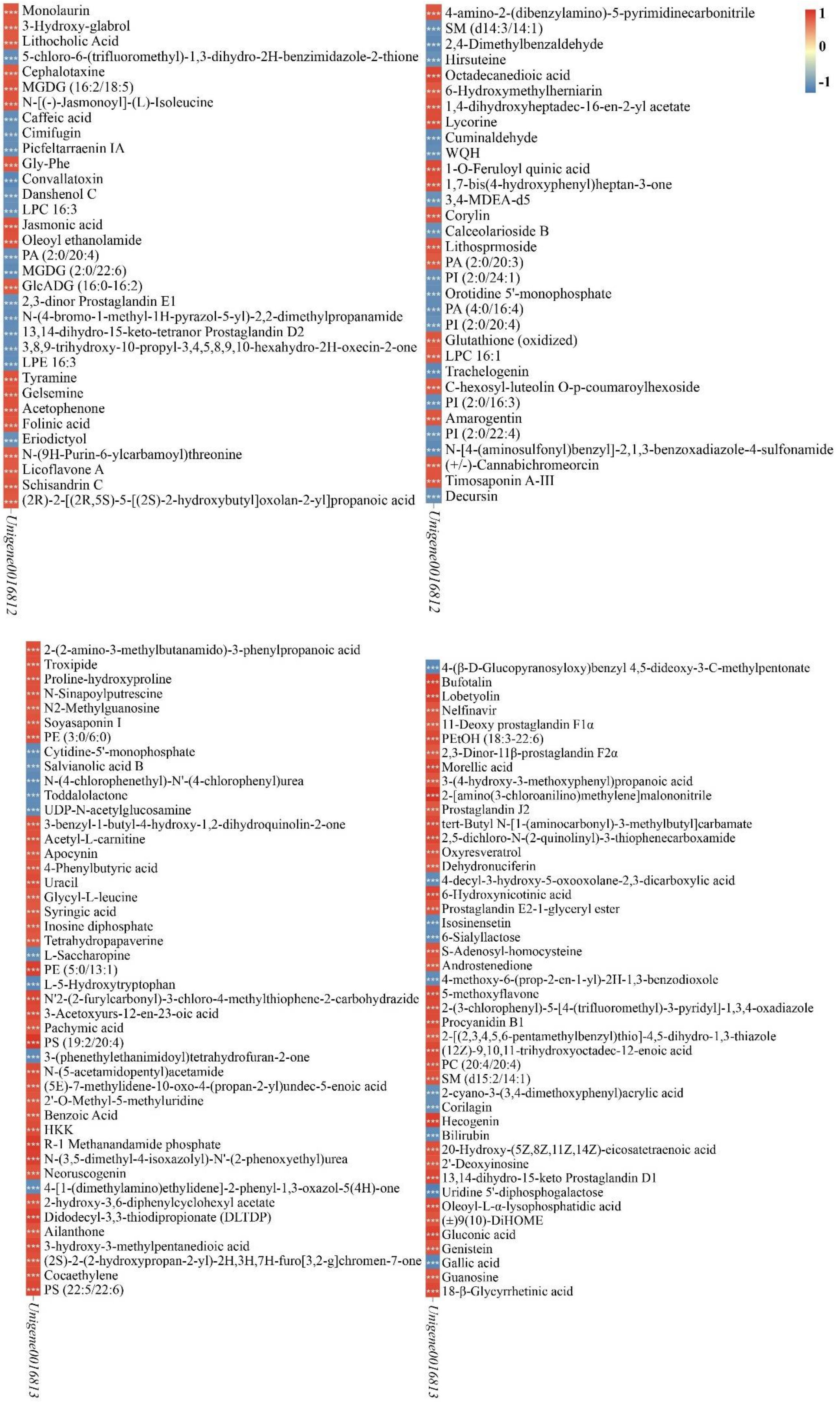
Monolaurin
3-Hydroxy-glabrol
Lithocholic Acid
5-chloro-6-(trifluoromethyl)-1,3-dihydro-2H-benzimidazole-2-thione
Cephalotaxine
MGDG (16:2/18:5)
N-[(-)-Jasmonoyl]-(L)-Isoleucine
Caffeic acid
Cimifugin
Picfeltarraenin IA
Gly-Phe
Convallatoxin
Danshenol C
LPC 16:3
Jasmonic acid
Oleoyl ethanolamide
PA (2:0/20:4)
MGDG (2:0/22:6)
GlcADG (16:0-16:2)
2,3-dinor Prostaglandin E1
N-(4-bromo-1-methyl-1H-pyrazol-5-yl)-2,2-dimethylpropanamide
13,14-dihydro-15-keto-tetranor Prostaglandin D2
3,8,9-trihydroxy-10-propyl-3,4,5,8,9,10-hexahydro-2H-oxecin-2-one
LPE 16:3
Tyramine
Gelsemine
Acetophenone
Folinic acid
Eriodictyol
N-(9H-Purin-6-ylcarbamoyl)threonine
Licoflavone A
Schisandrin C
(2R)-2-[(2R,5S)-5-[(2S)-2-hydroxybutyl]oxolan-2-yl]propanoic acid
Unigene0016812
4-amino-2-(dibenzylamino)-5-pyrimidinecarbonitrile
SM (d14:3/14:1)
2,4-Dimethylbenzaldehyde
Hirsuteine
Octadecanedioic acid
6-Hydroxymethylherniarin
1,4-dihydroxyheptadec-16-en-2-yl acetate
Lycorine
Cuminaldehyde
WQH
1-O-Feruloyl quinic acid
1,7-bis(4-hydroxyphenyl)heptan-3-one
3,4-MDEA-d5
Corylin
Calceolarioside B
Lithosprmoside
PA (2:0/20:3)
PI (2:0/24:1)
Orotidine 5'-monophosphate
PA (4:0/16:4)
PI (2:0/20:4)
Glutathione (oxidized)
LPC 16:1
Trachelogenin
C-hexosyl-luteolin O-p-coumaroylhexoside
PI (2:0/16:3)
Amarogentin
PI (2:0/22:4)
N-[4-(aminosulfonyl)benzyl]-2,1,3-benzoxadiazole-4-sulfonamide
(+/-)-Cannabichromeorcin
Timosaponin A-III
Decursin
Unigene0016812
1
0
-1
2-(2-amino-3-methylbutanamido)-3-phenylpropanoic acid
Troxipide
Proline-hydroxyproline
N-Sinapoylputrescine
N2-Methylguanosine
Soyasaponin I
PE (3:0/6:0)
Cytidine-5'-monophosphate
Salvianolic acid B
N-(4-chlorophenethyl)-N'-(4-chlorophenyl)urea
Toddalolactone
UDP-N-acetylglucosamine
3-benzyl-1-butyl-4-hydroxy-1,2-dihydroquinolin-2-one
Acetyl-L-carnitine
Apocynin
4-Phenylbutyric acid
Uracil
Glycyl-L-leucine
Syringic acid
Inosine diphosphate
Tetrahydropapaverine
L-Saccharopine
PE (5:0/13:1)
L-5-Hydroxytryptophan
N'2-(2-furylcarbonyl)-3-chloro-4-methylthiophene-2-carbohydrazide
3-Acetoxyurs-12-en-23-oic acid
Pachymic acid
PS (19:2/20:4)
3-(phenethylethanimidoyl)tetrahydrofuran-2-one
N-(5-acetamidopentyl)acetamide
(5E)-7-methylidene-10-oxo-4-(propan-2-yl)undec-5-enoic acid
2'-O-Methyl-5-methyluridine
Benzoic Acid
HKK
R-1 Methanandamide phosphate
N-(3,5-dimethyl-4-isoxazolyl)-N'-(2-phenoxyethyl)urea
Neoruscogenin
4-[1-(dimethylamino)ethylidene]-2-phenyl-1,3-oxazol-5(4H)-one
2-hydroxy-3,6-diphenylcyclohexyl acetate
Didodecyl-3,3-thiodipropionate (DLTDP)
Ailanthone
3-hydroxy-3-methylpentanedioic acid
(2S)-2-(2-hydroxypropan-2-yl)-2H,3H,7H-furo[3,2-g]chromen-7-one
Cocaethylene
PS (22:5/22:6)
Unigene0016813
4-(β-D-Glucopyranosyloxy)benzyl 4,5-dideoxy-3-C-methylpentonate
Bufotalin
Lobetyolin
Nelfinavir
11-Deoxy prostaglandin F1α
PEtOH (18:3-22:6)
2,3-Dinor-11β-prostaglandin F2α
Morellic acid
3-(4-hydroxy-3-methoxyphenyl)propanoic acid
2-[amino(3-chloroanilino)methylene]malononitrile
Prostaglandin J2
tert-Butyl N-[1-(aminocarbonyl)-3-methylbutyl]carbamate
2,5-dichloro-N-(2-quinolinyl)-3-thiophenecarboxamide
Oxyresveratrol
Dehydronuciferin
4-decyl-3-hydroxy-5-oxooxolane-2,3-dicarboxylic acid
6-Hydroxynicotinic acid
Prostaglandin E2-1-glyceryl ester
Isosinensetin
6-Sialyllactose
S-Adenosyl-homocysteine
Androstenedione
4-methoxy-6-(prop-2-en-1-yl)-2H-1,3-benzodioxole
5-methoxyflavone
2-(3-chlorophenyl)-5-[4-(trifluoromethyl)-3-pyridyl]-1,3,4-oxadiazole
Procyanidin B1
2-[(2,3,4,5,6-pentamethylbenzyl)thio]-4,5-dihydro-1,3-thiazole
(12Z)-9,10,11-trihydroxyoctadec-12-enoic acid
PC (20:4/20:4)
SM (d15:2/14:1)
2-cyano-3-(3,4-dimethoxyphenyl)acrylic acid
Corilagin
Hecogenin
Bilirubin
20-Hydroxy-(5Z,8Z,11Z,14Z)-eicosatetraenoic acid
2'-Deoxyinosine
13,14-dihydro-15-keto Prostaglandin D1
Uridine 5'-diphosphogalactose
Oleoyl-L-α-lysophosphatidic acid
(±)9(10)-DiHOME
Gluconic acid
Genistein
Gallic acid
Guanosine
18-β-Glycyrrhetinic acid
Unigene0016813

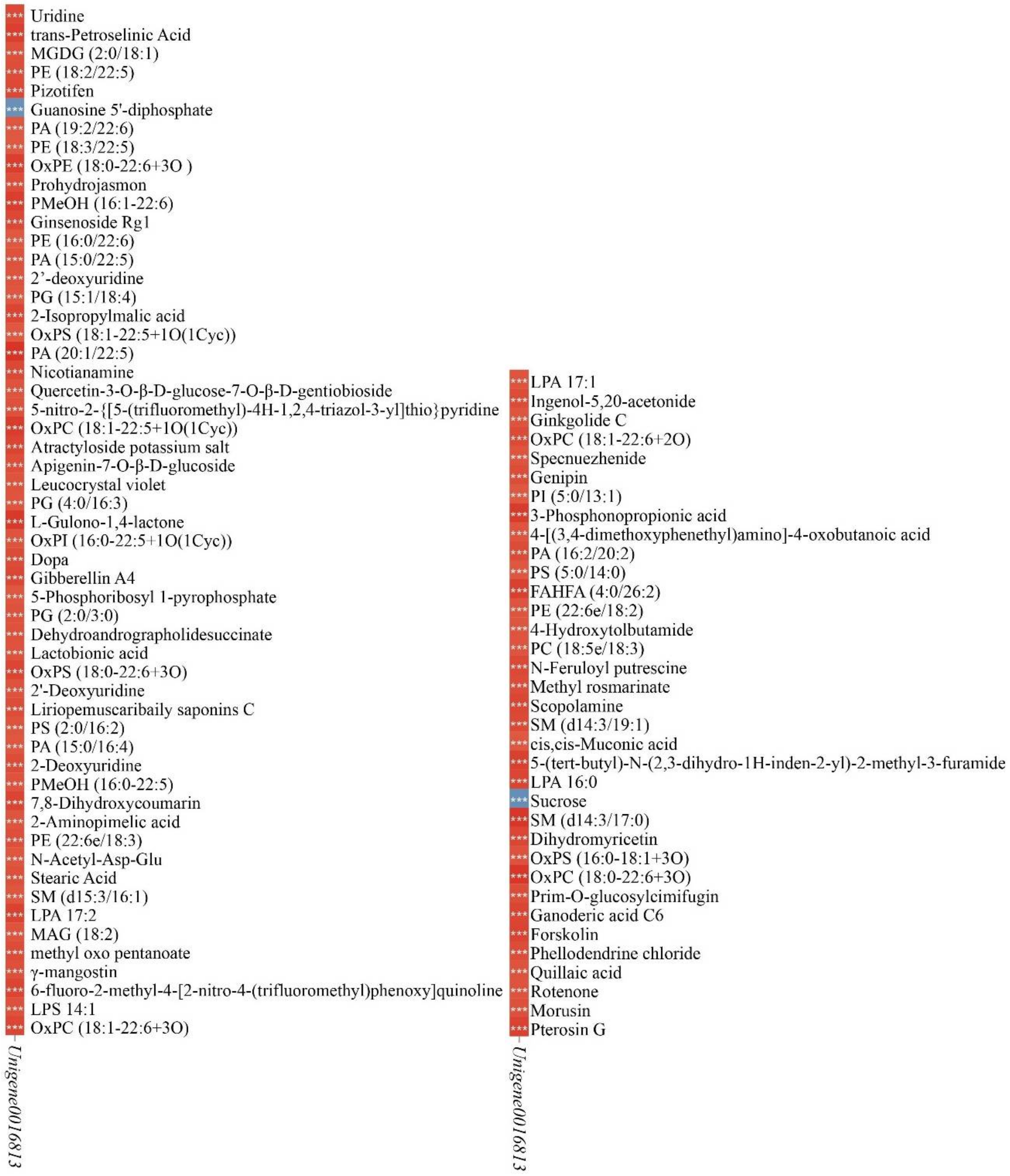
Uridine
trans-Petroselinic Acid
MGDG (2:0/18:1)
PE (18:2/22:5)
Pizotifen
Guanosine 5'-diphosphate
PA (19:2/22:6)
PE (18:3/22:5)
OxPE (18:0-22:6+3O)
Prohydrojasmon
PMeOH (16:1-22:6)
Ginsenoside Rg1
PE (16:0/22:6)
PA (15:0/22:5)
2'-deoxyuridine
PG (15:1/18:4)
2-Isopropylmalic acid
OxPS (18:1-22:5+1O(1Cyc))
PA (20:1/22:5)
Nicotianamine
Quercetin-3-O-β-D-glucose-7-O-β-D-gentiobioside
5-nitro-2-{[5-(trifluoromethyl)-4H-1,2,4-triazol-3-yl]thio}pyridine
OxPC (18:1-22:5+1O(1Cyc))
Atractyloside potassium salt
Apigenin-7-O-β-D-glucoside
Leucocrystal violet
PG (4:0/16:3)
L-Gulono-1,4-lactone
OxPI (16:0-22:5+1O(1Cyc))
Dopa
Gibberellin A4
5-Phosphoribosyl 1-pyrophosphate
PG (2:0/3:0)
Dehydroandrographolidesuccinate
Lactobionic acid
OxPS (18:0-22:6+3O)
2'-Deoxyuridine
Liriopemuscaribaily saponins C
PS (2:0/16:2)
PA (15:0/16:4)
2-Deoxyuridine
PMeOH (16:0-22:5)
7,8-Dihydroxycoumarin
2-Aminopimelic acid
PE (22:6e/18:3)
N-Acetyl-Asp-Glu
Stearic Acid
SM (d15:3/16:1)
LPA 17:2
MAG (18:2)
methyl oxo pentanoate
γ-mangostin
6-fluoro-2-methyl-4-[2-nitro-4-(trifluoromethyl)phenoxy]quinoline
LPS 14:1
OxPC (18:1-22:6+3O)
Unigene0016813
LPA 17:1
Ingenol-5,20-acetonide
Ginkgolide C
OxPC (18:1-22:6+2O)
Specnuezhenide
Genipin
PI (5:0/13:1)
3-Phosphonopropionic acid
4-[(3,4-dimethoxyphenethyl)amino]-4-oxobutanoic acid
PA (16:2/20:2)
PS (5:0/14:0)
FAHFA (4:0/26:2)
PE (22:6e/18:2)
4-Hydroxytolbutamide
PC (18:5e/18:3)
N-Feruloyl putrescine
Methyl rosmarinate
Scopolamine
SM (d14:3/19:1)
cis,cis-Muconic acid
5-(tert-butyl)-N-(2,3-dihydro-1H-inden-2-yl)-2-methyl-3-furamide
LPA 16:0
Sucrose
SM (d14:3/17:0)
Dihydromyricetin
OxPS (16:0-18:1+3O)
OxPC (18:0-22:6+3O)
Prim-O-glucosylcimifugin
Ganoderic acid C6
Forskolin
Phellodendrine chloride
Quillaic acid
Rotenone
Morusin
Pterosin G
Unigene0016813

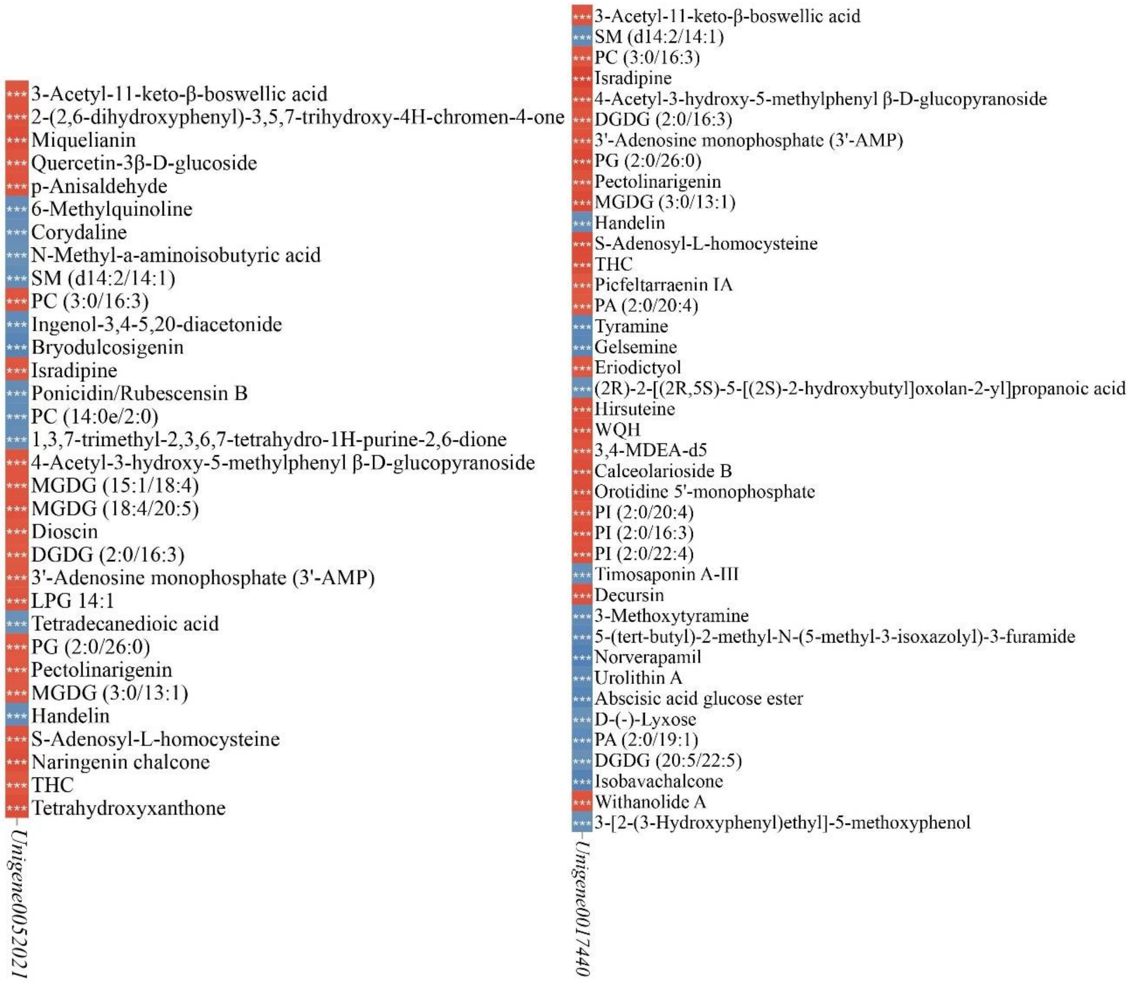
3-Acetyl-11-keto-β-boswellic acid
2-(2,6-dihydroxyphenyl)-3,5,7-trihydroxy-4H-chromen-4-one
Miquelianin
Quercetin-3β-D-glucoside
p-Anisaldehyde
6-Methylquinoline
Corydaline
N-Methyl-a-aminoisobutyric acid
SM (d14:2/14:1)
PC (3:0/16:3)
Ingenol-3,4-5,20-diacetonide
Bryodulcosigenin
Isradipine
Ponicidin/Rubescensin B
PC (14:0e/2:0)
1,3,7-trimethyl-2,3,6,7-tetrahydro-1H-purine-2,6-dione
4-Acetyl-3-hydroxy-5-methylphenyl β-D-glucopyranoside
MGDG (15:1/18:4)
MGDG (18:4/20:5)
Dioscin
DGDG (2:0/16:3)
3'-Adenosine monophosphate (3'-AMP)
LPG 14:1
Tetradecanedioic acid
PG (2:0/26:0)
Pectolinarigenin
MGDG (3:0/13:1)
Handelin
S-Adenosyl-L-homocysteine
Naringenin chalcone
THC
Tetrahydroxyxanthone
Unigene0052021
3-Acetyl-11-keto-β-boswellic acid
SM (d14:2/14:1)
PC (3:0/16:3)
Isradipine
4-Acetyl-3-hydroxy-5-methylphenyl β-D-glucopyranoside
DGDG (2:0/16:3)
3'-Adenosine monophosphate (3'-AMP)
PG (2:0/26:0)
Pectolinarigenin
MGDG (3:0/13:1)
Handelin
S-Adenosyl-L-homocysteine
THC
Picfeltarraenin IA
PA (2:0/20:4)
Tyramine
Gelsemine
Eriodictyol
(2R)-2-[(2R,5S)-5-[(2S)-2-hydroxybutyl]oxolan-2-yl]propanoic acid
Hirsuteine
WQH
3,4-MDEA-d5
Calceolarioside B
Orotidine 5'-monophosphate
PI (2:0/20:4)
PI (2:0/16:3)
PI (2:0/22:4)
Timosaponin A-III
Decursin
3-Methoxytyramine
5-(tert-butyl)-2-methyl-N-(5-methyl-3-isoxazolyl)-3-furamide
Norverapamil
Urolithin A
Abscisic acid glucose ester
D-(-)-Lyxose
PA (2:0/19:1)
DGDG (20:5/22:5)
Isobavachalcone
Withanolide A
3-[2-(3-Hydroxyphenyl)ethyl]-5-methoxyphenol
Unigene0017440

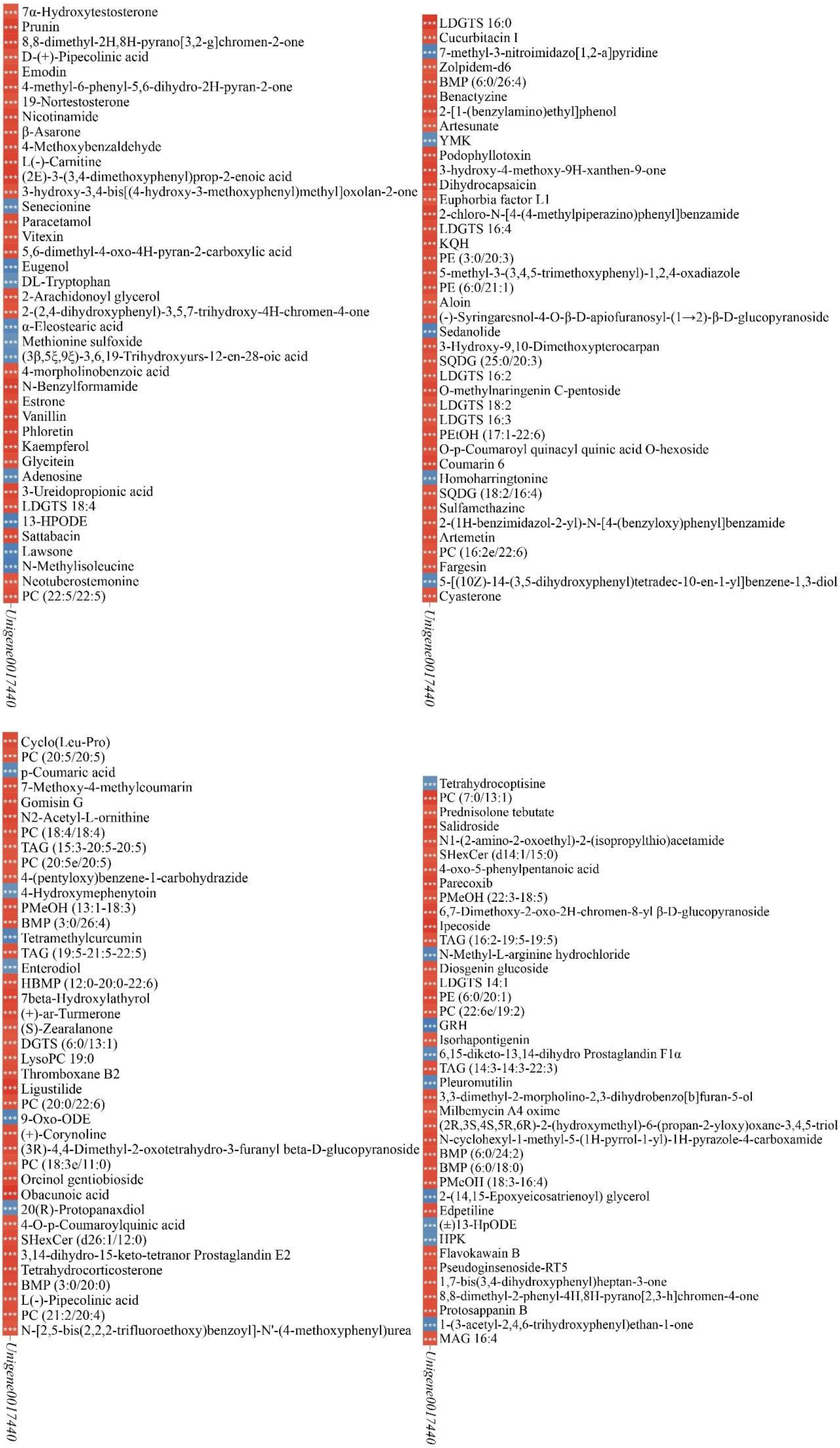
7α-Hydroxytestosterone
Prunin
8,8-dimethyl-2H,8H-pyrano[3,2-g]chromen-2-one
D-(+)-Pipecolinic acid
Emodin
4-methyl-6-phenyl-5,6-dihydro-2H-pyran-2-one
19-Nortestosterone
Nicotinamide
β-Asarone
4-Methoxybenzaldehyde
L(-)-Carnitine
(2E)-3-(3,4-dimethoxyphenyl)prop-2-enoic acid
3-hydroxy-3,4-bis[(4-hydroxy-3-methoxyphenyl)methyl]oxolan-2-one
Senecionine
Paracetamol
Vitexin
5,6-dimethyl-4-oxo-4H-pyran-2-carboxylic acid
Eugenol
DL-Tryptophan
2-Arachidonoyl glycerol
2-(2,4-dihydroxyphenyl)-3,5,7-trihydroxy-4H-chromen-4-one
α-Eleostearic acid
Methionine sulfoxide
(3β,5ξ,9ξ)-3,6,19-Trihydroxyurs-12-en-28-oic acid
4-morpholinobenzoic acid
N-Benzylformamide
Estrone
Vanillin
Phloretin
Kaempferol
Glycitein
Adenosine
3-Ureidopropionic acid
LDGTS 18:4
13-HPODE
Sattabacin
Lawsone
N-Methylisoleucine
Neotuberostemonine
PC (22:5/22:5)
Unigene0017440
LDGTS 16:0
Cucurbitacin I
7-methyl-3-nitroimidazo[1,2-a]pyridine
Zolpidem-d6
BMP (6:0/26:4)
Benactyzine
2-[1-(benzylamino)ethyl]phenol
Artesunate
YMK
Podophyllotoxin
3-hydroxy-4-methoxy-9H-xanthen-9-one
Dihydrocapsaicin
Euphorbia factor L1
2-chloro-N-[4-(4-methylpiperazino)phenyl]benzamide
LDGTS 16:4
KQH
PE (3:0/20:3)
5-methyl-3-(3,4,5-trimethoxyphenyl)-1,2,4-oxadiazole
PE (6:0/21:1)
Aloin
(-)-Syringaresnol-4-O-β-D-apiofuranosyl-(1→2)-β-D-glucopyranoside
Sedanolide
3-Hydroxy-9,10-Dimethoxypterocarpan
SQDG (25:0/20:3)
LDGTS 16:2
O-methylnaringenin C-pentoside
LDGTS 18:2
LDGTS 16:3
PEtOH (17:1-22:6)
O-p-Coumaroyl quinacyl quinic acid O-hexoside
Coumarin 6
Homoharringtonine
SQDG (18:2/16:4)
Sulfamethazine
2-(1H-benzimidazol-2-yl)-N-[4-(benzyloxy)phenyl]benzamide
Artemetin
PC (16:2e/22:6)
Fargesin
5-[(10Z)-14-(3,5-dihydroxyphenyl)tetradec-10-en-1-yl]benzene-1,3-diol
Cyasterone
Unigene0017440
Cyclo(Leu-Pro)
PC (20:5/20:5)
p-Coumaric acid
7-Methoxy-4-methylcoumarin
Gomisin G
N2-Acetyl-L-ornithine
PC (18:4/18:4)
TAG (15:3-20:5-20:5)
PC (20:5e/20:5)
4-(pentyloxy)benzene-1-carbohydrazide
4-Hydroxymephenytoin
PMeOH (13:1-18:3)
BMP (3:0/26:4)
Tetramethylcurcumin
TAG (19:5-21:5-22:5)
Enterodiol
HBMP (12:0-20:0-22:6)
7beta-Hydroxylathyrol
(+)-ar-Turmerone
(S)-Zearalanone
DGTS (6:0/13:1)
LysoPC 19:0
Thromboxane B2
Ligustilide
PC (20:0/22:6)
9-Oxo-ODE
(+)-Corynoline
(3R)-4,4-Dimethyl-2-oxotetrahydro-3-furanyl beta-D-glucopyranoside
PC (18:3e/11:0)
Orcinol gentiobioside
Obacunoic acid
20(R)-Protopanaxdiol
4-O-p-Coumaroylquinic acid
SHexCer (d26:1/12:0)
3,14-dihydro-15-keto-tetranor Prostaglandin E2
Tetrahydrocorticosterone
BMP (3:0/20:0)
L(-)-Pipecolinic acid
PC (21:2/20:4)
N-[2,5-bis(2,2,2-trifluoroethoxy)benzoyl]-N'-(4-methoxyphenyl)urea
Unigene0017440
Tetrahydrocoptisine
PC (7:0/13:1)
Prednisolone tebutate
Salidroside
N1-(2-amino-2-oxoethyl)-2-(isopropylthio)acetamide
SHexCer (d14:1/15:0)
4-oxo-5-phenylpentanoic acid
Parecoxib
PMeOH (22:3-18:5)
6,7-Dimethoxy-2-oxo-2H-chromen-8-yl β-D-glucopyranoside
Ipecoside
TAG (16:2-19:5-19:5)
N-Methyl-L-arginine hydrochloride
Diosgenin glucoside
LDGTS 14:1
PE (6:0/20:1)
PC (22:6e/19:2)
GRH
Isorhapontigenin
6,15-diketo-13,14-dihydro Prostaglandin F1α
TAG (14:3-14:3-22:3)
Pleuromutilin
3,3-dimethyl-2-morpholino-2,3-dihydrobenzo[b]furan-5-ol
Milbemycin A4 oxime
(2R,3S,4S,5R,6R)-2-(hydroxymethyl)-6-(propan-2-yloxy)oxane-3,4,5-triol
N-cyclohexyl-1-methyl-5-(1H-pyrrol-1-yl)-1H-pyrazole-4-carboxamide
BMP (6:0/24:2)
BMP (6:0/18:0)
PMeOH (18:3-16:4)
2-(14,15-Epoxyeicosatrienoyl) glycerol
Edpetiline
(±)13-HpODE
HPK
Flavokawain B
Pseudoginsenoside-RT5
1,7-bis(3,4-dihydroxyphenyl)heptan-3-one
8,8-dimethyl-2-phenyl-4H,8H-pyrano[2,3-h]chromen-4-one
Protosappanin B
1-(3-acetyl-2,4,6-trihydroxyphenyl)ethan-1-one
MAG 16:4
Unigene0017440

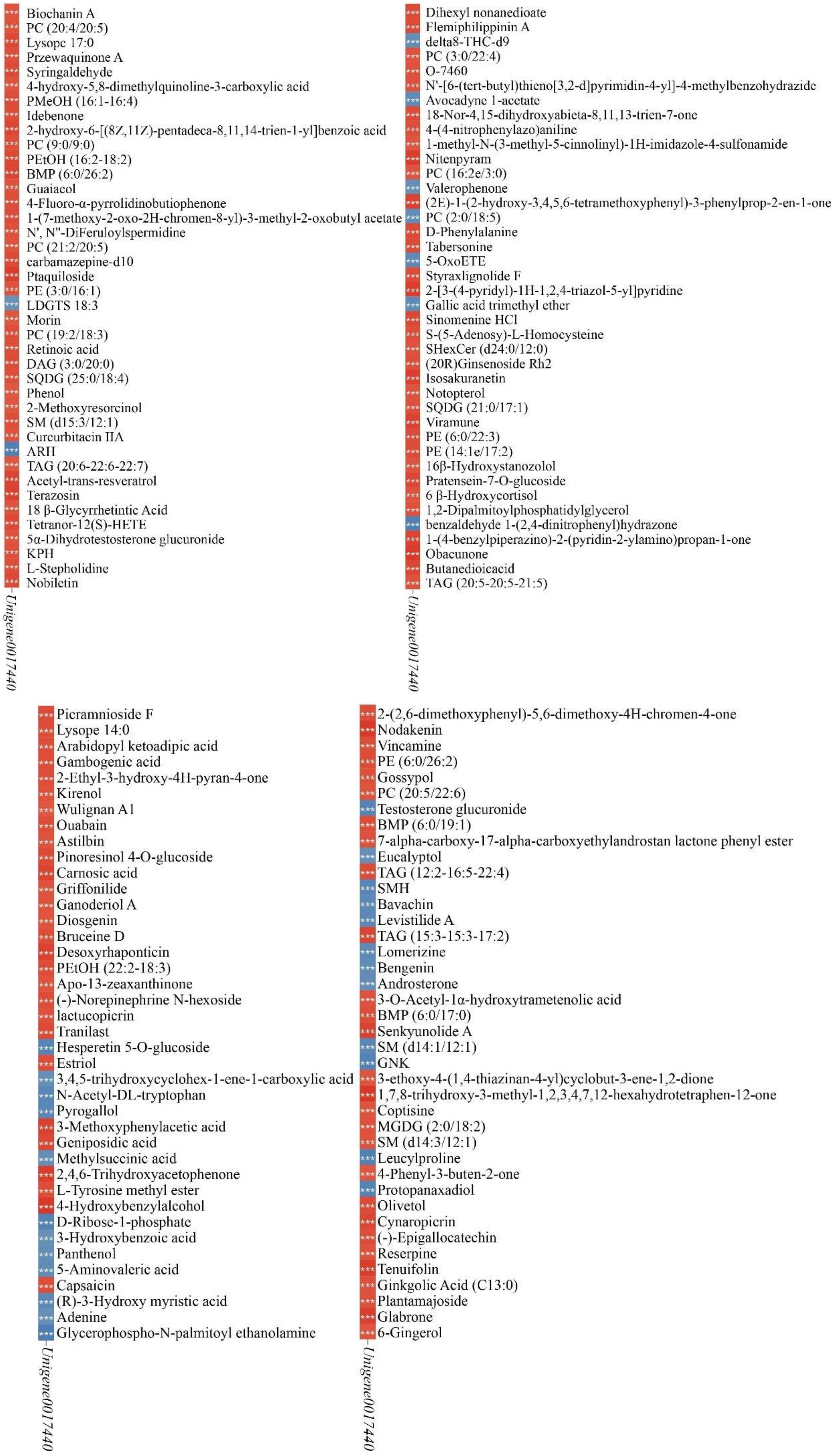
Biochanin A
PC (20:4/20:5)
Lysopc 17:0
Przewaquinone A
Syringaldehyde
4-hydroxy-5,8-dimethylquinoline-3-carboxylic acid
PMeOH (16:1-16:4)
Idebenone
2-hydroxy-6-[(8Z,11Z)-pentadeca-8,11,14-trien-1-yl]benzoic acid
PC (9:0/9:0)
PEtOH (16:2-18:2)
BMP (6:0/26:2)
Guaiacol
4-Fluoro-α-pyrrolidinobutiophenone
1-(7-methoxy-2-oxo-2H-chromen-8-yl)-3-methyl-2-oxobutyl acetate
N', N''-DiFeruloylspermidine
PC (21:2/20:5)
carbamazepine-d10
Ptaquiloside
PE (3:0/16:1)
LDGTS 18:3
Morin
PC (19:2/18:3)
Retinoic acid
DAG (3:0/20:0)
SQDG (25:0/18:4)
Phenol
2-Methoxyresorcinol
SM (d15:3/12:1)
Curcurbitacin IIA
ARII
TAG (20:6-22:6-22:7)
Acetyl-trans-resveratrol
Terazosin
18 β-Glycyrrhetintic Acid
Tetranor-12(S)-HETE
5α-Dihydrotestosterone glucuronide
KPH
L-Stepholidine
Nobiletin
Unigene0017440
Dihexyl nonanedioate
Flemiphilippinin A
delta8-THC-d9
PC (3:0/22:4)
O-7460
N'-[6-(tert-butyl)thieno[3,2-d]pyrimidin-4-yl]-4-methylbenzohydrazide
Avocadyne 1-acetate
18-Nor-4,15-dihydroxyabieta-8,11,13-trien-7-one
4-(4-nitrophenylazo)aniline
1-methyl-N-(3-methyl-5-cinnolinyl)-1H-imidazole-4-sulfonamide
Nitenpyram
PC (16:2e/3:0)
Valerophenone
(2E)-1-(2-hydroxy-3,4,5,6-tetramethoxyphenyl)-3-phenylprop-2-en-1-one
PC (2:0/18:5)
D-Phenylalanine
Tabersonine
5-OxoETE
Styraxlignolide F
2-[3-(4-pyridyl)-1H-1,2,4-triazol-5-yl]pyridine
Gallic acid trimethyl ether
Sinomenine HCl
S-(5-Adenosy)-L-Homocysteine
SHexCer (d24:0/12:0)
(20R)Ginsenoside Rh2
Isosakuranetin
Notopterol
SQDG (21:0/17:1)
Viramune
PE (6:0/22:3)
PE (14:1e/17:2)
16β-Hydroxystanozolol
Pratensein-7-O-glucoside
6 β-Hydroxycortisol
1,2-Dipalmitoylphosphatidylglycerol
benzaldehyde 1-(2,4-dinitrophenyl)hydrazone
1-(4-benzylpiperazino)-2-(pyridin-2-ylamino)propan-1-one
Obacunone
Butanedioicacid
TAG (20:5-20:5-21:5)
Unigene0017440
Picramnioside F
Lysope 14:0
Arabidopyl ketoadipic acid
Gambogenic acid
2-Ethyl-3-hydroxy-4H-pyran-4-one
Kirenol
Wulignan A1
Ouabain
Astilbin
Pinoresinol 4-O-glucoside
Carnosic acid
Griffonilide
Ganoderiol A
Diosgenin
Bruceine D
Desoxyrhaponticin
PEtOH (22:2-18:3)
Apo-13-zeaxanthinone
(-)-Norepinephrine N-hexoside
lactucopicrin
Tranilast
Hesperetin 5-O-glucoside
Estriol
3,4,5-trihydroxycyclohex-1-ene-1-carboxylic acid
N-Acetyl-DL-tryptophan
Pyrogallol
3-Methoxyphenylacetic acid
Geniposidic acid
Methylsuccinic acid
2,4,6-Trihydroxyacetophenone
L-Tyrosine methyl ester
4-Hydroxybenzylalcohol
D-Ribose-1-phosphate
3-Hydroxybenzoic acid
Panthenol
5-Aminovaleric acid
Capsaicin
(R)-3-Hydroxy myristic acid
Adenine
Glycerophospho-N-palmitoyl ethanolamine
Unigene0017440
2-(2,6-dimethoxyphenyl)-5,6-dimethoxy-4H-chromen-4-one
Nodakenin
Vincamine
PE (6:0/26:2)
Gossypol
PC (20:5/22:6)
Testosterone glucuronide
BMP (6:0/19:1)
7-alpha-carboxy-17-alpha-carboxyethylandrostan lactone phenyl ester
Eucalyptol
TAG (12:2-16:5-22:4)
SMH
Bavachin
Levistilide A
TAG (15:3-15:3-17:2)
Lomerizine
Bengenin
Androsterone
3-O-Acetyl-1α-hydroxytrametenolic acid
BMP (6:0/17:0)
Senkyunolide A
SM (d14:1/12:1)
GNK
3-ethoxy-4-(1,4-thiazinan-4-yl)cyclobut-3-ene-1,2-dione
1,7,8-trihydroxy-3-methyl-1,2,3,4,7,12-hexahydrotetraphen-12-one
Coptisine
MGDG (2:0/18:2)
SM (d14:3/12:1)
Leucylproline
4-Phenyl-3-buten-2-one
Protopanaxadiol
Olivetol
Cynaropicrin
(-)-Epigallocatechin
Reserpine
Tenuifolin
Ginkgolic Acid (C13:0)
Plantamajoside
Glabrone
6-Gingerol
Unigene0017440

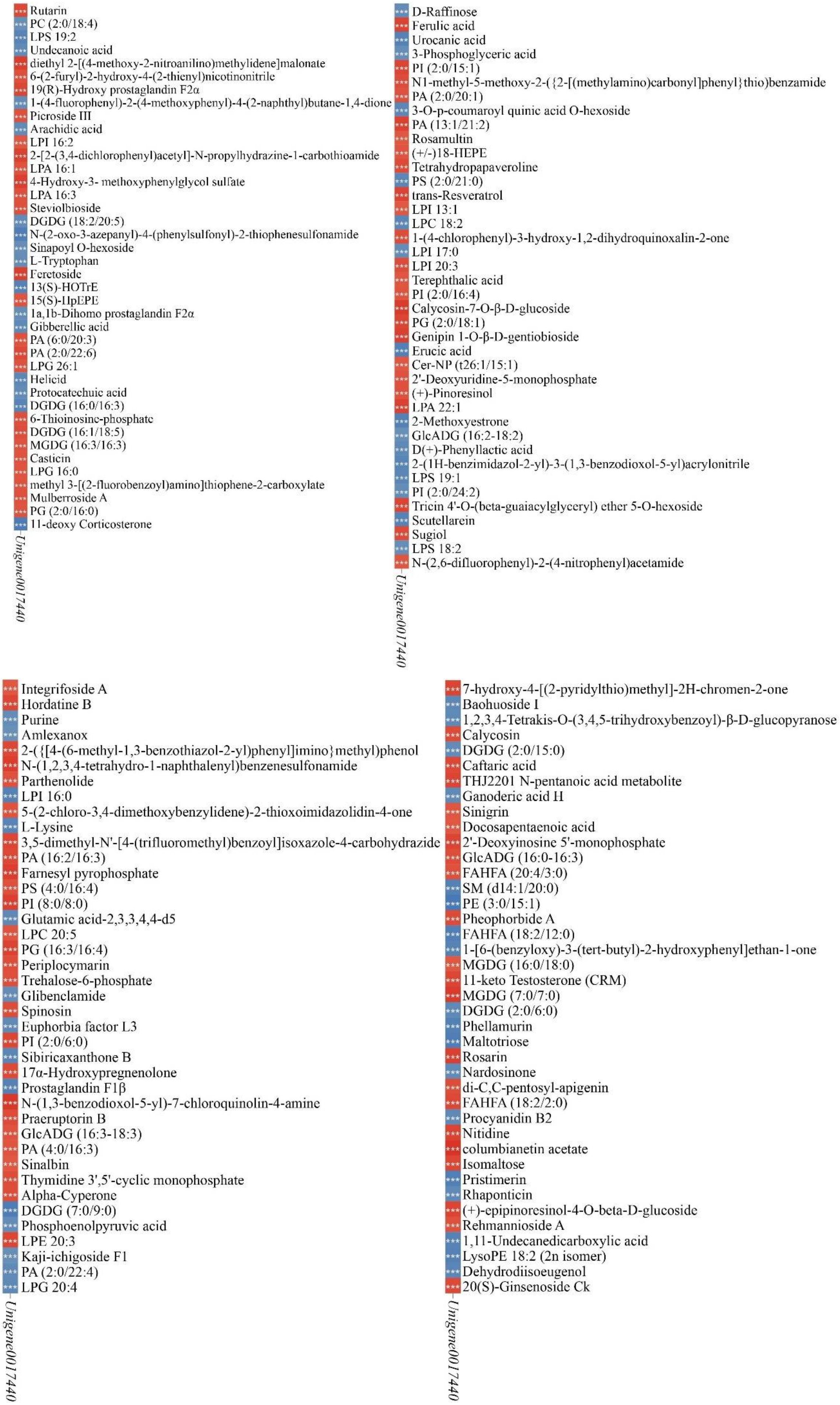
Rutarin
PC (2:0/18:4)
LPS 19:2
Undecanoic acid
diethyl 2-[(4-methoxy-2-nitroanilino)methylidene]malonate
6-(2-furyl)-2-hydroxy-4-(2-thienyl)nicotinonitrile
19(R)-Hydroxy prostaglandin F2α
1-(4-fluorophenyl)-2-(4-methoxyphenyl)-4-(2-naphthyl)butane-1,4-dione
Picroside III
Arachidic acid
LPI 16:2
2-[2-(3,4-dichlorophenyl)acetyl]-N-propylhydrazine-1-carbothioamide
LPA 16:1
4-Hydroxy-3- methoxyphenylglycol sulfate
LPA 16:3
Steviolbioside
DGDG (18:2/20:5)
N-(2-oxo-3-azepanyl)-4-(phenylsulfonyl)-2-thiophenesulfonamide
Sinapoyl O-hexoside
L-Tryptophan
Feretoside
13(S)-HOTrE
15(S)-HpEPE
1a,1b-Dihomo prostaglandin F2α
Gibberellic acid
PA (6:0/20:3)
PA (2:0/22:6)
LPG 26:1
Helicid
Protocatechuic acid
DGDG (16:0/16:3)
6-Thioinosine-phosphate
DGDG (16:1/18:5)
MGDG (16:3/16:3)
Casticin
LPG 16:0
methyl 3-[(2-fluorobenzoyl)amino]thiophene-2-carboxylate
Mulberroside A
PG (2:0/16:0)
11-deoxy Corticosterone
Unigene0017440
D-Raffinose
Ferulic acid
Urocanic acid
3-Phosphoglyceric acid
PI (2:0/15:1)
N1-methyl-5-methoxy-2-({2-[(methylamino)carbonyl]phenyl}thio)benzamide
PA (2:0/20:1)
3-O-p-coumaroyl quinic acid O-hexoside
PA (13:1/21:2)
Rosamultin
(+/-)18-HEPE
Tetrahydropapaveroline
PS (2:0/21:0)
trans-Resveratrol
LPI 13:1
LPC 18:2
1-(4-chlorophenyl)-3-hydroxy-1,2-dihydroquinoxalin-2-one
LPI 17:0
LPI 20:3
Terephthalic acid
PI (2:0/16:4)
Calycosin-7-O-β-D-glucoside
PG (2:0/18:1)
Genipin 1-O-β-D-gentiobioside
Erucic acid
Cer-NP (t26:1/15:1)
2'-Deoxyuridine-5-monophosphate
(+)-Pinoresinol
LPA 22:1
2-Methoxyestrone
GlcADG (16:2-18:2)
D(+)-Phenyllactic acid
2-(1H-benzimidazol-2-yl)-3-(1,3-benzodioxol-5-yl)acrylonitrile
LPS 19:1
PI (2:0/24:2)
Tricin 4'-O-(beta-guaiacylglyceryl) ether 5-O-hexoside
Scutellarein
Sugiol
LPS 18:2
N-(2,6-difluorophenyl)-2-(4-nitrophenyl)acetamide
Unigene0017440
Integrifoside A
Hordatine B
Purine
Amlexanox
2-({[4-(6-methyl-1,3-benzothiazol-2-yl)phenyl]imino}methyl)phenol
N-(1,2,3,4-tetrahydro-1-naphthalenyl)benzenesulfonamide
Parthenolide
LPI 16:0
5-(2-chloro-3,4-dimethoxybenzylidene)-2-thioxoimidazolidin-4-one
L-Lysine
3,5-dimethyl-N'-[4-(trifluoromethyl)benzoyl]isoxazole-4-carbohydrazide
PA (16:2/16:3)
Farnesyl pyrophosphate
PS (4:0/16:4)
PI (8:0/8:0)
Glutamic acid-2,3,3,4,4-d5
LPC 20:5
PG (16:3/16:4)
Periplocymarin
Trehalose-6-phosphate
Glibenclamide
Spinosin
Euphorbia factor L3
PI (2:0/6:0)
Sibiricaxanthone B
17α-Hydroxypregnenolone
Prostaglandin F1β
N-(1,3-benzodioxol-5-yl)-7-chloroquinolin-4-amine
Praeruptorin B
GlcADG (16:3-18:3)
PA (4:0/16:3)
Sinalbin
Thymidine 3',5'-cyclic monophosphate
Alpha-Cyperone
DGDG (7:0/9:0)
Phosphoenolpyruvic acid
LPE 20:3
Kaji-ichigoside F1
PA (2:0/22:4)
LPG 20:4
Unigene0017440
7-hydroxy-4-[(2-pyridylthio)methyl]-2H-chromen-2-one
Baohuoside I
1,2,3,4-Tetrakis-O-(3,4,5-trihydroxybenzoyl)-β-D-glucopyranose
Calycosin
DGDG (2:0/15:0)
Caftaric acid
THJ2201 N-pentanoic acid metabolite
Ganoderic acid H
Sinigrin
Docosapentaenoic acid
2'-Deoxyinosine 5'-monophosphate
GlcADG (16:0-16:3)
FAHFA (20:4/3:0)
SM (d14:1/20:0)
PE (3:0/15:1)
Pheophorbide A
FAHFA (18:2/12:0)
1-[6-(benzyloxy)-3-(tert-butyl)-2-hydroxyphenyl]ethan-1-one
MGDG (16:0/18:0)
11-keto Testosterone (CRM)
MGDG (7:0/7:0)
DGDG (2:0/6:0)
Phellamurin
Maltotriose
Rosarin
Nardosinone
di-C,C-pentosyl-apigenin
FAHFA (18:2/2:0)
Procyanidin B2
Nitidine
columbianetin acetate
Isomaltose
Pristimerin
Rhaponticin
(+)-epipinoresinol-4-O-beta-D-glucoside
Rehmannioside A
1,11-Undecanedicarboxylic acid
LysoPE 18:2 (2n isomer)
Dehydrodiisoeugenol
20(S)-Ginsenoside Ck
Unigene0017440

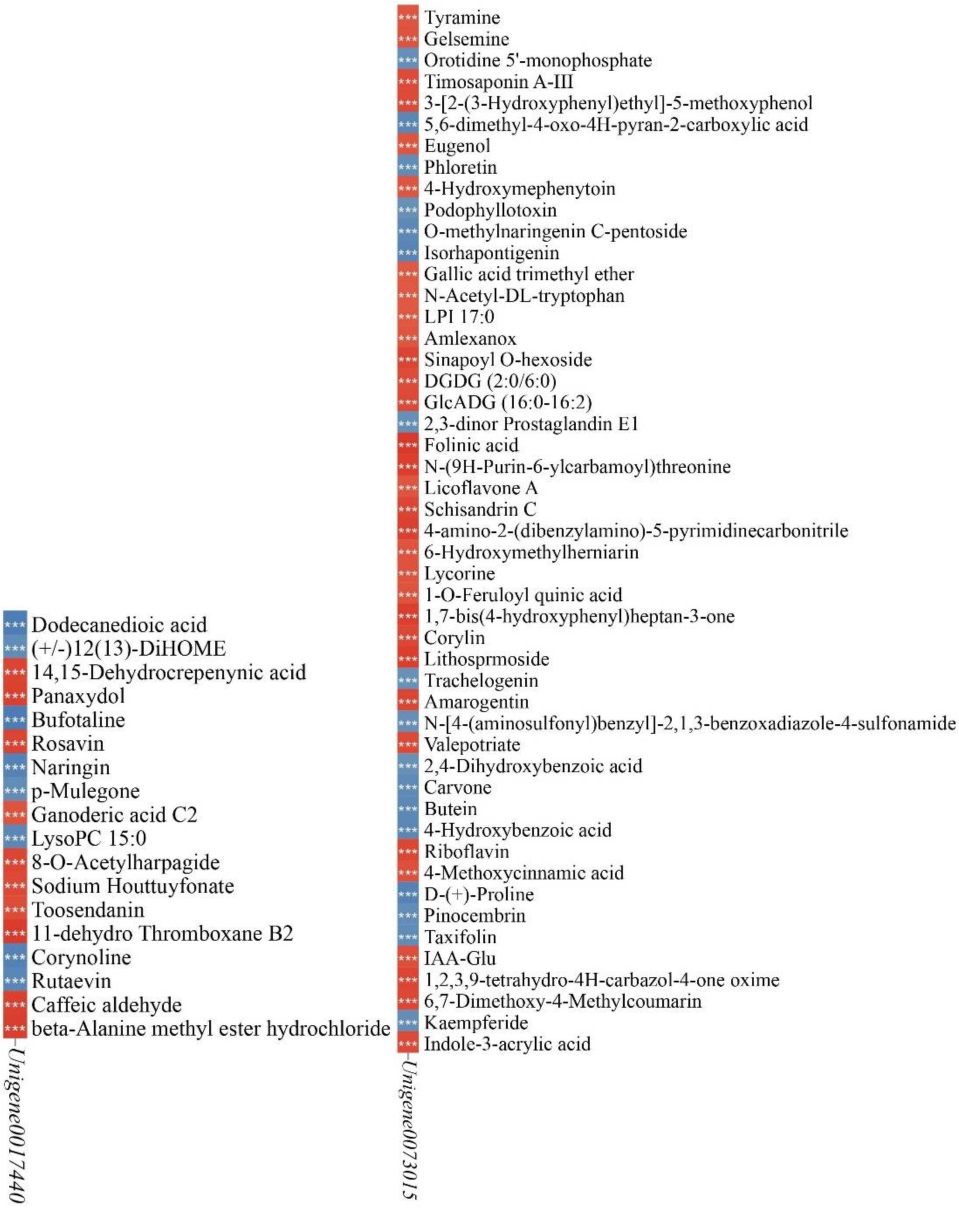
Dodecanedioic acid
(+/-)12(13)-DiHOME
14,15-Dehydrocrepenynic acid
Panaxydol
Bufotaline
Rosavin
Naringin
p-Mulegone
Ganoderic acid C2
LysoPC 15:0
8-O-Acetylharpagide
Sodium Houttuyfonate
Toosendanin
11-dehydro Thromboxane B2
Corynoline
Rutaevin
Caffeic aldehyde
beta-Alanine methyl ester hydrochloride
Unigene0017440
Tyramine
Gelsemine
Orotidine 5'-monophosphate
Timosaponin A-III
3-[2-(3-Hydroxyphenyl)ethyl]-5-methoxyphenol
5,6-dimethyl-4-oxo-4H-pyran-2-carboxylic acid
Eugenol
Phloretin
4-Hydroxymephenytoin
Podophyllotoxin
O-methylnaringenin C-pentoside
Isorhapontigenin
Gallic acid trimethyl ether
N-Acetyl-DL-tryptophan
LPI 17:0
Amlexanox
Sinapoyl O-hexoside
DGDG (2:0/6:0)
GlcADG (16:0-16:2)
2,3-dinor Prostaglandin E1
Folinic acid
N-(9H-Purin-6-ylcarbamoyl)threonine
Licoflavone A
Schisandrin C
4-amino-2-(dibenzylamino)-5-pyrimidinecarbonitrile
6-Hydroxymethylherniarin
Lycorine
1-O-Feruloyl quinic acid
1,7-bis(4-hydroxyphenyl)heptan-3-one
Corylin
Lithosprmoside
Trachelogenin
Amarogentin
N-[4-(aminosulfonyl)benzyl]-2,1,3-benzoxadiazole-4-sulfonamide
Valepotriate
2,4-Dihydroxybenzoic acid
Carvone
Butein
4-Hydroxybenzoic acid
Riboflavin
4-Methoxycinnamic acid
D-(+)-Proline
Pinocembrin
Taxifolin
IAA-Glu
1,2,3,9-tetrahydro-4H-carbazol-4-one oxime
6,7-Dimethoxy-4-Methylcoumarin
Kaempferide
Indole-3-acrylic acid
Unigene0073015

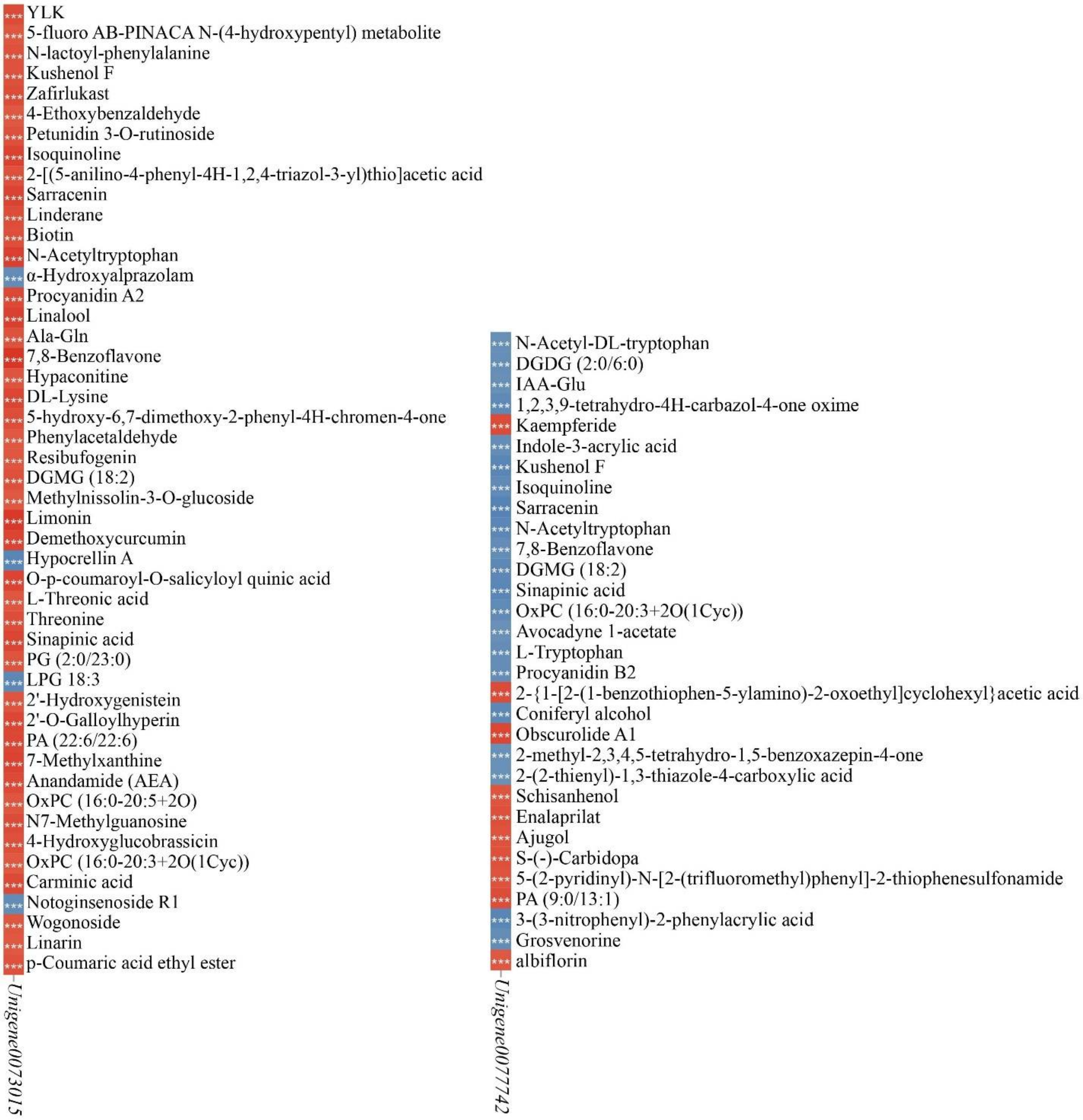
YLK
5-fluoro AB-PINACA N-(4-hydroxypentyl) metabolite
N-lactoyl-phenylalanine
Kushenol F
Zafirlukast
4-Ethoxybenzaldehyde
Petunidin 3-O-rutinoside
Isoquinoline
2-[(5-anilino-4-phenyl-4H-1,2,4-triazol-3-yl)thio]acetic acid
Sarracenin
Linderane
Biotin
N-Acetyltryptophan
α-Hydroxyalprazolam
Procyanidin A2
Linalool
Ala-Gln
7,8-Benzoflavone
Hypaconitine
DL-Lysine
5-hydroxy-6,7-dimethoxy-2-phenyl-4H-chromen-4-one
Phenylacetaldehyde
Resibufogenin
DGMG (18:2)
Methylnissolin-3-O-glucoside
Limonin
Demethoxycurcumin
Hypocrellin A
O-p-coumaroyl-O-salicyloyl quinic acid
L-Threonic acid
Threonine
Sinapinic acid
PG (2:0/23:0)
LPG 18:3
2'-Hydroxygenistein
2'-O-Galloylhyperin
PA (22:6/22:6)
7-Methylxanthine
Anandamide (AEA)
OxPC (16:0-20:5+2O)
N7-Methylguanosine
4-Hydroxyglucobrassicin
OxPC (16:0-20:3+2O(1Cyc))
Carminic acid
Notoginsenoside R1
Wogonoside
Linarin
p-Coumaric acid ethyl ester
Unigene0073015
N-Acetyl-DL-tryptophan
DGDG (2:0/6:0)
IAA-Glu
1,2,3,9-tetrahydro-4H-carbazol-4-one oxime
Kaempferide
Indole-3-acrylic acid
Kushenol F
Isoquinoline
Sarracenin
N-Acetyltryptophan
7,8-Benzoflavone
DGMG (18:2)
Sinapinic acid
OxPC (16:0-20:3+2O(1Cyc))
Avocadyne 1-acetate
L-Tryptophan
Procyanidin B2
2-{1-[2-(1-benzothiophen-5-ylamino)-2-oxoethyl]cyclohexyl}acetic acid
Coniferyl alcohol
Obscurolide A1
2-methyl-2,3,4,5-tetrahydro-1,5-benzoxazepin-4-one
2-(2-thienyl)-1,3-thiazole-4-carboxylic acid
Schisanhenol
Enalaprilat
Ajugol
S-(-)-Carbidopa
5-(2-pyridinyl)-N-[2-(trifluoromethyl)phenyl]-2-thiophenesulfonamide
PA (9:0/13:1)
3-(3-nitrophenyl)-2-phenylacrylic acid
Grosvenorine
albiflorin
Unigene0077742

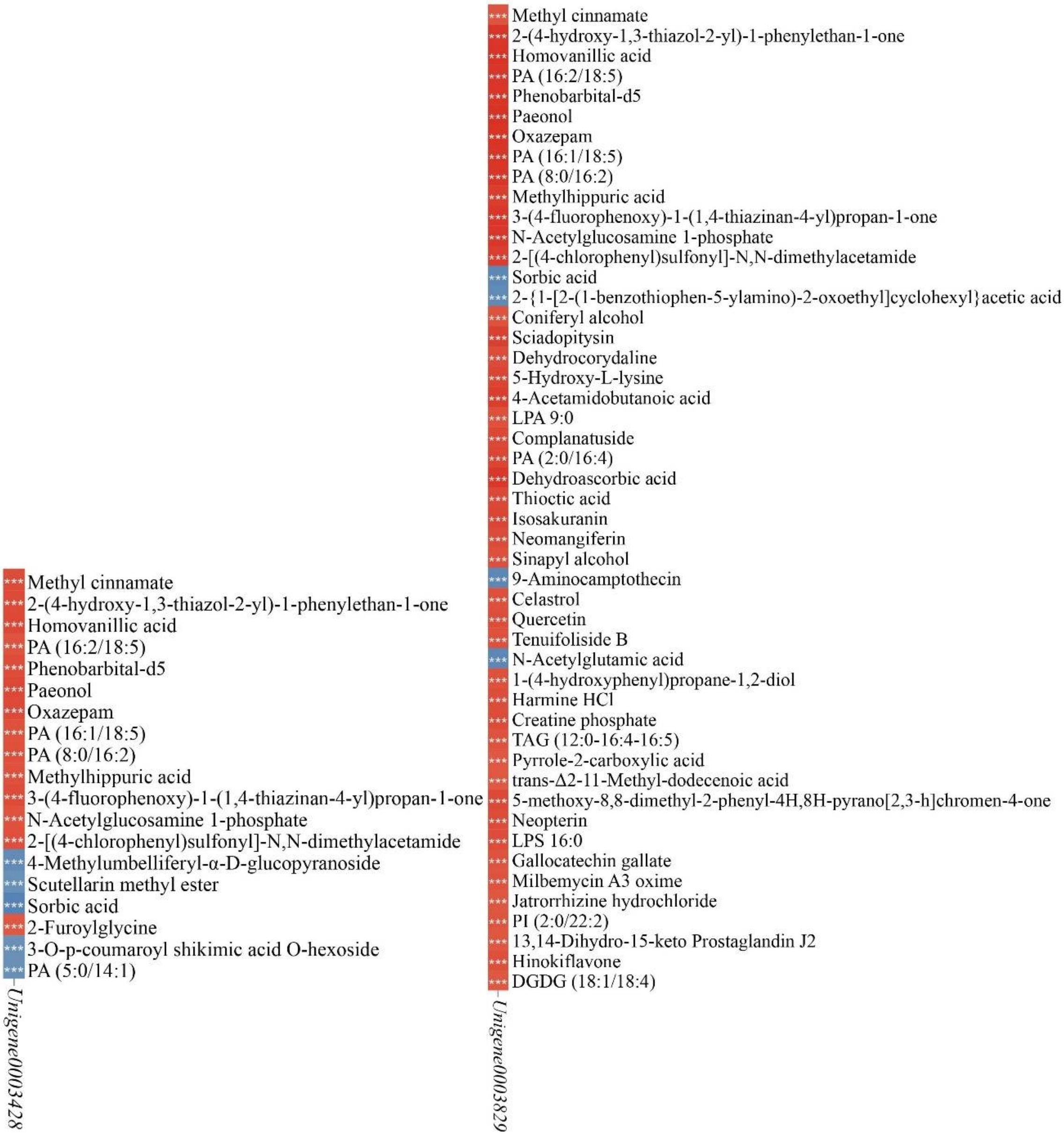
Methyl cinnamate
2-(4-hydroxy-1,3-thiazol-2-yl)-1-phenylethan-1-one
Homovanillic acid
PA (16:2/18:5)
Phenobarbital-d5
Paeonol
Oxazepam
PA (16:1/18:5)
PA (8:0/16:2)
Methylhippuric acid
3-(4-fluorophenoxy)-1-(1,4-thiazinan-4-yl)propan-1-one
N-Acetylglucosamine 1-phosphate
2-[(4-chlorophenyl)sulfonyl]-N,N-dimethylacetamide
4-Methylumbelliferyl-α-D-glucopyranoside
Scutellarin methyl ester
Sorbic acid
2-Furoylglycine
3-O-p-coumaroyl shikimic acid O-hexoside
PA (5:0/14:1)
Unigene0003428
Methyl cinnamate
2-(4-hydroxy-1,3-thiazol-2-yl)-1-phenylethan-1-one
Homovanillic acid
PA (16:2/18:5)
Phenobarbital-d5
Paeonol
Oxazepam
PA (16:1/18:5)
PA (8:0/16:2)
Methylhippuric acid
3-(4-fluorophenoxy)-1-(1,4-thiazinan-4-yl)propan-1-one
N-Acetylglucosamine 1-phosphate
2-[(4-chlorophenyl)sulfonyl]-N,N-dimethylacetamide
Sorbic acid
2-{1-[2-(1-benzothiophen-5-ylamino)-2-oxoethyl]cyclohexyl}acetic acid
Coniferyl alcohol
Sciadopitysin
Dehydrocorydaline
5-Hydroxy-L-lysine
4-Acetamidobutanoic acid
LPA 9:0
Complanatuside
PA (2:0/16:4)
Dehydroascorbic acid
Thioctic acid
Isosakuranin
Neomangiferin
Sinapyl alcohol
9-Aminocamptothecin
Celastrol
Quercetin
Tenuifoliside B
N-Acetylglutamic acid
1-(4-hydroxyphenyl)propane-1,2-diol
Harmine HCl
Creatine phosphate
TAG (12:0-16:4-16:5)
Pyrrole-2-carboxylic acid
trans-Δ2-11-Methyl-dodecenoic acid
5-methoxy-8,8-dimethyl-2-phenyl-4H,8H-pyrano[2,3-h]chromen-4-one
Neopterin
LPS 16:0
Gallocatechin gallate
Milbemycin A3 oxime
Jatrorrhizine hydrochloride
PI (2:0/22:2)
13,14-Dihydro-15-keto Prostaglandin J2
Hinokiflavone
DGDG (18:1/18:4)
Unigene0003829

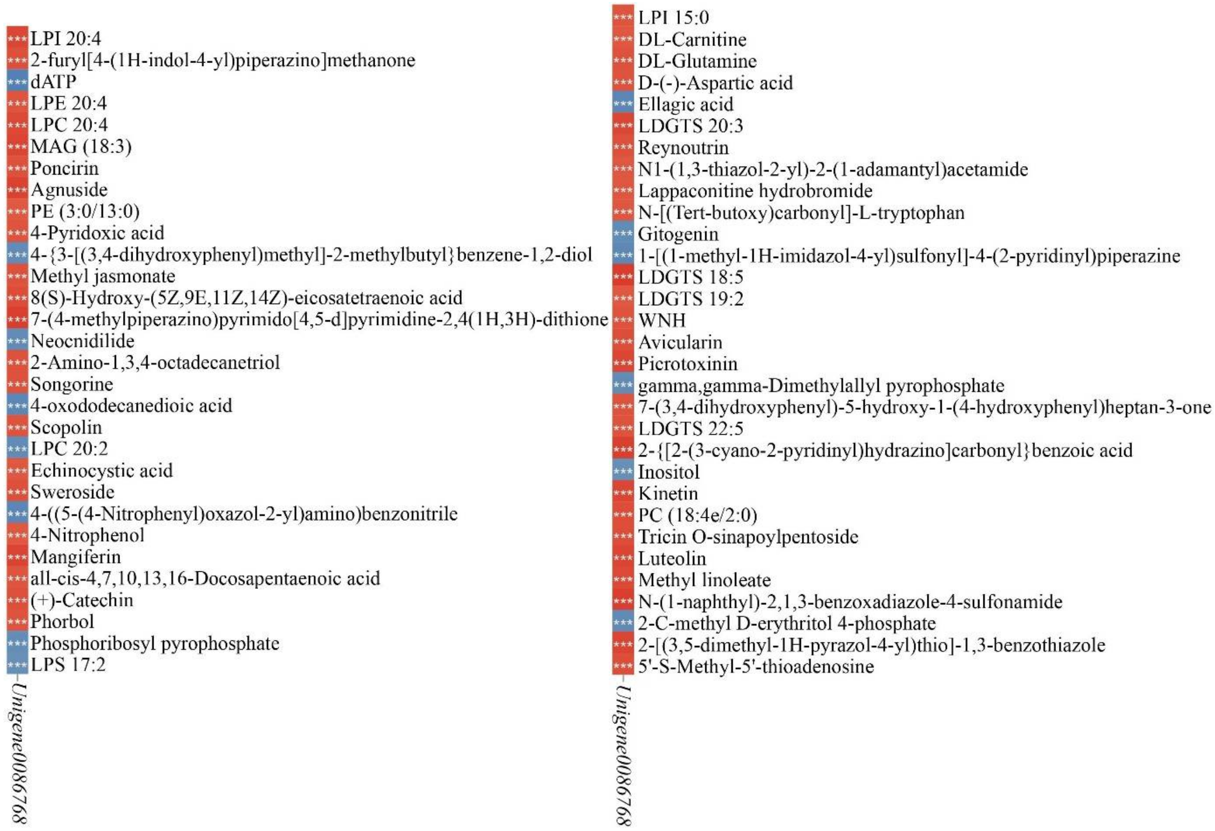

Supplementary Figure S6. Heatmap of correlations between major DGEs and metabolites in the CPAs transporter family

(According to the requirement of the absolute value of Person correlation coefficient |Corr|>0.8, the related DEGs and metabolome data in the CPAs transporter family were screened and correlated. $p \geq 0.05$ is not marked; $0.01 < p < 0.05$ is marked as *; $0.001 < p < 0.01$ is marked as **; $p \leq 0.001$ is marked as ***.)
