# Supplementary material for: Transcriptome and Metabonomic Analysis of Tamarix ramosissima Potassium (K+) Channels and Transporters in Response to NaCl Stress
Source: Genes (Basel). 2022 Jul 23;13(8):1313. doi: 10.3390/genes13081313 (PMC9394374; doi:10.3390/genes13081313)
Supplement: Supplementary file 1 [file genes-13-01313-s001.zip › Supplementaty Table S1.pdf]

Supplementary Table S1. Filtered reads quality statistics

Sample	Raw data (bp)	Clean data (bp)	Q20 (%)	Q30 (%)	GC (%)
CK-0h-1	6463611900	6358880951	97.56	93.13	45.04
CK-0h-2	6462481500	6308523705	97.53	93.09	45.24
CK-0h-3	6275665500	6169250017	97.62	93.30	45.06
NaCl-48h-1	5646281400	5524761130	98.80	96.02	44.95
NaCl-48h-2	6556220100	6447264123	98.75	95.84	44.92
NaCl-48h-3	6726009900	6617359533	98.73	95.77	44.89
NaCl-168h-1	6572502600	6447580283	98.60	95.50	45.04
NaCl-168h-2	6603229500	6484753576	98.72	95.83	44.75
NaCl-168h-3	6672482400	6555014485	98.69	95.72	44.98
NaCl+KCl-48h-1	6323610900	6208449917	97.57	93.18	45.30
NaCl+KCl-48h-2	6348257700	6220404531	97.65	93.38	45.26
NaCl+KCl-48h-3	6412534800	6279374482	97.63	93.30	45.26
NaCl+KCl-168h-1	6315612000	6203678507	97.65	93.39	45.01
NaCl+KCl-168h-2	6313731900	6189064447	97.58	93.24	45.05
NaCl+KCl-168h-3	6464712900	6343031296	97.16	92.27	44.99

Note: CK means control group; NaCl means 200 mM NaCl treatment group; NaCl + KCl means 200 mM NaCl + 10 mM KCl treatment group.
